# Systematically developed, comprehensive atlas of unique evidence-based psychological interventions for severe mental disorders

**DOI:** 10.1136/bmjment-2026-302555

**Published:** 2026-07-15

**Authors:** Giuliano Tomei, Chrysanthi Blithikioti, Camilla Cadorin, Lorena Pizzocri, Fabrizio Visconti, Marcella Lucente, Irene Gómez-Gómez, Ioana Alina Cristea

**Affiliations:** 1 Department of General Psychology, Università degli Studi di Padova, Padova, Italy; 2 Universidad de Sevilla, Seville, Spain

**Keywords:** Psychotherapy, Psychotic Disorders, Borderline Personality Disorder, Anorexia Nervosa, Bipolar and Related Disorders

## Abstract

**Background:**

The existent classifications of the distinct evidence-based psychological interventions (EBPs) are broad, not systematically developed or restricted to one mental disorder.

**Objective:**

To identify the unique EBPs and their variations evaluated for six severe mental disorders (schizophrenia and psychosis, bipolar disorders, bulimia nervosa, anorexia nervosa, one type of substance use and borderline personality).

**Study selection and analysis:**

We established a large cohort of EBPs by selecting all randomised controlled trials (RCTs) from six recent and large network meta-analyses for severe mental disorders in adults. Pairs of researchers independently selected all psychological intervention arms and assigned them to macro-families based on labels and theoretical background. Subsequently, within each macro-family, groups of identical arms, that is, referencing the same manual or protocol, were consolidated as distinct psychological interventions. Adaptations related to content, structure, delivery or culture were also described.

**Findings:**

From 260 RCTs, 422 psychological intervention arms were grouped in 45 macro-families and reduced to 266 distinct interventions (63% of total arms) across disorders. Intervention variability, that is, the proportion of unique psychological interventions out of the total number of arms, was lowest for the substance use disorder selected—stimulant use disorder (47/104, 45%) and bulimia nervosa (25/45, 55.5%). Variability was high for bipolar disorders (48/59, 81%), anorexia nervosa (20/29, 69%), schizophrenia/psychosis (86/123, 70%) and borderline personality disorder (40/62, 64.5%). 79 of the 266 distinct interventions (29%) contained adaptations (12% multiple adaptations) and 75/266 (28%) had openly available manuals or protocols.

**Conclusions and clinical implications:**

Mapping EBPs across macro-families and distinct interventions could inform evidence synthesis and identification of active ingredients, guide mechanisms of change exploration and support prioritising interventions for research and dissemination.

**WHAT IS ALREADY KNOWN ON THIS TOPIC**

- The distinct evidence-based psychotherapies and variations tested for each mental disorder have never been systematically charted. Existing classifications of psychotherapies had a narrow focus on one disorder or approach, and most were not developed systematically, as for example from a comprehensive list of interventions tested in randomised controlled trials.

**WHAT THIS STUDY ADDS**

- Across 260 randomised trials of psychological interventions sourced from six large network meta-analyses for severe mental disorders (schizophrenia and psychosis, bipolar disorders, bulimia nervosa, anorexia nervosa, stimulant use disorder and borderline personality disorder), we identified 422 psychological treatment arms, mapped onto 45 macro-families and distilled into 266 unique interventions. Variability, the proportion of unique psychological interventions out of the total number of psychological arms, was high (over 60%) for all disorders except stimulant use disorder and bulimia nervosa. 29% of the singular interventions included at least one adaptation, and 28% had a publicly accessible protocol or manual.

**HOW THIS STUDY MIGHT AFFECT RESEARCH, PRACTICE OR POLICY**

- Mapping treatment protocols and manuals into macro-families and distinct interventions across disorders could represent a starting point for identifying active treatment components, as well as for optimising and prioritising interventions for research and dissemination, for example, favouring interventions with openly accessible manuals, particularly in low- and middle-income settings. The proposed classification could support a more precise aggregation of intervention arms in evidence synthesis and better trial planning and reporting by providing researchers with an atlas from which to select the treatments evaluated or delivered.

## Introduction

Evidence-based psychotherapies (EBPs) for mental disorders are evaluated in randomised controlled trials (RCTs) and subsequent meta-analyses of these and widely recommended by clinical practice guidelines (CPGs). The content and delivery of these interventions are described in treatment protocols and manuals, analogous to drug labels that specify ingredients, dosage and administration. Nevertheless, the distinct EBPs and variations tested for each mental disorder have never been charted. The question has wide-ranging implications, beyond mere accounting.

First, presently, meta-analyses frequently lump together interventions from the same family (eg, cognitive–behavioural therapy (CBT)), which increases unexplained heterogeneity^1^ and precludes investigating if some versions in the same family are more effective than others, generally or in pre-specified patient subgroups. Based on such meta-analyses, CPGs often make broad strokes recommendations about entire intervention classes, instead of specific protocols. Conversely, with a comprehensive atlas of distinct psychological interventions grouped by disorder, meta-analysts could only aggregate those identical and examine differences among the others. Second, distinct interventions within the same family may differ in terms of accessibility and scalability. Some manuals might only be commercially available,^2^ older, more difficult to retrieve,^3^ require more intensive delivery or highly specialised professionals, thus rendering interventions using them more difficult to scale. Precise information about distinct interventions could help CPG panels prioritise those easier to implement or more scalable, at the very least in contexts of limited resources. Third, for some interventions, such as dialectical behaviour therapy (DBT) for symptoms of borderline personality disorder, trials have been conducted over more decades.^4 5^ When pooling older and more recent trials in a meta-analysis, it would be important to ascertain if the same intervention protocol was delivered or whether there are adaptations, for example, in content, that could be associated with changes in efficacy.

Though some professional societies such as the American Psychological Association maintain updated lists of EBPs by disorders (eg, https://societyofclinicalpsychology.org/resources/psychological-treatments/), the methodology of selecting interventions is non-systematic and the list may not include all distinct interventions tested in RCTs. An alternative approach could rely on network meta-analyses (NMAs), which assemble large collections of trials, with diverse comparators, allowing for representation of both frequently and infrequently studied interventions. NMAs have been labelled as the highest level of evidence in treatment guidelines^6^ and are increasingly used by organisations like the National Institute for Health and Care Excellence in producing such guidelines.^7^ We report a systematic mapping of unique psychological interventions, including any adaptations, tested in randomised trials retrieved from recent NMAs across six severe mental disorders in adults.

## Methods

We assembled a large cohort of RCTs of psychological interventions for severe mental disorders in adults, relying on recent, large NMAs. The search and selection process of the NMAs for each disorder was described in a previous report^3^ and reporting adhered to the Preferred Reporting Items for Systematic Reviews and Meta-Analyses (PRISMA) 2020 guidelines,^8^ insofar as feasible. Briefly, we searched PubMed as of November 2023 for NMAs published over the previous 5 years (since 2018) of psychological interventions for six severe mental disorders, specifically schizophrenia and psychotic disorders, bipolar disorders, bulimia nervosa, anorexia nervosa, one type of substance use disorder (SUD) and borderline personality disorder. The search and selection process for each NMA was previously described^3^ and is presented in online supplemental table S1. For each disorder, only one NMA was selected, prioritising number of included trials, recency (if similar number of trials) and inclusion of psychological interventions as stand-alone versus adjunctive interventions (when available). For SUD, there was no comprehensive NMA for more types of substances, so we selected an NMA for one type of substance (stimulants, specifically cocaine and amphetamine). The characteristics of the selected NMAs are displayed in online supplemental table S2.

10.1136/bmjment-2026-302555.supp1 Supplementary data

From each of the six NMAs,^9–14^ we selected all included RCTs and for each RCT, all active psychological intervention arms, defined as including at least one psychological component and delivered in the same way to all participants (eg, following a protocol or manual), regardless of whether trialists had originally intended them as control conditions. However, we did not include treatment as usual (TAU) or enhanced TAU, even when their description mentioned psychological components, as delivery of these could vary significantly across trial sites or even participants in the same site. Non-pharmacological interventions, even when presumed to work through psychological mechanisms (eg, exercise), were also excluded. Pairs of researchers working independently (GT, CB, LP, IG-G, CC, ML, FV) identified active psychological arms for each disorder, with disagreements resolved by consultations with the senior author (IAC).

For each disorder, pairs of researchers (GT, CB, LP, IG-G, CC, ML, FV) independently assigned all identified psychological interventions to macro-families (eg, CBT, psychodynamic psychotherapy, DBT), using a bespoke Excel spreadsheet. The process was inductive and did not start from a predefined list across disorders. The macro-family was identified based on the label and theoretical background of the intervention, as described in the NMA from which the trial had been selected and in the original trial report. We aimed for macro-families to include interventions that explicitly adhered to a common theoretical background but avoided making assumptions when the background was unclear or eclectic (ie, combining elements from more theories). We considered cases with unclear or eclectic background as separate families. Interventions that combined two approaches (eg, schema therapy and DBT) or merged components originating from distinct macro-families (eg, psychodynamic and cognitive) were assigned to up to three separate macro-families. Each pair of researchers first identified the macro-families for the disorder assigned. Next, all pairs together revised assignments for interventions not clearly labelled or with other doubts about the assignment.

Subsequently, from all psychological intervention arms within a macro-family and a disorder, the same pairs of researchers independently identified groups of interventions that referenced the same protocol or manual in the description in the trial reports. These groups were consolidated into unique interventions. When intervention descriptions in trial reports cited multiple (ie, manual, another article) or divergent sources (eg, articles or manuals from different years) for similarly labelled interventions developed by the same author group, we consulted all these sources to see if a common progenitor manual could be identified and if there were indications it was modified. Interventions for which a common progenitor manual could be identified, with no indication of substantive modifications, were consolidated under the same label.

The same pairs of independent reviewers also recorded when changes to the intervention manual or protocol were described in the trial reports. These were considered adaptations if the core manual remained the same, but authors described specific modifications. We considered adaptations related to content (eg, adding or removing components, specific populations), structure (eg, number or frequency of sessions), delivery format (eg, group, digital) and culture (only if the change went beyond simple translation, eg, involving modifications due to cultural or religious norms and values^15^). A detailed description of adaptation types in each category is included in online supplemental table S3. Conversely, if changes described were substantive, for example, integrating elements from more therapy approaches, describing novel therapeutic targets or multiple modifications to intervention components, we considered the intervention as distinct (ie, not an adaptation of an already consolidated intervention). For insufficiently described adaptations (eg, ‘adapted from’ with no further details), we first cross-checked intervention structure, delivery and content by comparing protocols or manuals with what was described in the paper to assess whether we could infer specific adaptations (eg, paper stating eight sessions compared with manual stating 21). Modifications of the original manual that could not be assigned to one of the predefined categories were classified as ‘unknown’. Interventions that did not reference a specific manual were also considered distinct, owing to the impossibility of reproducing them. A senior researcher (IAC), not involved in the original coding, reviewed all assignments to macro-families, consolidation into unique interventions and adaptations. Disagreements were resolved through multiple rounds of discussion among each pair of raters and with the senior researcher (IAC).

The resultant list of distinct interventions was cross-checked with a previously published,^3^ openly available dataset (10.5281/zenodo.14567988) regarding accessibility and public availability of treatment protocols and manuals for the same trials and psychological treatment arms studied here. In this previous work,^3^ we sought comprehensive intervention descriptions, such as protocols or manuals, for all active psychological arms examined here. Here, we report the number of unique interventions in each macro-family that were (1) unpublished and (2) openly available (for mixed interventions if at least one of the component interventions had a publicly available manual).

Results are presented descriptively, as counts and proportions, computed in Microsoft Excel (Office Professional Plus 2021). As all intervention arms with psychological components are described, we do not report CIs. For each disorder, we computed intervention variability, defined as the proportion of unique psychological interventions out of the total number of psychological arms. To identify instances where the same manual was used in different disorders, Claude Opus 4.7 was used between 4 May and 7 May 2026 on the extracted datasets, with hits checked by two raters (GT, CB). Figure 2 was created using a data visualisation platform built on JavaScript (flourish.studio). Claude Opus 4.7 was used on 15 May 2026 to create an interactive visualisation of the unique intervention and corresponding manuals.

## Results

**Figure 1:**
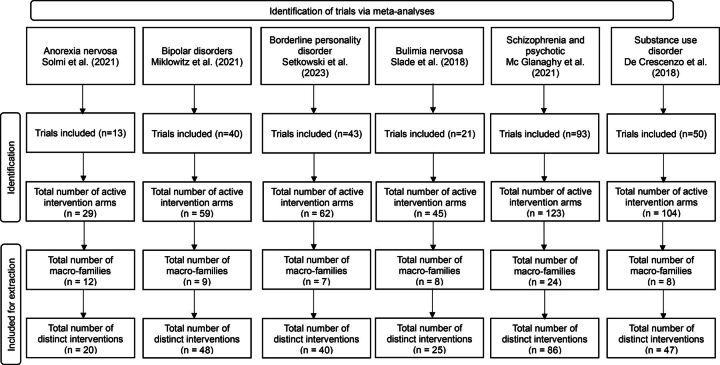
Flow diagram of identification of randomised clinical trials, psychological intervention arms, macro-families of interventions and distinct interventions.

Figure 1 displays a flow diagram, adapted from the PRISMA 2020, of the selection and identification of the intervention arms, as well as of the consolidation in macro-families and unique interventions.

**Figure 2:**
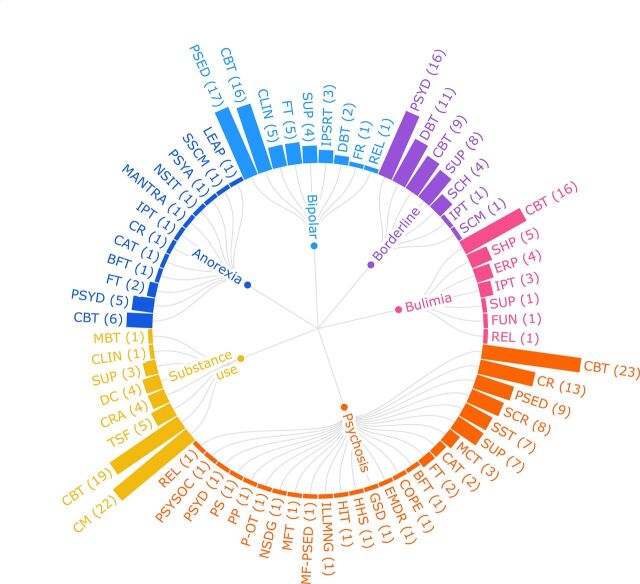
Graphical representation of family distribution across distinct interventions by disorder. Bar length is proportional to the frequency of each macro-family. Bracketed numbers indicate the corresponding absolute frequency. BFT, behavioural family therapy; CAT, cognitive analytic therapy; CBT, cognitive–behavioural therapy; CLIN, clinical intervention; CM, contingency management; COPE, coping skills; CR, cognitive remediation; CRA, community reinforcement approach; DBT, dialectical behaviour therapy; DC, drug counselling; EMDR, eye movement desensitisation and reprocessing; ERP, exposure and response prevention; FR, functional remediation; FT, family therapy; FUN, functional analysis; GSD, guided self-determination; HHS, holistic health sessions; HIT, hallucination-focused integrative treatment; ILLMNG, illness management; IPSRT, interpersonal and social rhythm therapy; IPT, interpersonal psychotherapy; LEAP, compulsive exercise activity therapy; MANTRA, Maudsley model of anorexia nervosa treatment for adults; MBT, mind-body therapy; MCT, metacognitive training; MF-PSED, mindfulness-based psychoeducation; MFT, multifamily treatment; NSDG, non-specific discussion group; NSIT, non-specialistic intervention; P-OT, psychological-based occupational therapy; PP, positive psychotherapy; PS, problem solving; PSED, psychoeducation; PSYA, psychoanalysis; PSYD, psychodynamic psychotherapy; PSYSOC, psychosocial intervention; REL, relaxation; SCH, schema therapy; SCM, structured clinical management; SCR, social cognitive remediation; SHP, self-help; SSCM, specialistic supportive clinical management; SST, social skills training; SUP, supportive therapy; TSF, twelve-step facilitation.

The six NMAs included 260 trials from which we identified 422 psychological intervention arms, grouped in 45 distinct macro-families and consolidated in 266 unique interventions (63% of total psychological arms) across disorders. A complete list of macro-families, total psychological intervention arms, distinct interventions and publicly accessible distinct interventions across all six disorders is presented in online supplemental table S4. Broadly, we identified 12 macro-families for anorexia nervosa, 9 for bipolar disorder, 7 for borderline personality disorder, 7 for bulimia nervosa, 25 for schizophrenia and psychosis and 8 for SUD. A visual display of the macro-families by disorder is presented in figure 2. Across all disorders, CBT was the most common macro-family by absolute number of unique interventions of the total (89/134, 66%), followed by psychoeducation (26/37, 70%), supportive therapy (23/26, 88%), psychodynamic psychotherapy (22/27, 81%) and contingency management (22/60, 37%), though the latter appeared exclusively for SUD. Of the most common macro-families, manuals were publicly available for 20/89 CBT (22%), 5/23 supportive (22%) and 2/22 psychodynamic distinct interventions (9%). 22 macro-families included only one unique intervention each. Online supplemental tables S5–S10 include, for each disorder, the list of original intervention arms in each trial, the macro-families assigned, the unique interventions and any adaptations. The datasets of all distinct psychological intervention arms by disorder are publicly available on Zenodo,^16^ and an interactive visualisation of the macro-families and unique interventions is available at https://ioanaalina.github.io/Psychotherapy-atlas/psychotherapy_atlas(2).html. 39 distinct interventions had a mixed theoretical background and were included in more than one macro-family (online supplemental table S11). Seven manuals (3%) were transdiagnostic, that is, used across two or more disorders (online supplemental table S12).

Numbers of unique psychological interventions in each macro-family and disorder are displayed in online supplemental table S13, and absolute and relative frequencies of macro-families and unique interventions by disorder are presented in table 1. Intervention variability, that is, the proportion of unique psychological interventions out of the total number of psychological arms, was lowest for SUD (47/104, 45%) and bulimia nervosa (25/45 arms, 55.5%). Conversely, variability was high across interventions for bipolar disorder (48/59, 81%), anorexia nervosa (20/29, 69%), schizophrenia/psychosis (86/123, 70%) and borderline personality disorder (40/62, 64.5%).

**Table 1:** Macro-families and unique interventions by disorder

Disorder	RCTs	Psy arms	Macro-families n (%*)	Unique† n (%*)
Anorexia nervosa	13	29	12 (41)	20 (69)
Bipolar disorder	40	59	9 (15)	48 (81)
Borderline personality disorder	43	62	7 (11)	40 (64.5)
Bulimia nervosa	21	45	7 (15.5)	25 (55.5)
Schizophrenia and psychosis	93	123	25 (20)	86 (70)
Substance use disorder	50	104	8 (8)	47 (45)
Total	260	422	45 (11)‡	266 (63)

*Out of the disorder’s total number of psychological intervention arms.

†Number of distinct interventions.

‡Total macro-families across all disorders; macro-families appearing in multiple disorders were counted once in the total.

Psy, psychological intervention; RCTs, randomised controlled trials.

Characteristics of unique psychological intervention in each disorder class are presented in table 2. Of the total 266 distinct interventions, 79 (29%) contained at least one adaptation and 32 distinct (12%) incorporated multiple modifications to the original manual or protocol. We identified a total of 137 adaptations (online supplemental table S14), most of which involved content (59/137, 43%) or structure (42/137, 31%). Most content adaptation involved a modified component (46/59) or changes for a specific population (12/59). Most structure adaptations regarded changes to the number of sessions (30/42), and others involved changes to session length (9), frequency (2) or internal structure (1). Modifications related to delivery (14/137, 10%) and cultural elements (9/134, 7%) were rare. Delivery adaptations referred to a modified format (8/14), delivery mode (4) or setting change (1). Cultural adaptations (online supplemental table S15) frequently only described the culture to which the protocol was updated. Nine adaptations (7%) were described elliptically or ambiguously and labelled ‘unknown’.

**Table 2:** Characteristics of unique interventions grouped by disorders

Disorder	Unique*	Mixed n (%)†	Open access n (%)‡	Non-manualised n (%)§	Unpublished n (%)¶	With adaptions n (%)**	With multiple adaptions n (%)††
Anorexia nervosa	20	2 (10)	3 (15)	4 (20)	4 (20)	7 (35)	0
Bipolar disorders	48	6 (12.5)	17 (35)	3 (6)	12 (25)	12 (25)	11 (23)
Borderline personality disorder	40	9 (22.5)	5 (12.5)	2 (5)	14 (35)	13 (32.5)	9 (22.5)
Bulimia nervosa	25	6 (24)	5 (20)	1 (4)	5 (8)	17 (68)	2 (8)
Schizophrenia and psychosis	86	4 (5)	27 (31)	0	21 (24)	19 (22)	6 (7)
Substance use disorders	47	12 (25.5)	18 (38)	4 (8.5)	14 (30)	11 (23)	4 (8.5)
Total	266	39 (15)	75 (28)	14 (5)	70 (26)	79 (30)	32 (12)

*Number of distinct interventions.

†Mixed theoretical background.

‡Interventions have a publicly available protocol/manual (for combined interventions, the protocol/manual for at least one of the interventions is openly available).

§Intervention is not manualised.

¶Intervention manual is unpublished.

**Interventions with at least one adaptation.

††Interventions with multiple adaptations.

Of the 266 distinct psychological interventions, 14 were not manualised (5%), and 70 (26%) were described in unpublished manuals, resulting in 84 (31%) unique interventions that could not be directly accessed without contacting intervention developers. 75 distinct interventions (28%) had an openly available protocol or manual.

## Discussion

Across 260 trials of psychological interventions for six severe mental disorders, we identified 422 psychological treatment arms, which were mapped onto 45 macro-families and distilled into 266 unique interventions. Almost a third of the singular interventions included at least one adaptation, and 12% included multiple adaptations. One-third of the interventions were either not manualised or with an unpublished manual, and approximately one-third had a publicly, openly, accessible protocol or manual. Most distinct interventions were concentrated within CBT, supportive and psychodynamic approaches, and just around a fifth of these had open-access protocols or manuals. Contingency management and psychoeducation were also among the most common families of unique interventions, though this result was largely driven by their extensive use in bipolar disorders and SUD.

Our results provide a systematic, cross-disorder mapping of unique evidence-based psychological interventions for six severe mental disorders in adults. Psychological interventions were evaluated in trials included in NMAs, indicating that they contributed, directly or indirectly, to evidence synthesis. Our results cannot be extrapolated to estimate the total number of distinct manualised psychotherapies. One recent scoping review^17^ identified 1132 book-based English language treatment manuals considering all adult mental disorders. Only 151 of these (13.3%) were explicitly mentioned in evidence-based CPGs. We identified 266 distinct interventions for six severe mental disorders, not including common disorders like depression or anxiety, which also implied we found few transdiagnostic manuals (3%). Conversely, most manuals identified by the scoping review (71%) had at least one transdiagnostic target. The research question and approach significantly diverge between our work and the scoping review by Wislocki and coauthors.^17^ We aimed to distinguish interventions that had been tested in RCTs and subsequently included in evidence synthesis. It is possible that not all these interventions were recognised in CPGs, but they all informed evidence synthesis and were formally evaluated in trials. Additionally, we also considered non-book-based treatment descriptions, such as protocols developed within a trial. Conversely, Wislocki *et al*^17^ sought to identify all book-based therapy manuals, whether these were tested or not in trials, and subsequently looked if these manuals had been cited in CPGs and at what level of evidence.

The existence of a vast array of psychological interventions, several of which contain adaptations, is valuable in providing choices for patients and in diverse clinical contexts. However, the variability across interventions complicates assessment of effectiveness. Meta-analyses incorporate this heterogeneity by lumping interventions in broad classes, assuming similar effects and mechanisms for treatments in the same class. Aggregating across classes obscures the distinctions among interventions in the same class. However, parsing variability is key for personalising treatment, that is, within the same class, some versions might be more effective or acceptable for some patient subgroups. Our findings could support a more granular approach of consolidating interventions for evidence synthesis, mapping them onto unique interventions or macro-families, which could then be used to define direct comparisons in a pairwise meta-analysis or as nodes in an NMA.

About a third of unique interventions identified contained adaptations to the original manual, in contrast to a recent scoping review^17^ that found only 10% of book-based treatment manuals contained adaptations. The discrepancy could be accounted for by the fact that adapted interventions are more likely to be developed in the context of a new trial and described in a protocol or supplement than a new book. More generally, there is little research as to whether such adaptations serve their purpose of optimising the intervention for a specific population or clinical context. Most modifications related to content (eg, not delivering some component included in the original manual), and fewer modifications related to structure (eg, modifying number of sessions or delivery mode). Cultural adaptations were sporadically tested, as previously shown for common mental disorders in people of Chinese descent.^15^ Our list of interventions with adaptations could be used to identify those with higher optimisation potential that could be tested against the original intervention in comparative trials.

Macro-families, derived from how the authors labelled the intervention and described its theoretical foundations, could provide important indications for identifying mechanisms of change. Interventions within the same macro-family frequently share commonalities and rely on similar theoretical constructs, which could be further operationalised in experimental and computational research. In identifying macro-families, we abided by the original language and theoretical underpinning of the treatments, rather than opting for a higher order classification. Other previous classification endeavours, such as the Nottingham Classification,^18^ developed for schizophrenia, aimed to group interventions in higher order classes, such as ‘Thought/Action’, ‘Cognitive functioning’, ‘Social’ and ‘Psychoanalytic/dynamic’. While pragmatically useful for evidence synthesis, a broad strokes, higher order classification risks obscuring important conceptual and technical differences between approaches in the same class. Our resultant macro-family classification, while significantly more extensive (45 vs 10 in the Nottingham), is also more precise, particularly in light of our inclusion of multiple, distinct disorders. However, half of the macro-families only included one intervention and are unlikely to contribute significantly to evidence synthesis. Across disorders, CBT was the most prevalent macro-family, followed by non-specific interventions (supportive and psychoeducation, used often for bipolar disorders), contingency management (used specifically for SUD) and psychodynamic approaches. Our classification also included interventions originally delivered as control if these contained psychological elements, which might at least partly explain the prominence of non-specific approaches. Such interventions were included as are often shown to have stand-alone benefits. For example, though general psychiatric management was intended as a control condition for DBT for borderline personality disorder symptoms, its efficacy was similar.^19^

Our findings have important implications for treatment implementation, dissemination and reporting. Overall, only 28% of the distinct interventions had open-access manuals, and this proportion was even lower for the best represented macro-families, such as CBT or psychodynamic approaches. Interventions with open manuals could be prioritised^20^ in effectiveness research and subsequently in implementation, particularly in low- and middle-income settings. From a macro-family of interventions shown to be effective, guidelines could also prioritise those with accessible manuals and deprioritise those with unpublished or not easily accessible manuals. For difficult to access interventions, guidelines could weigh whether the gain in efficacy is sufficient to justify the challenges in dissemination, when other interventions in the same macro-family exist. Similarly, from a macro-family for which meta-analyses provided evidence of effectiveness, interventions could be selected based on adaptations reducing treatment duration or requiring less specialised professionals for delivery. Moreover, patients who do not adhere or respond to one specific intervention could try another from the same macro-family without the need of changing therapist, which would often be the case if switching treatment class (eg, moving from CBT to psychodynamic). The classification in macro-families and distinct interventions could also support better reporting of interventions, as investigators could select these from our atlas, together with progenitor manuals and any adaptations. It could support more careful labelling of interventions, by using already existing distinct intervention names, as well as accurate referencing of manuals used, by cross-checking with the list we provide here.

Our analysis has several limitations. First, we sourced interventions from RCTs included in NMAs, so it is possible that some trials or interventions were missed. Nevertheless, by relying on NMAs, we ensure that most interventions that were tested and informed evidence synthesis were represented. Second, owing to the taxing nature of our analysis in terms of time and resources, we restricted our mapping to one NMA per disorder. Some of the excluded NMAs were restricted to one intervention family or delivery type or used non-clinical outcomes and hence would not have amended themselves to our methodology. Other excluded NMAs, particularly related to SUD and psychosis, potentially included additional distinct psychological interventions. For these cases, assessing overlap in terms of included trials was not practical due to multiple differences in the eligibility criteria between the selected and excluded NMAs. Third, the systematic search for NMAs was conducted in November 2023, so new NMAs for these disorders, with likely distinct eligibility criteria, could have been conducted in the mean time. While our approach charted unique evidence-based psychological interventions in a large cohort of trials, it is by no means exhaustive. Fourth, the classification in macro-families and distinct interventions is intertwined with how trial authors reported the interventions. We used independent raters and various rounds of consensus to reduce subjectivity in classification and attempted to balance sensitivity (ie, classifying interventions using different manuals as distinct) with precision (ie, avoiding creating new categories when an identical intervention was described in more trial reports). However, it is possible that we misclassified some interventions. Fifth, descriptions of interventions were sometimes limited and sparse in terms of theoretical orientation, which also could have led to misclassification. Sixth, we assigned composite and mixed interventions to up to three macro-families, depending on how these were described by trialists. From the standpoint of mechanisms of change and dissemination, such interventions pose important challenges, as they require the fusion of distinct theories and concepts and therapists specialised in more approaches. A macro-family labelled integrative approaches, while more accurate, would have missed the distinctive theoretical flavours within the category.

Our findings showcase the variability of psychological interventions across six severe mental disorders in adults, while also providing a systematisation of this variability in unique interventions and macro-families. The resultant classification of distinct interventions grouped in macro-families could represent a starting point for identifying active treatment components, as well as optimising and prioritising interventions for research and dissemination. Moreover, it could support better trial planning and reporting by providing an atlas that researchers could use when describing treatments delivered.

## Data Availability

Data are available in a public, open access repository. All the extracted data are available in the paper, supplement and in additional Excel files, including all active and unique interventions with references to all manuals, publicly accessible on Zenodo: doi:10.5281/zenodo.20203782. Interactive visualisations of the extracted data on unique interventions and manuals are also available on Zenodo (doi: 10.5281/zenodo.20203782) and at https://ioanaalina.github.io/Psychotherapy-atlas/psychotherapy_atlas(2).html.
