# Supplementary material for: Systematically developed, comprehensive atlas of unique evidence-based psychological interventions for severe mental disorders
Source: BMJ Ment Health. 2026 Jul 15;29(1):e302555. doi: 10.1136/bmjment-2026-302555 (PMC13386039; doi:10.1136/bmjment-2026-302555)
Supplement: Supplementary data [file bmjment-29-1-s001.pdf]

Appendix

Supplementary Methods 2

Table S1. Identification and selection of included network meta-analyses 2

Table S2. Characteristics of the included network meta-analyses 4

Table S3. Categories of adaptations and their definitions 5

Supplementary Results 6

Table S4. Intervention macro-families with total psychological intervention arms, consolidated distinct interventions and distinct interventions with publicly accessible manuals. 6

Table S5. Total psychological intervention arms with macro-family and adaptations – Anorexia Nervosa ..8

Table S6. Total psychological intervention arms with macro-family and adaptations – Bipolar disorder ...12

Table S7. Total psychological intervention arms with macro-family and adaptations – Borderline personality disorder 18

Table S8. Total psychological intervention arms with macro-family and adaptations – Bulimia nervosa ...28

Table S9. Total psychological intervention arms with macro-family and adaptations – Schizophrenia and psychotic 34

Table S10. Total psychological intervention arms with macro-family and adaptations – Substance use disorders (stimulant use disorders) 41

Table S11. Distinct interventions assigned to multiple families 55

Table S12. Manuals of distinct interventions used across more than one disorder 57

Table S13. Number of distinct intervention arms in each macro-family grouped by disorder 58

Table S14. Adaptations of interventions 60

Table S15. Cultural adaptations 61

Supplementary Methods

Table S1. Identification and selection of included network meta-analyses

Disorder	Search string	Hits	Eligible NMAs	Reasons for rejection
Psychosis	"network meta analysis"[Title/Abstract] AND ("psychotic"[Title/Abstract] OR "schizophrenia"[Title/Abstract]) AND ("psychol*"[Title/Abstract] OR "psychosocial*"[Title/Abstract] OR "psychother*"[Title/Abstract]) AND 2018/11/01:2023/11/01[Date - Publication]	10	4	1[1] on relapse prevention<br>1[2] on stigma<br>1[3] on a specific type of intervention
Borderline personality disorder	((network meta-analysis[Title/Abstract]) AND (borderline[Title/Abstract]) AND (psychol*[Title/Abstract] OR psychosocial*[Title/Abstract] OR psychother*[Title/Abstract])) AND (("2018/11/1"[Date - Publication] : "2023/11/1"[Date - Publication]))	2	1	Only one eligible hit
Substance-used disorder	"network meta-analysis"[Title/Abstract] AND ("alcohol"[Title/Abstract] OR "opioid"[Title/Abstract] OR "cannabis"[Title/Abstract] OR "hallucinogens"[Title/Abstract] OR "inhalants"[Title/Abstract] OR "sedatives"[Title/Abstract] OR "hypnotics"[Title/Abstract] OR "anxiolytics"[Title/Abstract] OR "stimulants"[Title/Abstract] OR "amphetamine"[Title/Abstract] OR "cocaine"[Title/Abstract] OR "substance"[Title/Abstract]) AND ("psychol*"[Title/Abstract] OR "psychosocial*"[Title/Abstract] OR "psychother*"[Title/Abstract]) AND 2018/11/01:2023/11/01[Date - Publication] [#]	14	7	1[4] harmful alcohol use, not alcohol use disorder<br>1[5] on opioid-dependent patients on methadone maintenance, smaller number of trials<br>1[6] smaller number of included trials<br>1[7] interventions for alcohol use, but population not alcohol use disorder<br>1[8] comorbid alcohol use and depression<br>1[9] included any intervention for maintaining abstinence, smaller number of trials for psychosocial
Bipolar disorder	"network meta analysis"[Title/Abstract] AND ("bipolar"[Title/Abstract] OR "mood"[Title/Abstract]) AND ("psychol*"[Title/Abstract] OR "psychosocial*"[Title/Abstract] OR "psychother*"[Title/Abstract]) AND 2018/11/01:2023/11/01 [Date - Publication]	16	2	1[10] on a specific type of intervention for depressive symptoms
Anorexia nervosa/bulimia nervosa	((network meta analysis[Title/Abstract]) AND (anorexia[Title/Abstract] OR bulimia[Title/Abstract] OR eating[Title/Abstract])) AND (("2018/11/01"[Date - Publication] : "2023/11/01"[Date - Publication]))[$]	2	2	1 eligible hit for each disorder

Note. NMA, Network meta-analysis

[#] Caffeine and tobacco use were excluded

[$] Terms related to the type of interventions were eliminated from the search string because no hits were initially retrieved.

Excluded references

1. Bighelli I, Rodolico A, García-Mieres H, et al. Psychosocial and psychological interventions for relapse prevention in schizophrenia: a systematic review and network meta-analysis. *Lancet Psychiatry* 2021;8(11):969–80. doi: 10.1016/s2215-0366(21)00243-1 [published Online First: 20211012]
2. Luo H, Li Y, Yang BX, et al. Psychological interventions for personal stigma of patients with schizophrenia: A systematic review and network meta-analysis. *J Psychiatr Res* 2022;148:348–56. doi: 10.1016/j.jpsychires.2022.02.010 [published Online First: 20220216]
3. Nijman SA, Veling W, van der Stouwe ECD, et al. Social Cognition Training for People With a Psychotic Disorder: A Network Meta-analysis. *Schizophr Bull* 2020;46(5):1086–103. doi: 10.1093/schbul/sbaa023
4. Tan CJ, Shufelt T, Behan E, et al. Comparative effectiveness of psychosocial interventions in adults with harmful use of alcohol: a systematic review and network meta-analysis. *Addiction* 2023;118(8):1414–29. doi: 10.1111/add.16187 [published Online First: 20230404]
5. Wen H, Xiang X, Jiang Y, et al. Comparative efficacy of psychosocial interventions for opioid-dependent people receiving methadone maintenance treatment: A network meta-analysis. *Addiction* 2023;118(6):1029–39. doi: 10.1111/add.16167 [published Online First: 20230308]
6. Zhang P, Zhan J, Wang S, et al. Psychological Interventions on Abstinence in Patients with Alcohol Use Disorder: A Systematic Review and Network Meta-Analysis of Randomized Controlled Trials. *Neuropsychiatr Dis Treat* 2022;18:1815–30. doi: 10.2147/ndt.S372300 [published Online First: 20220823]

7. Gao J, Cao J, Guo T, et al. Association between alcoholic interventions and abstinence rates for alcohol use disorders: A meta-analysis. *Medicine (Baltimore)* 2018;97(50):e13566. doi: 10.1097/md.0000000000013566
8. Grant S, Azhar G, Han E, et al. Clinical interventions for adults with comorbid alcohol use and depressive disorders: A systematic review and network meta-analysis. *PLoS Med* 2021;18(10):e1003822. doi: 10.1371/journal.pmed.1003822 [published Online First: 20211008]
9. Cheng HY, McGuinness LA, Elbers RG, et al. Treatment interventions to maintain abstinence from alcohol in primary care: systematic review and network meta-analysis. *Bmj* 2020;371:m3934. doi: 10.1136/bmj.m3934 [published Online First: 20201125]
10. Gutierrez G, Gizzarelli T, Moghimi E, et al. Online cognitive behavioral therapy (eCBT) for the management of depression symptoms in unipolar and bipolar spectrum disorders, a systematic review and network meta-analysis. *J Affect Disord* 2023;341:379–92. doi: 10.1016/j.jad.2023.09.005 [published Online First: 20230906]

Table S2. Characteristics of the included network meta-analyses

Meta-analysis	Population	Intervention	Comparator	Outcome	Included trials (n)
Mc Glanaghy et al., 2021[1]	Adults aged 18-65 with schizophrenia, psychosis, or related disorder (schizophreniform, schizoaffective, delusional disorder), per diagnostic or clinical criteria.	Any psychological intervention delivered individually or in groups.	Treatment as usual (levels 0-3 by care intensity), befriending, supportive counselling, occupational therapy.	Total symptom severity at post-intervention.	94
Miklowitz et al., 2021[2]	Adults and adolescents (age >=12) with bipolar disorder I, II, or unspecified, in any clinical state.	Manualized individual, family, or group psychosocial intervention combined with pharmacotherapy.	Brief psychoeducation (<=3 sessions), supportive therapy, or treatment as usual.	Episode recurrence at 12 months. Depressive and manic symptom severity at 12 months. Study retention.	39
Setkowski et al., 2023[3]	Adults outpatients (age >=18) with (sub)clinical borderline personality disorder, diagnosed by structured interview.	Any stand-alone psychotherapy delivered in an outpatient setting, including specialised generic, and mixed approaches.	Treatment as usual, Generic Treatments, or Community Treatment by Experts.	Borderline personality disorder severity at post-treatment. Suicidal behaviour - combined rate of suicide attempts and deaths. Study drop-out.	43
Solmi et al., 2021[4]	Adult outpatients (age >=18) with acute anorexia nervosa.	Stand-alone outpatient psychological interventions.	Treatment as usual (specialist outpatient standard care).	Body-mass index changes at 52 weeks. Eating disorder psychopathology at 52 weeks. All-cause dropout.	13
Slade et al., 2018[5]	Adults (aged >=18) with bulimia nervosa.	Psychological, pharmacological, or combination therapy licensed in the UK for bulimia nervosa.	Wait list or another active treatment (head-to-head comparisons).	Full remission at end of treatment.	21
De Crescenzo et al., 2018[6]	Adults with cocaine and/or amphetamine addiction actively seeking treatment.	Structured psychosocial intervention, alone or combined.	Treatment as usual or another active psychosocial intervention.	Abstinence at end of treatment assessed by urinalysis. All-cause dropout.	50

Note.

[1]Mc Glanaghy E, Turner D, Davis GA, et al. A network meta-analysis of psychological interventions for schizophrenia and psychosis: Impact on symptoms. Schizophr Res 2021;228:447–59.
[2] Miklowitz DJ, Efthimiou O, Furukawa TA, et al. Adjunctive Psychotherapy for Bipolar Disorder: A Systematic Review and Component Network Meta-analysis. JAMA Psychiatry 2021;78:141–50.
[3] Setkowski K, Palantza C, van Ballegooijen W, et al. Which psychotherapy is most effective and acceptable in the treatment of adults with a (sub)clinical borderline personality disorder? A systematic review and network meta-analysis. Psychol Med 2023;53:3261–80.
[4] Solmi M, Wade TD, Byrne S, et al. Comparative efficacy and acceptability of psychological interventions for the treatment of adult outpatients with anorexia nervosa: a systematic review and network meta-analysis. Lancet Psychiatry 2021;8:215–24.
[5] Slade E, Keeney E, Mavranezouli I, et al. Treatments for bulimia nervosa: a network meta-analysis. Psychol Med 2018;48:2629–36.
[6] De Crescenzo F, Ciabattini M, D'Alò GL, et al. Comparative efficacy and acceptability of psychosocial interventions for individuals with cocaine and amphetamine addiction: A systematic review and network meta-analysis. PLoS Med 2018;15:e1002715

Table S3. Categories of adaptations and their definitions

Domain	Domain code	Subtype	Definition
Content	Mod. Content	Modified components	Addition, removal, or alteration of any specific therapeutic ingredient or component of the original intervention.
Content	Mod. Content	Adapted for population	Content modifications introduced to tailor the intervention to a particular clinical population (e.g., diagnosis, age group, severity) while keeping the overall structure similar.
Delivery	Mod. Delivery	Format	Changes in whether the intervention is delivered in an individual or group format, compared to the original intervention design.
Delivery	Mod. Delivery	Setting	Changes in the physical or virtual setting of delivery, such as moving from in-person to remote/online delivery or the opposite.
Delivery	Mod. Delivery	Mode	Changes in how the intervention is provided, such as shifting from self-help/unguided formats to therapist-guided formats, or vice versa, relative to the original design.
Structure	Mod. Structure	Altered number of sessions	Increase or decrease in the total number of treatment sessions compared with the original intervention design.
Structure	Mod. Structure	Altered frequency of sessions	Changes in how often sessions occur (e.g., weekly vs fortnightly) compared with the original intervention design.
Structure	Mod. Structure	Altered length of sessions	Increase or decrease in the duration of individual sessions compared with the original intervention design.
Structure	Mod. Structure	Altered internal structure of sessions	Reordering or restructuring the sequence of contents within one or more sessions, without necessarily changing the components themselves.
Cultural	Mod. Cultural		Changes introduced to align the intervention with different cultural norms, values, language, religious beliefs, idiomatic expressions, or expectations, while retaining the core function and scope of the treatment.
Unknown	Mod. Unknown		Any mention of modification or adaptation to the original intervention where the nature of the changes is not described or is too vague to classify into other subtypes.

Note. Mod, modification

Supplementary Results

Table S4. Intervention macro-families with total psychological intervention arms, consolidated distinct interventions and distinct interventions with publicly accessible manuals.

Acronym	Macro-family	Total[a]	Distinct[b]	Distinct (%)[c]	Open-access[d]
CBT	Cognitive behavioral therapy	134	89	66%	20
CM	Contingency management	60	22	37%	11
PSED	Psychoeducation	37	26	70%	9
PSYD	Psychodynamic psychotherapy	27	22	81%	2
SUP	Supportive therapy	26	23	88%	5
CR	Cognitive remediation	26	14	54%	4
DBT	Dialectical behavior therapy	26	13	50%	3
CRA	Community reinforcement approach	15	4	27%	2
SCR	Social cognitive remediation	13	8	61%	2
SST	Social skills training	12	7	58%	3
FT	Family therapy	11	9	82%	5
CLIN	Clinical intervention/management	9	6	67%	3
TSF	Twelve-Step facilitation	9	5	55%	3
SHP	Self-help	8	5	62%	
IPT	Interpersonal psychotherapy	6	5	83%	1
ERP	Exposure and response prevention	6	4	67%	
MCT	Metacognitive training	6	3	50%	3
DC	Drug counselling	5	4	80%	
SCH	Schema therapy	5	4	80%	1
IPSRT	Interpersonal and social rhythm therapy	5	3	60%	
SSCM	Specialistic supportive clinical management	5	1	20%	
CAT	Cognitive analytic therapy	4	3	75%	1
REL	Relaxation	3	3	100%	1
MANTRA	Maudsley model of anorexia nervosa treatment for adults	3	1	33%	1
MF-PSED	Mindfulness-based psychoeducation	3	1	33%	1
BFT	Behavioral family therapy	2	2	100%	
PS	Problem Solving	2	1	50%	1
SCM	Structured clinical management	2	1	50%	
COPE	Coping skills	1	1	100%	1
EMDR	Eye movement desensitization and reprocessing	1	1	100%	
FR	Functional remediation	1	1	100%	
FUN	Functional analysis	1	1	100%	1
GSD	Guided self-determination	1	1	100%	
HHS	Holistic health sessions	1	1	100%	1
HIT	Hallucinations focused integrative treatment	1	1	100%	
ILLMNG	Illness management	1	1	100%	
LEAP	Compulsive exercise activity therapy	1	1	100%	1
MBT	Mind-Body Therapy	1	1	100%	
MFT	Multifamily treatment	1	1	100%	
NSDG	Non-specific discussion group	1	1	100%	
NSIT	Non-specialistic Intervention	1	1	100%	
P-OT	Psychological based occupational therapy	1	1	100%	
PP	Positive psychotherapy	1	1	100%	1
PSYA	Psychoanalysis	1	1	100%	

Acronym	Macro-family	Total[a]	Distinct[b]	Distinct (%)[c]	Open-access[d]
PSYSOC	Psychosocial intervention	1	1	100%	

Note. The macrofamilies are listed in decreasing order starting with the ones with those including the highest number of total arms. Interventions assigned to multiple macro-families were counted in each relevant macro-family.
[a] Absolute frequency of active psychological intervention arms.
[b] Distinct interventions
[c] Proportion out of total number of active psychological intervention arms in the macro-family.
[d] Distinct interventions with publicly accessible manuals

Table S5. Total psychological intervention arms with macro-family and adaptations – Anorexia Nervosa

Trial reference (author, year)	Original Intervention Name	Distinct Intervention Name	Macro-family intervention 1	Macro-family intervention 2	Manuals references	Type of Adaptation (content, duration/sessions, cultural, unknown)
Ball, 2004	Behavioral family therapy	BFT (Robin & Foster, 1989, Adapted Ball et al., 2004)	BFT		Robin AL, & Foster SL (1989). Negotiating parent-adolescent conflict: A behavioral-family systems approach. New York: Guilford.	Content
Ball, 2004	Cognitive-behavioral therapy	CBT (Garner & Bemis, 1982, Adapted Ball et al., 2004)	CBT		Garner DH, & Bemis KM (1982). Cognitive behavior therapy for anorexia nervosa. In Garner DM, & Garfinkel PE (Eds.), Handbook of psychotherapy for anorexia nervosa and bulimia. New York: Guilford Press.<br>Young, J. (1994). Cognitive therapy for personality disorders: A schema-focused approach (2nd ed.). Sarasota, FL: Professional Resource Press.	Content
Byrne, 2017	Cognitive-behavioral therapy-E-f/b	CBT-E-f/b (Fairburn, 2008)	CBT		Fairburn CG, Cooper Z, Shafran R, Bohn K, Hawker DM, Murphy R, Straebler S (2008) Enhanced cognitive behavior therapy for eating disorders: the core protocol, in Cognitive Behavior Therapy and Eating Disorders. Edited by Fairburn CG. New York, Guilford.<br>Fairburn CG, Cooper Z, Shafran R, Bohn K, Hawker DM: Clinical perfectionism, core low self-esteem, and interpersonal problems, in Cognitive Behavior Therapy and Eating Disorders. Edited by Fairburn CG. New York, Guilford, 2008	
Byrne, 2017	MANTRA	MANTRA (Schmidt, 2012, 2014)	MANTRA		Schmidt U, Wade TD, Treasure J (2014). The Maudsley model of anorexia nervosa treatment for adults (MANTRA): development, key features, and preliminary evidence.	
Byrne, 2017	SSCM	SSCM (McIntosh, 1997, unpublished)	SSCM		McIntosh VV, Jordan J, Joyce PR, McKenzie JM, Luty SE, Carter FA, Bulik CM. (1997) [unpublished] SPECIALIST SUPPORTIVE CARE THERAPIST MANUAL	
Crisp, 1991	Family/individual psychodynamic oriented therapy	Family/individual psychodynamic oriented therapy	PSYD		Not manualized	
Crisp, 1991	Group (patients, family) therapy	Group (patients, family) therapy	PSYD		Not manualized	
Crisp, 1991	Individual inpatient therapy (eclectic)	Individual inpatient therapy (eclectic) (Crisp, 1991)	PSYD		Crisp AH (1980). Anorexia Nervosa: Let me be. London: Academic Press	
Dare, 2001	Cognitive analytic therapy	CAT (Ryle, 1990)	CAT		Ryle A (1990). Cognitive-Analytic Therapy: Active Participation in Change. London: John Wiley & Sons	
Dare, 2001	Family therapy	Family therapy (Dare & Eisler, 1995)	FT		Dare C & Eisler I (1995) Family therapy. In Szmukler G, Dare C, Treasure J (eds.) Eating Disorders: Handbook of Theory, Treatment and Research. Chichester: John Wiley & Sons	Delivery

Trial reference (author, year)	Original Intervention Name	Distinct Intervention Name	Macro-family intervention 1	Macro-family intervention 2	Manuals references	Type of Adaptation (content, duration/sessions, cultural, unknown)
Dare, 2001	Focal psychoanalytic psychotherapy	Focal psychoanalytic psychotherapy (Dare, 1995)	PSYA		Dare C (1995). Psychoanalytic psychotherapy (of eating disorders). In Gabbard GO. (ed) Treatment of Psychiatric Disorders, Washington DC: American Psychiatric Press.<br>Dare C & Crowther C (1995) Living dangerously: psychoanalytic psychotherapy of anorexia nervosa. In Szmukler G, Dare C, Treasure J (eds.) Eating Disorders: Handbook of Theory, Treatment and Research. Chichester: John Wiley & Sons	
Hall, 1987	Family & psychodynamic	Family & psychodynamic (Hall & Crisp, 1987, no manual)	FT	PSYD	Not manualized	
Hall, 1987	Diet, mood, behavior group	Diet, mood, behavior group (Hall & Crisp, 1987, no manual)	NSIT		Not manualized	
Hay, 2018	Cognitive-behavioral therapy-AN	CBT-AN (Pike, 2003, unpublished)	CBT		Pike KM, Carter J, Olmsted M (2003) [unpublished]. Cognitive behavioral therapy manual for Anorexia Nervosa	
Hay, 2018	Cognitive-behavioral therapy-AN + LEAP	CBT-AN (Pike, 2003, unpublished) + LEAP (Taranis et al., 2011)	CBT	LEAP	Pike KM, Carter J, Olmsted M (2003). Cognitive behavioral therapy manual for Anorexia Nervosa.<br>Taranis L, Touyz S, La Puma M, Meyer C. (2011) Loughborough Eating-disorders Activity Programme “LEAP”	
Lock, 2013	Cognitive-behavioral therapy	CBT (Halmi, 2005)	CBT		Halmi CA, Agras WS, Crow SJ, Mitchell J, Wilson GT, Bryson S, Kraemer H (2005). Predictors of treatment acceptance and completion in anorexia nervosa: Implications for future study designs. Arch Gen Psychiatry 62.	Unknown
Lock, 2013	Cognitive remediation therapy	CR (Tchanturia & Lock, 2011)	CR		Tchanturia K, Lock J (2011) Cognitive remediation therapy (CRT) for eating disorders: Development, refinement, and future directions. Curr Top Behav Neurosci; 6:269–297.	
McIntosh, 2005	Cognitive-behavioral therapy	CBT (Carter 1997, Adapted from Bulik 1993 unpublished, and Garner 1997)	CBT		Carter FA, McIntosh VV, Jordan J, Joyce PR, McKenzie JM, Bulik CM. (1997) Cognitive Behavioural Therapy Therapist Manua.<br>Bulik C, Sullivan PF, Carter FA, Joyce PR (1993) Cognitive Therapy Therapist Manual for the treatment of Bulimia Nervosa.<br>Garner DM, Vitousek KM, Pike KM (1997) Cognitive-behavioral therapy for anorexia nervosa. In Garner DM, & Garfinkel PE (Eds.), Handbook of psychotherapy for anorexia nervosa and bulimia. New York: Guilford Pressl	Unknown
McIntosh, 2005	Interpersonal psychotherapy	IPT (McKenzie, 2005, Adapted from Klerman, 1984 and Fairburn, 1993)	IPT		McKenzie JM, McIntosh VV, Luty SE, Jordan J, Joyce PR, Carter FA, Bulik CM. (1997) Interpersonal Psychotherapy Therapist manual.<br>Klerman GL, Weissman MM, Rounsaville BJ, Chevron ES (1984) Inter personal Psychotherapy of Depression. New York: Basic Books.<br>Fairburn CG (1993) Interpersonal psychotherapy for bulimia nervosa. In	

Trial reference (author, year)	Original Intervention Name	Distinct Intervention Name	Macro-family intervention 1	Macro-family intervention 2	Manuals references	Type of Adaptation (content, duration/sessions, cultural, unknown)
					Klerman GL, Weissman MM (Eds.) New Applications of Interpersonal Psychotherapy. Washington, DC: American Psychiatric Press	
McIntosh, 2005	SSCM	SSCM (McIntosh, 1997, unpublished)	SSCM		McIntosh VV, Jordan J, Joyce PR, McKenzie JM, Luty SE, Carter FA, Bulik CM. (1997) [unpublished] Specialist Supportive Care Therapist Manual	
Schmidt, 2012	MANTRA	MANTRA (Schmidt, 2012, 2014)	MANTRA		Schmidt U, Oldershaw A, Jichi F, Sternheim L, Startup H, McIntosh V, Jordan J, Tchanturia K, Wolff G, Rooney M, Landau S, Treasure J (2012) Out-patient psychological therapies for adults with anorexia nervosa: randomised controlled trial. BJPsych 201, 392-399.	
Schmidt, 2012	SSCM	SSCM (McIntosh, 1997, unpublished)	SSCM		McIntosh VV, Jordan J, Joyce PR, McKenzie JM, Luty SE, Carter FA, Bulik CM. (1997) [unpublished] Specialist Supportive Care Therapist Manual	
Schmidt, 2015	MANTRA	MANTRA (Schmidt, 2012, 2014)	MANTRA		Schmidt U, Oldershaw A, Jichi F, Sternheim L, Startup H, McIntosh V, Jordan J, Tchanturia K, Wolff G, Rooney M, Landau S, Treasure J (2012) Out-patient psychological therapies for adults with anorexia nervosa: randomised controlled trial. BJPsych 201, 392-399.	
Schmidt, 2015	SSCM	SSCM (McIntosh, 1997, unpublished)	SSCM		McIntosh VV, Jordan J, Joyce PR, McKenzie JM, Luty SE, Carter FA, Bulik CM. (1997) [unpublished] Specialist Supportive Care Therapist Manual	
Touyz, 2013	Cognitive-behavioral therapy-AN	CBT-AN (Pike, 2003, unpublished)	CBT		Pike KM, Carter J, Olmsted M (2003) [unpublished]. Cognitive behavioral therapy manual for Anorexia Nervosa	Content
Touyz, 2013	SSCM	SSCM (McIntosh, 1997, unpublished)	SSCM		McIntosh VV, Jordan J, Joyce PR, McKenzie JM, Luty SE, Carter FA, Bulik CM. (1997) [unpublished] SPECIALIST SUPPORTIVE CARE THERAPIST MANUAL	Content
Treasure, 1995	Cognitive analytic therapy	CAT (Ryle, 1990)	CAT		Ryle A (1990). Cognitive-Analytic Therapy: Active Participation in Change. London: John Wiley & Sons	
Zipfel, 2014	Cognitive-behavioral therapy-E-b	CBT-E-f/b (Fairburn, 2008)	CBT		Fairburn CG, Cooper Z, Shafran R, Bohn K, Hawker DM, Murphy R, Straebler S (2008) Enhanced cognitive behavior therapy for eating disorders: the core protocol, in Cognitive Behavior Therapy and Eating Disorders. Edited by Fairburn CG. New York, Guilford. Fairburn CG, Cooper Z, Shafran R, Bohn K, Hawker DM: Clinical perfectionism, core low self-esteem, and interpersonal problems, in Cognitive Behavior Therapy and Eating Disorders. Edited by Fairburn CG. New York, Guilford, 2008.	

Trial reference (author, year)	Original Intervention Name	Distinct Intervention Name	Macro-family intervention 1	Macro-family intervention 2	Manuals references	Type of Adaptation (content, duration/sessions, cultural, unknown)
Zipfel, 2014	Focal psychodynamic therapy	Focal psychodynamic therapy (Schauenburg, 2009)	PSYD		Schauenburg H, Friederich H-C, Wild B, Zipfel S, Herzog W (2009) Focal psychodynamic psychotherapy of anorexia nervosa: a treatment manual. Psychotherapeut; 54: 270–80	

List of acronyms for interventions and families: BFT: Behavioral family therapy. CAT: Cognitive analytic therapy. CBT: Cognitive-behavioral therapy. CBT-AN: Cognitive-behavioral therapy for Anorexia Nervosa. CBT-E-b: Enhanced Cognitive-behavioral therapy – broad. CBT-E-b/f: Enhanced Cognitive-behavioral therapy – broad/focused. CR: Cognitive remediation therapy. FT: Family Therapy. IPT: Interpersonal Psychotherapy. LEAP: Compulsive Exercise Activity Therapy. MANTRA: Maudsley anorexia nervosa treatment for adults. NSIT: Non-specialistic intervention. PSYA: Psychoanalytic Psychotherapy. PSYD: Psychodynamic Psychotherapy. SSCM: Specialist Supportive Clinical Management.

Table S6. Total psychological intervention arms with macro-family and adaptations – Bipolar disorder

Trial reference (author, year)	Original Intervention Name	Distinct Intervention Name	Macro-family intervention 1	Macro-family intervention 2	Manuals references	Type of Adaptation (content, duration/sessions, cultural, unknown)
Ball, 2006	Cognitive therapy	CT (Ball, 2006, unpublished)	CBT		Ball JR, Mitchell PB, Corry JC, Skillecorn A, Smith M, Malhi GS (2006) [unpublished]. Cognitive Therapy: Therapist Manual.	
Bordbar, 2009	Family focused psychoeducational program	PSED-FC (Bordbar, 2009, no manual)	PSED		Not manualized	
Cardoso, 2015	Psychoeducation	PSED (Adapted from Colom & Vieta, 2004)	PSED		Colom F, & Vieta E (2004). Improving the outcome of bipolar disorder through non pharmacological strategies: The role of psychoeducation. Rev Bras Psiquiatr. 26:(suppl 3):47–50.	Structure
Castle, 2010	Group based psychosocial intervention	GPI (Castle, 2007)	PSED	CBT	Castle D, Berk M, Berk L, Lauder S, Chamberlain J, Gilbert M (2007). Pilot of group intervention for bipolar disorder. int J Psychiatry Clin Pract; 11: 279–84.	
Cochran, 1984	Cognitive-behavioral therapy-based compliance intervention	CBT-C (Adapted from Beck, 1979)	CBT		Beck AX, Rush AJ, Shaw BE, & Emery G (1979). Cognitive therapy of depression. New York: Guilford Press.	Content, Structure
Colom, 2009	Group psychoeducation	PSED (Colom & Vieta, 2006)	PSED		Colom F, & Vieta E (2006). Psychoeducation Manual for Bipolar Disorder. Cambridge, New York.	
Costa, 2011	Cognitive-behavioral group therapy	CBGT (Adapted from Basco & Rush, 1996)	CBT		Basco MR, & Rush AJ (1996). Cognitive-behavioral therapy for bipolar disorder. New York: Guilford Press.	Structure, Delivery
D'Souza, 2010	Systematic illness management skills enhancement programme-bipolar disorder	SIMSEP-BD (D'Souza & Rich, 2002)	PSED		D'Souza R, & Rich D (2002). A case-control study in the use of 'Illness Management Skills Enhancement Programme' for treatment adherence in patients with a bipolar disorder. Bipolar Disord. 4, 121.	
deBarros, 2013	Group psychoeducation	PSED (Adapted from Colom & Vieta, 2006)	PSED		Colom F, & Vieta E (2006) Psychoeducation manual for bipolar disorder, 1st edn., New York, Cambridge University Press.	Structure
deBarros, 2013	Relaxation group	REL (Adapted from Jacobson, 1955)	REL		Jacobson E (1995). Neuromuscular controls in man: methods of self direction in health and in disease. Am J Psychol;68:549–561.	Content, Structure
Frank, 2005	Interpersonal and Social Rhythm therapy acute +	IPSRT (Frank, 2005)	IPSRT		Frank E (2005). Treating Bipolar Disorder: A Clinician's Guide to interpersonal and Social Rhythm Therapy. New York, NY: Guilford Press.	

Trial reference (author, year)	Original Intervention Name	Distinct Intervention Name	Macro-family intervention 1	Macro-family intervention 2	Manuals references	Type of Adaptation (content, duration/sessions, cultural, unknown)
	maintenance + PHA					
Frank, 2005	Intensive clinical management acute + maintenance + PHA	ICM (Adapted from Fawcett, 1987)	CLIN		Fawcett J, Epstein P, Fiester SJ, Elkin I, Autry JH (1987). Clinical management— imipramine/placebo administration manual: NIMH Treatment of Depression Collaborative Research Program. Psychopharmacol Bull;23:309-324.	Content, Structure
Frank, 2005	Interpersonal and Social Rhythm therapy acute + Intensive clinical management maintenance + PHA	IPSRT (Frank, 2005) + ICM (Adapted from Fawcett, 1987)	IPSRT	CLIN	Frank E (2005). Treating Bipolar Disorder: A Clinician's Guide to interpersonal and Social Rhythm Therapy. New York, NY: Guilford Press.<br>Fawcett J, Epstein P, Fiester SJ, Elkin I, Autry JH (1987). Clinical management— imipramine/placebo administration manual: NIMH Treatment of Depression Collaborative Research Program.	Content, Structure
Frank, 2005	Intensive clinical management acute + Interpersonal and Social Rhythm therapy maintenance + PHA	ICM (Adapted from Fawcett, 1987) + IPSRT (Frank, 2005)	CLIN	IPSRT	Fawcett J, Epstein P, Fiester SJ, Elkin I, Autry JH (1987). Clinical management— imipramine/placebo administration manual: NIMH Treatment of Depression Collaborative Research Program.<br>Frank E (2005). Treating Bipolar Disorder: A Clinician's Guide to interpersonal and Social Rhythm Therapy. New York, NY: Guilford Press. Psychopharmacol Bull;23:309-324.	Content, Structure
Goldstein, 2015	Dialectic behaviour therapy	DBT (Goldstein, 2007, Adapted from Miller, 2006)	DBT		Goldstein TR, Axelson DA, Birmaher B, Brent DA (2007). Dialectical behavior therapy for adolescents with bipolar disorder: A one-year open trial. J Am Acad Child Adolesc Psychiatry 46:820–830.<br>Miller AL, Rathus JH, Linehan MM (2006). Dialectical Behavior Therapy with Suicidal Adolescents. New York: Guilford Press.	Content, Structure
Gomes, 2011	Cognitive-behavioral group therapy	CBGT (Gomes, 2010)	CBT		Gomes, BC (2010). Estudo controlado de terapia cognitivo comportamental em grupo no tratamento de pacientes com transtorno bipolar (Dissertação (Mestrado). Universidade de São Paulo, São Paulo. [Doctoral thesis]	
Gonzáles Isasi, 2014	Psychoeducational and CBT based group	CT/CBT (Adapted from Lam, 1999)	CBT		Lam DH, Jones S, Hayward P, Bright J (1999). Cognitive therapy for bipolar disorder: a therapist's guide to concepts. Methods and Practice. New York: John Wiley & Sons.	Structure
Harvey, 2015	Cognitive-behaviour therapy for insomnia-bipolar disorder	CBTI-BP (Harvey, 2015)	CBT		Harvey AG, Soehner AM, Kaplan KA, Hein K, Lee J, Kanady J, Li D, Rabe-Hesketh S, Ketter TA, Neylan TC, Buysse DJ (2015). Treating insomnia improves mood state, sleep, and functioning in bipolar disorder: a pilot randomized controlled trial. J Consult Clin Psychol.;83(3):564-77.	

Trial reference (author, year)	Original Intervention Name	Distinct Intervention Name	Macro-family intervention 1	Macro-family intervention 2	Manuals references	Type of Adaptation (content, duration/sessions, cultural, unknown)
					doi: 10.1037/a0038655. Epub 2015 Jan 26. PMID: 25622197; PMCID: PMC4446240. [Supplementary]	
Harvey, 2015	Psychoeducation	PSED (Harvey, 2015, unpublished)	PSED		Harvey AG (2015) [unpublished]. Psychoeducation: Therapist Manual.	
Hautzinger, 2024	Specific, emotional-cognitive group psychotherapy	SEKT (Hautzinger, 2024)	CBT		Hautzinger M, on behalf of A2 BipoLife Consortium (2024). Adjuvant Psychotherapies to Prevent Relapse in Bipolar Disorder: A Randomized Clinical Trial. JAMA Psychiatry; e241310. [Supplementary]	
Hautzinger, 2024	Emotionally demanding, supportive, psychoeducational psychotherapy	FEST (Hautzinger, 2024)	SUP	PSED	Hautzinger M, on behalf of A2 BipoLife Consortium (2024). Adjuvant Psychotherapies to Prevent Relapse in Bipolar Disorder: A Randomized Clinical Trial. JAMA Psychiatry; e241310. [Supplementary]	
Inder, 2015	Interpersonal and Social Rhythm therapy + PHA	IPSRT (Frank, 2001, unpublished)	IPSRT		Frank E, Kupfer DJ, Cornes C, Carter SB, Frankel D, Hlastala SA (2001) [unpublished]. Interpersonal and Social Rhythm Therapy (IPSRT). A manual for the adaptation of interpersonal psychotherapy to the treatment of bipolar disorder. Presented at the Fourth International Conference on Bipolar Disorder, June 14–16, Pittsburgh, PA.	
Inder, 2015	SSCM + PHA	SSCM (Crowe, 2003, unpublished)	CLIN	SUP	Crowe MT, Moor S, Joyce PR, Luty SE, Janet DC (2003) [unpublished]. Specialist Supportive Care Therapist Manual.	
Javadpour, 2013	Psychoeducation	PSED (Adapted from Colom & Vieta, 2006)	PSED		Colom F, & Vieta E (2006). Psychoeducation Manual for Bipolar Disorder. Cambridge, New York.	Structure
Jones, 2015	Recovery focused cognitive-behavioral therapy	CBT-RF (Jones, 2012)	CBT		Jones S, Mulligan LD, Law H, Dunn G, Welford M, Smith G, Morrison AP (2012). A randomised controlled trial of recovery focused CBT for individuals with early bipolar disorder. BMC psychiatry, 12, 1-8.	
Lam, 2000	Individual cognitive therapy	CT/CBT (Lam, 2000)	CBT		Lam DH, Bright J, Jones S, Hayward P, Schuck N, Chisholm D, Sham P (2000). Cognitive therapy for bipolar illness: Pilot study of relapse prevention. Cognitive Therapy and Research;24:503-520. [Supplementary]	
Lam, 2005	Individual cognitive therapy	CT/CBT (Lam, 1999)	CBT		Lam DH, Jones S, Hayward P, Bright J (1999). Cognitive Therapy for Bipolar Disorder: A Therapist's Guide to Concepts, Methods and Practice. New York, John Wiley & Sons.	

Trial reference (author, year)	Original Intervention Name	Distinct Intervention Name	Macro-family intervention 1	Macro-family intervention 2	Manuals references	Type of Adaptation (content, duration/sessions, cultural, unknown)
Lobban, 2010	Enhanced relapse prevention	ERP (Adapted from Morriss, 2004)	PSED		Morriss R (2004). The early warning symptom intervention for clients with bipolar affective disorder. Advances in Psychiatric Treatment; 10:18-26.	Content, Structure
Meyer, 2012	Cognitive-behavioral therapy	CBT (Meyer & Hautzinger, 2004)	CBT		Meyer TD, & Hautzinger M (2004). Manic Depressive Disorders – Cognitive Behaviour Therapy for Relapse Prevention [in German]. Beltz: Weinheim.	
Meyer, 2012	Supportive therapy	SUP (Meyer, 2012, unpublished)	SUP		Meyer TD (2012) [unpublished]. Supportive Therapy: Therapist Manual.	
Miklowitz, 2003	Family focused therapy	FFT (Miklowitz & Goldstein, 1997)	FT		Miklowitz DJ, & Goldstein MJ (1997). Bipolar Disorder: A Family-Focused Treatment Approach. New York, NY Guilford Press.	
Miklowitz, 2003	Crisis Management	CR-M (George, 2002)	CLIN		George EL,Taylor DO, Miklowitz DJ (2002). A case management protocol for patients with bipolar disease. Bipolar Update; 112-13.	
Miklowitz, 2007	Family focused therapy	FFT (Miklowitz & Goldstein, 1997)	FT		Miklowitz DJ, & Goldstein MJ (1997). Bipolar Disorder: A Family-Focused Treatment Approach. New York, NY Guilford Press.	
Miklowitz, 2007	Cognitive-behavioral therapy	CBT (Otto & Reilly-Harrington, 2002)	CBT		Otto MW, & Reilly-Harrington NA (2002). Cognitive-behavior therapy for the management of bipolar disorder. In: Hofmann, SG.; Tompson, MC., editors. Handbook of Psychosocial Treatments for Severe Mental Disorders. New York, NY: Guilford Press, 116-130.	
Miklowitz, 2007	Interpersonal and Social Rhythm therapy	IPSRT (Frank, 2005)	IPSRT		Frank E (2005). Treating Bipolar Disorder: A Clinician's Guide to Interpersonal and Social Rhythm Therapy. New York, NY: Guilford Press.	
Miklowitz, 2007	Collaborative care - Psychoeducation	PSED-CC (Miklowitz, 2007, unpublished)	PSED		Miklowitz (2007) [unpublished]. Collaborative Care: Therapist Manual.	
Miklowitz, 2008	Family focused therapy for adolescents	FFT-A (Miklowitz, 2004; Miklowitz, 2006)	FT		Miklowitz DJ, George EL, Axelson DA, Kim EY, Birmaher B, Schneck C, Beresford C, Craighead WE, Brent DA (2004). Family-focused treatment for adolescents with bipolar disorder. J Affect Disord;82(Suppl 1):113–128.<br>Miklowitz DJ, Biuckians A, Richards JA (2006). Early-onset bipolar disorder: a family treatment perspective. Dev Psychopathology;18(4):1247–1265.	
Miklowitz, 2008	Enhanced care - psychoeducation	PSED-EC (Miklowitz 2008, unpublished)	PSED		Miklowitz (2008) [unpublished]. Enhanced Care: Therapist Manual.	

Trial reference (author, year)	Original Intervention Name	Distinct Intervention Name	Macro-family intervention 1	Macro-family intervention 2	Manuals references	Type of Adaptation (content, duration/sessions, cultural, unknown)
Miklowitz, 2014	Family focused therapy for adolescents	FFT-A (Miklowitz, 2014, unpublished)	FT		Miklowitz DJ (2014) [unpublished]. Family-Focused Therapy (FFT) for Youth with Mood and Psychotic Disorders: Clinicians treatment Manual.	
Miklowitz, 2014	Enhanced care - psychoeducation	PSED-EC (Miklowitz, 2012)	PSED		Miklowitz DJ, George EL, Taylor DO (2012). Enhanced care manual: Clinician's manual for Brief Psychoeducational Treatment of Children and Adolescents at Risk of Bipolar Disorder.	
Morriss, 2016	Group psychoeducation	PSED (Morriss, 2011)	PSED		Morriss RK, Lobban F, Jones S, Riste L, Peter S, Roberts C, Davies L, Mayes D (2011). Pragmatic randomised controlled trial of group psychoeducation versus group support in the maintenance of bipolar disorder. BMC Psychiatry; 11: 214.	
Morriss, 2016	Group peer support	GPS (Morriss, 2016, unpublished)	SUP		Morriss RK (2016) [unpublished] Group Peer Support: Therapist Manual.	
Nagy, 2015	Behavioral family therapy	BFT (Adapted from Mueser & Glynn, 1999)	FT		Mueser K, & Glynn S (1999). Behavioral family therapy for psychiatric disorders. Oakland: New Harbinger Publications.	Cultural
Nagy, 2015	Supportive psychotherapy	PSED-FC (Nagy, 2015, no manual)	PSED		Not manualized	
Parikh, 2012	Cognitive-behavioral therapy	CT/CBT (Adapted from Lam, 1999)	CBT		Lam DH, Jones S, Hayward P, Bright J (1999). Cognitive therapy for bipolar disorder: a therapist's guide to concepts. Methods and Practice. New York: John Wiley & Sons.	Structure
Parikh, 2012	Group psychoeducation	PSED-LFG (Bauer & McBride, 2003)	PSED		Bauer MS, & McBride L (2003). Structured Group Psychotherapy for Bipolar Disorder: The Life Goals Program. 2nd ed. New York, NY: Springer Publishing Company.	
Perich, 2013	Mindfulness-based cognitive therapy	MBCT (Perich, 2013, unpublished)	CBT		Perich T (2013) [unpublished]. Mindfulness-Based Cognitive Therapy - MBCT: Therapist Manual.	
Perlick, 2018	Family focused therapy - health promoting intervention	FFT-HIP (Perlick, 2010)	FT		Perlick DA, Miklowitz DJ, Lopez N, Chou J, Kalvin C, Adzhiashvili V, Aronson A (2010). Family-focused treatment for caregivers of patients with bipolar disorder. Bipolar Disord.2010;12:627-637.	
Perry, 1999	Psychoeducation	PSED (Perry, 1995)	PSED		Perry A, Tarrier N, Morriss R (1995). Identification of prodromal signs and symptoms and early intervention in manic depressive psychosis patients: a case example. Behav Cognit Psychother;23:399409	
Rea, 2003	Family focused therapy	FFT (Miklowitz & Goldstein, 1997)	FT		Miklowitz DJ, & Goldstein MJ (1997). Bipolar disorder: A family focused treatment approach. New York: Guilford Press.	

**Trial reference (author, year)**	**Original Intervention Name**	**Distinct Intervention Name**	**Macro-family intervention 1**	**Macro-family intervention 2**	**Manuals references**	**Type of Adaptation (content, duration/sessions, cultural, unknown)**
Rea, 2003	Individually focused patient treatment	IFPT (Rea, 2003, no manual)	PSED	CBT	Not manualized	
Reinares, 2008	Caregiver group psychoeducation	PSED-FC (Reinares, 2008, unpublished)	PSED		Reinares M (2008) [unpublished]. Manual de psicoeducación para el trabajo con familiares de pacientes con trastorno bipolar.	
Sajatovic, 2009	Group psychoeducation (Life Goals Program)	PSED-LFG (Bauer & McBride, 2003)	PSED		Bauer MS, & McBride L (2003). Structured Group Psychotherapy for Bipolar Disorder: The Life Goals Program. 2nd ed. New York, NY: Springer Publishing Company.	
Scott, 2001	Cognitive therapy	CT/CBT (Scott, 2001)	CBT		Scott J, Garland A, Moorhead S (2001). A pilot study of cognitive therapy in bipolar disorders. Psychol Med.; 31:459-467.	
Scott, 2006	Cognitive-behavioral therapy	CT/CBT (Adapted from Scott, 2002)	CBT		Scott J (2002). Overcoming Mood Swings. London: Constable Robinson.	Content, Structure, Delivery
Torrent, 2013	Functional remediation	FR (Torrent, 2013, unpublished)	FR		Torrent C (2013) [unpublished]. Functional Remediation for Bipolar Disorder: Therapist Manual.	
Torrent, 2013	Psychoeducation	PSED (Colom & Vieta, 2006, Adapted by Torrent, 2013, unpublished)	PSED		Colom F, & Vieta E (2006). Psychoeducation Manual for Bipolar Disorder. Cambridge, UK, Cambridge University Press.<br>Torrent C (2013) [unpublished]. Psychoeducation for Bipolar Disorder: Therapist Manual.	
van der Voort, 2015	Collaborative care	CC (van der Voort, 2015)	CLIN		Van der Voort TYG, van Meijel B, Goossens PJJ, Hoogendoorn AW, Draisma S, Beekman A, Kupka RW (2015). Collaborative care for patients with bipolar disorder: randomised controlled trial. Br J Psychiatry; 206(5):393-400	
Van Dijk, 2013	DBT based Psychoeducational Group	DBT-SK (Adapted from Linehan, 1993b)	DBT	PSED	Linehan MM (1993). Skills Training Manual for Treating Borderline Personality Disorder. Guilford Press, NewYork, NY.	Content, Structure

List of acronyms for interventions and families: BFT: Behavioral Family therapy. CBGT: Cognitive Behavioral Group therapy. CBT: Cognitive-behavioral therapy. CBT-C: Cognitive-Behaviour therapy – Compliance. CBT-RF: Recovery focused-Cognitive-Behaviour therapy. CBTI-BP: Cognitive-Behaviour therapy for insomnia-Bipolar Disorder. CC: Collaborative Care. CLIN: Clinical Management. CR-M: Crisis Management. CT: Cognitive therapy. CT/CBT: Cognitive therapy/Cognitive-Behaviour therapy. DBG: DBT based Psychoeducational Group. DBT: Dialectic Behaviour therapy. DBT-SK: Dialectic Behaviour therapy - Skills Training. ERP: Enhanced Relapse Prevention. FEST: Emotionally demanding, Supportive, Psychoeducational psychotherapy. FR: Functional Remediation. FT: Family therapy. FFT: Family Focused therapy. FFT-A: Family Focused therapy for Adolescents. FFT-HIP: Family Focused therapy - Health Promoting Intervention. GPI: Group Psychological Intervention. GPS: Group Peer Support. ICM: Intensive clinical management. IFPT: Individually Focused Patient Treatment. IPSRT: Interpersonal and Social Rhythm therapy. MBCT: Mindfulness-Based Cognitive therapy. PSED: Psychoeducation. PSED-CC: Collaborative Care-Psychoeducation. PSED-EC: Enhanced Care-Psychoeducation. PSED-FC: Family/Caregiver Psychoeducation. PSED-LGP: Psychoeducation - Life Goal Program. REL: Relaxation. SEKT: Specific, Emotional-cognitive group psychotherapy. SIMSEP-BD: Systematic Illness Management Skills Enhancement Programme-Bipolar Disorder. SSCM: Specialist Supportive Care Management. SUP: Supportive therapy.

Table S7. Total psychological intervention arms with macro-family and adaptations – Borderline personality disorder

Trial reference (author, year)	Original Intervention Name	Distinct Intervention Name	Macro-family intervention 1	Macro-family intervention 2	Family intervention 3	Manuals references	Manuals references	Type of Adaptation (content, duration/sessions, cultural, unknown)
Amianto, 2011	Sequential brief adlerian psychodynamic psychotherapy plus supervised team management	AdP + STM (not manualized)	PSYD				Not manualized	
Andreasson, 2016	Dialectical behavior therapy	DBT (adapted from Rathus & Miller, 1995, unpublished, described in Rathus & Miller, 2002)	DBT				Miller AL, Rathus JH, Landsman M, & Linehan MM (1995) DBT skills training manual for suicidal adolescents. Unpublished manual. Montefiore Medical Center/Albert Einstein College of Medicine, Bronx, NY.	Content, structure
Andreasson, 2016	Collaborative assessment and management of suicidality treatment	CAMS (Jobes, 2004; Jobes, 2006)	SUP				Jobes DA, Drozd JF (2004) The CAMS approach to working with suicidal patients. J Contemp Psychother (1):73–85. Jobes DA (2006) Managing Suicidal Risk: A	

Trial reference (author, year)	Original Intervention Name	Distinct Intervention Name	Macro-family intervention 1	Macro-family intervention 2	Family intervention 3	Manuals references	Manuals references	Type of Adaptation (content, duration/sessions, cultural, unknown)
							Collaborative Approach. New York, London: Guilford Press	
Andreoli, 2016	Abandonment psychotherapy delivered by nurses	AP (Andreoli, 2016, unpublished)	PSYD	CBT			Andreoli (2016) [Unpublished] La psychothérapie psychanalytique du deuil traumatique de l'abandon	Delivey
Andreoli, 2016	Abandonment psychotherapy delivered by certified psychotherapists	AP (Andreoli, 2016, unpublished)	PSYD	CBT			Andreoli (2016) [Unpublished] La psychothérapie psychanalytique du deuil traumatique de l'abandon	Delivery
Bateman, 2009	Mentalization based treatment	MBT-O (Bateman & Fonagy 2004, 2006 described in Bateman, 2010; Bateman & Fonagy 2016)	PSYD				Bateman AW, Fonagy P (2004) Psychotherapy for Mentalization-Based Treatment. Oxford, Oxford University Press.<br>Bateman AW, Fonagy P (2006) 'The structure of mentalization-based treatment', Mentalization-based Treatment for Borderline Personality Disorder: A Practical Guide, International Perspectives in Philosophy & Psychiatry (Oxford; online edn, Oxford Academic, 1 Feb. 2013)	
Bateman, 2009	Structured clinical management	SCM (Bateman & Fonagy, 2009, unpublished, described in Bateman & Krawitz, 2013)	SCM				Bateman & Fonagy (2009) [unpublished] "Structured Clinical Management of Borderline Personality Disorder: Best Clinical Practice"<br>Bateman AW, Krawitz R (2013) Borderline Personality Disorder: An Evidence-Based Guide for Generalist Mental Health Professionals. Oxford: Oxford University Press	
Bellino, 2010	Interpersonal psychotherapy adapted to borderline personality disorder + PHA	IPT (adapted from Markowitz, 2005, 2006)	IPT				Markowitz JC (2005) Interpersonal therapy of personality disorders. In: Oldham JM, Skodol AE, Bender BS, editors. Textbook of personality disorders. Washington (DC): American Psychiatric Press; p 321–334.	Content, structure
Bohus, 2020	Dialectical behavior therapy-PTSD	DBT-PTSD (Bohus, 2019)	DBT				Bohus M, Schmahl C, Fydrich T, et al (2019) A research programme to evaluate DBT-PTSD, a modular treatment approach for Complex PTSD after childhood abuse. bord personal disord emot dysregul 6, 7. https://doi.org/10.1186/s40479-019-0099-y	
Bohus, 2020	Cognitive processing therapy	CPT (adapted from Resick, 2016)	CBT				Resick PA, Monson CM, Chard KM (2016) Cognitive Processing Therapy for PTSD: A Comprehensive Manual. Guilford Press	Structure

Trial reference (author, year)	Original Intervention Name	Distinct Intervention Name	Macro-family intervention 1	Macro-family intervention 2	Family intervention 3	Manuals references	Manuals references	Type of Adaptation (content, duration/sessions, cultural, unknown)
Bozzatello, 2020	Interpersonal psychotherapy adapted for treating borderline personality disorder revised	IPT (adapted from Markowitz, 2005, 2006)	IPT				Markowitz JC (2005) Interpersonal therapy of personality disorders. In: Oldham JM, Skodol AE, Bender BS, editors. Textbook of personality disorders. Washington (DC): American Psychiatric Press; p. 321–34. Markowitz JC, Skodol AE, Bleiberg K. (2006) Interpersonal psychotherapy for borderline personality disorder: possible mechanisms of change. J Clin Psychol 62(4):431–44. doi: 10.1002/jclp.20242	
Carlyle, 2020	Mentalization based treatment	MBT-O (Bateman & Fonagy 2004, 2006 described in Bateman, 2010; Bateman & Fonagy 2016)	PSYD				Bateman AW, Fonagy P (2004) Psychotherapy for Mentalization-Based Treatment. Oxford, Oxford University Press. Bateman AW, Fonagy P (2006) 'The structure of mentalization-based treatment', Mentalization-based Treatment for Borderline Personality Disorder: A Practical Guide, International Perspectives in Philosophy & Psychiatry (Oxford; online edn, Oxford Academic, 1 Feb. 2013), https://doi.org/10.1093/med/9780198570905.003.004, accessed 16 Apr. 2025. Bateman A, & Fonagy P (2010) Mentalization based treatment for borderline personality disorder. World Psychiatry, 9: 11-15. https://doi.org/10.1002/j.2051-5545.2010.tb00255.x	
Carlyle, 2020	Enhanced therapeutic case management	SCM (Bateman & Fonagy, 2009, unpublished, described in Bateman & Krawitz, 2013)	SCM				Bateman AW, Krawitz R (2013) Borderline Personality Disorder: An Evidence-Based Guide for Generalist Mental Health Professionals. Oxford: Oxford University Press	
Carter, 2010	Dialectical behavior therapy	DBT (adapted from Linehan, 1993a, 1993b)	DBT				Linehan MM (1993a) Cognitive-behavioral treatment of borderline personality disorder. New York: Guildford	Content, delivery, structure
Clarkin, 2007	Transference-focused psychotherapy	TFP (Clarkin, 1999; Yeomans, 2002)	PSYD				Clarkin JF, Yeomans FE, Kernberg OF (1999) Psychotherapy for Borderline Personality. New York, NY: John Wiley & Sons	
Clarkin, 2007	Dialectical behavior therapy	DBT (adapted from Linehan, 1993a, 1993b)	DBT				Linehan MM (1993a) Cognitive-Behavioral Treatment of Borderline Personality Disorder. New York, Guilford	

Trial reference (author, year)	Original Intervention Name	Distinct Intervention Name	Macro-family intervention 1	Macro-family intervention 2	Family intervention 3	Manuals references	Manuals references	Type of Adaptation (content, duration/sessions, cultural, unknown)
Clarkin, 2007	Supportive therapy	SUP (Rockland, 1992)	SUP	PSYD			Rockland LH (1992) Supportive Therapy for Borderline Patients: A Psychodynamic Approach. New York, Guilford, 1992	
Cottraux, 2009	Cognitive therapy	CT (Cottraux, 2009, unpublished)	CBT	SCH			Cottraux (2009) [Unpublished] Manuel de thérapie cognitive	
Cottraux, 2009	Rogerian supportive therapy	CCT (Cottraux, 2009, unpublished)	SUP	PSYD			Cottraux (2009) [Unpublished] Manuel de thérapie de soutien non directive	
Crawford, 2020	Psychological support for personality	PSP (Crawford, 2018)	SUP				Crawford MJ, Thana L, Parker J, Turner O, Xing KP, McMurran M, Moran P, Weaver T, Barrett B, Claringbold A, Bassett P, & Sanatinia R (2018) Psychological Support for Personality (PSP) versus treatment as usual: study protocol for a feasibility randomized controlled trial of a low intensity intervention for people with personality disorder. Trials, 19(1), 547.	Content, delivery
Davidson, 2006	Cognitive behavior therapy	CBT (Davidson, 2000)	CBT				Davidson KM (2000). Cognitive therapy for personality disorders: A guide for clinicians. London: Butterworth-Heinemann.	
Dixon-Gordon, 2015	Dialectical behavior therapy-emotion regulation skills training	DBT-ST (adapted from Linehan, 2014)	DBT				Linehan MM (1993a) Cognitive behavioral therapy of borderline personality disorder. New York: Guilford Press.<br>Linehan MM (2014) DBT Skills Training Manual (2nd ed.). New York: Guilford Press.	Content
Dixon-Gordon, 2015	Dialectical behavior therapy-interpersonal effectiveness skills training	DBT-ST (adapted from Linehan, 2014)	DBT				Linehan MM (1993a) Cognitive behavioral therapy of borderline personality disorder. New York: Guilford Press.<br>Linehan MM (2014) DBT Skills Training Manual (2nd ed.). New York: Guilford Press.	Content
Dixon-Gordon, 2015	Interpersonal and psycho-education group	SUP (Dixon-Gordon, 2015, unpublished)	SUP				Dixon-Gordon (2015) [Unpublished] Interpersonal + Psychoeducation Group Mini-Manual	
Doering, 2010	Transference-focused psychotherapy	TFP (Clarkin, 1999; Yeomans, 2002)	PSYD				Clarkin JF, Yeomans FE, Kernberg OF (1999) Psychotherapy for Borderline Personality. New York, NY: John Wiley & Sons.<br>Clarkin JF, Yeomans FE, Kernberg OF (2001)	

Trial reference (author, year)	Original Intervention Name	Distinct Intervention Name	Macro-family intervention 1	Macro-family intervention 2	Family intervention 3	Manuals references	Manuals references	Type of Adaptation (content, duration/sessions, cultural, unknown)
							Psychotherapie der Borderline Persönlichkeit. Manual zur psychodynamischen Therapie. Schattauer	
Farrell, 2009	Schema-focused therapy	Group ST (Farrell & Shaw, 1990, unpublished; adapted from Farrell & Shaw, 2012)	SCH				Farrell JM, & Shaw IA (1990) [unpublished]. Emotional awareness training for borderline personality disorder patients: a treatment manual. Unpublished manuscript for NIH RO3, Indiana University School of Medicine, Indianapolis. Farrell JM, & Shaw IA (2012). Group Schema therapy for Borderline Personality Disorder: A Step-by-Step Treatment Manual with Patient Workbook. John Wiley & Sons.	Content, delivery, structure
Feigenbaum, 2012	Dialectical behavior therapy	DBT (adapted from Linehan, 1993a, 1993b)	DBT				Linehan MM (1993a) Cognitive behavioral therapy of borderline personality disorder. New York: Guilford Press	
Giesen-Bloo, 2006	Transference-focused psychotherapy	TFP (Clarkin, 1999; Yeomans, 2002)	PSYD				Clarkin JF, Yeomans FE, Kernberg OF (1999) Psychotherapy for Borderline Personality. New York, NY: John Wiley & Sons. Yeomans FE, Clarkin JF, & Kernberg OF (2002) A primer of transference-focused psychotherapy for the borderline patient. Jason Aronson.	
Giesen-Bloo, 2006	Schema-focused therapy	ST (Young, 1994, 2003)	SCH				Young JE (1994) Cognitive therapy for personality disorders: A schema-focused approach (Rev. ed.). Professional Resource Press/Professional Resource Exchange.. Young JE, Klosko JS, & Weishaar ME (2003) Schema therapy: A practitioner's guide. Guilford Press.	
Gregory, 2008	Dynamic deconstructive psychotherapy	DDP (Gregory & Remen, 2008)	PSYD				Gregory RJ, & Remen AL (2008) A manual-based psychodynamic therapy for treatment-resistant borderline personality disorder. Psychotherapy (Chicago, Ill.), 45(1), 15–27	
Herpertz, 2020	Mechanism-based anti-aggression psychotherapy	MAAP (Herpertz, 2020, unpublished)	DBT	CBT	PSYD		Herpertz (2020) [Unpublished] MAAP intervention manual	
Herpertz, 2020	Non-specific supportive psychotherapy	SUP (Markowitz, 2002, unpublished)	SUP				Markowitz J, Sacks M, Frances A (2002) [Unpublished manual] Non-specific supportive psychotherapy manual	

Trial reference (author, year)	Original Intervention Name	Distinct Intervention Name	Macro-family intervention 1	Macro-family intervention 2	Family intervention 3	Manuals references	Manuals references	Type of Adaptation (content, duration/sessions, cultural, unknown)
Hilden, 2020	Schema group therapy plus treatment as usual	Group ST (Farrell & Shaw, 1990, unpublished; adapted from Farrell & Shaw, 2012)	SCH				Farrell JM, & Shaw IA (2012) Group Schema therapy for Borderline Personality Disorder: A Step-by-Step Treatment Manual with Patient Workbook. John Wiley & Sons.	
Jørgensen, 2012	Combined mentalization-based psychotherapy	MBT-O (Jorgensen, 2009, unpublished; adapted from Bateman & Fonagy, 2004)	PSYD				Jørgensen CR, Kjølbye M, Freund C, et al (2009) [Unpublished] Behandlings-beskrivelse for mentaliseringsbaseret gruppeterapi ved borderline personlighedsforstyrrelser. [Mentalization-based group psychotherapy for borderline personality disorder. Summary of clinical directions]. Clinic for personality disorders, Aarhus University Hospital, Risskov.<br>Bateman A, & Fonagy P (2004) Psychotherapy for Borderline personality Disorder: Mentalization-based Treatment. Oxford University Press, USA	
Jørgensen, 2012	Supportive group therapy	SUP (Jørgensen, 2008, unpublished)	SUP				Jørgensen CR, Freund C, Funk I (2008) Behandlingsbeskrivelse for støttende gruppeterapi. Klinik for personlighedsforstyr-relser, Risskov. [Supportive group treatment. Summary of clinical directions. Clinic for personality disorders, Risskov]. Clinic for personality disorders, Aarhus University Hospital, Risskov (unpublished).	
Koons, 2001	Dialectical behavior therapy	DBT (adapted from Linehan, 1993a, 1993b)	DBT				Linehan MM (1993a) Cognitive behavioral therapy of borderline personality disorder. New York: Guilford Press.<br>Linehan MM (1993b) Skills training manual for treating borderline personality disorder. New York: Guilford Press	
Kredlow, 2017	Cognitive-behavioral therapy	CBT-PTSD (Mueser, 2008, unpublished)	CBT				Mueser (2008) [unpublished] CBT intervention	
Laurenssen, 2018	Day hospital mentalization-based treatment	MBT-DH (Bateman & Fonagy, 2004)	PSYD				Bateman A, & Fonagy P (2004) Psychotherapy for Borderline Personality Disorder: Mentalization-Based Treatment. Oxford: Oxford University Press	
Leppanen, 2015	Community treatment by experts	CTBE (Leppanen, 2015)	SCH	DBT			Leppänen V, Hakko H, Sintonen H, & Lindeman S (2016) Comparing effectiveness of treatments for borderline personality disorder in communal mental health care: The Oulu BPD Study. Community mental health journal, 52, 216-227.	

**Trial reference (author, year)**	**Original Intervention Name**	**Distinct Intervention Name**	**Macro-family intervention 1**	**Macro-family intervention 2**	**Family intervention 3**	**Manuals references**	**Manuals references**	**Type of Adaptation (content, duration/sessions, cultural, unknown)**
Lin, 2019	Dialectic behaviour therapy - skills training	DBT-ST (adapted from Linehan, 1993b)	DBT				Linehan MM (1993b) Skills training manual for treating borderline personality disorder. Guilford Press.	Structure
Lin, 2019	Cognitive therapy group program	CT (adapted from Free, 2007)	CBT				Free ML (2007) Cognitive therapy in groups: Guidelines and resources for practice. John Wiley & Sons.	Structure
Linehan, 1991	Dialectic behavior Therapy	DBT (Linehan, 1984)	DBT				Linehan MM (1984) Dialectical Behavior Therapy for Treatment of Parasuicidal Women: Treatment Manual. Seattle, Wash: University of Washington	
Linehan, 2006	Dialectic behavior Therapy	DBT (adapted from Linehan, 1993a, 1993b)	DBT				Linehan MM (1993a) Cognitive-Behavioral Treatment of Borderline Personality Disorder. New York: The Guilford Press.<br>Linehan MM (1993b) Skills Training Manual for Treating Borderline Personality Disorder. New York: The Guilford Press	
Majdara, 2019	Dynamic deconstructive psychotherapy	DDP (Gregory & Remen, 2008)	PSYD				Gregory RJ, & Remen AL (2008) A manual-based psychodynamic therapy for treatment-resistant borderline personality disorder. Psychotherapy: Theory, Research, Practice, Training, 45(1), 15–27. www.upstate.edu/ddp	
McMain, 2017	Dialectic behavior Therapy	DBT-ST (adapted from Linehan, 2014)	DBT				Linehan MM (2014) DBT Skills Training Manual (2nd ed.). New York: Guilford Press.	
McMain, 2009	Dialectic behavior Therapy	DBT (adapted from Linehan, 1993a, 1993b)	DBT				Linehan MM (1993a) Cognitive-Behavioral Treatment of Borderline Personality Disorder. New York: The Guilford Press.<br>Linehan MM (1993b) Skills Training Manual for Treating Borderline Personality Disorder. New York: The Guilford Press	
McMain, 2009	General psychiatric management	GPM (McMain, 2009, unpublished)	PSYD				McMain SF, Links PS, Gnam WH, Guimond T, Cardish RJ, Korman L, & Streiner DL (2009) [unpublished] General Psychiatric Management: Treatment Manual	
Philips, 2018	Mentalization-based treatment plus SUD treatment	MBT-O (Bateman & Fonagy 2004, 2006 described in	PSYD				Bateman AW, Fonagy P (2004) Psychotherapy for Mentalization-Based Treatment. Oxford, Oxford University Press.	

Trial reference (author, year)	Original Intervention Name	Distinct Intervention Name	Macro-family intervention 1	Macro-family intervention 2	Family intervention 3	Manuals references	Manuals references	Type of Adaptation (content, duration/sessions, cultural, unknown)
		Bateman, 2010; Bateman & Fonagy 2016)					Bateman AW, Fonagy P (2016) Mentalization-based treatment for personality disorders: a practical guide. Oxford, Oxford University Press	
Pistorello, 2012	Dialectic behavior therapy	DBT (adapted from Linehan, 1993a, 1993b)	DBT				Linehan MM (1993a) Cognitive-Behavioral Treatment of Borderline Personality Disorder. New York: The Guilford Press.<br>Linehan MM (1993b) Skills Training Manual for Treating Borderline Personality Disorder. New York: The Guilford Press	
Priebe, 2012	Dialectic behavior therapy	DBT (adapted from Linehan, 1993a, 1993b)	DBT				Linehan MM (1993a) Cognitive-Behavioral Treatment of Borderline Personality Disorder. New York: The Guilford Press.<br>Linehan MM (1993b) Skills Training Manual for Treating Borderline Personality Disorder. New York: The Guilford Press	
Reneses, 2013	Psychic representation focused psychotherapy plus conventional treatment	STDT (Trujillo, 2002, 2010)	PSYD				Trujillo M (2002) The treatment of narcissistic disorders with short-term dynamic psychotherapy. In: Magnavita J, ed. Comprehensive handbook of psychotherapy. New York: John Willey and sons, p. 32-8.<br>Trujillo M (2010) Intensive short-term dynamic psychotherapy. In: Sadok B, Sadok V, Ruiz P, eds. Comprehensive Textbook of Psychiatry. Baltimore: Williams and Wilkins, 2010	
Soler, 2009	Dialectic behavior therapy - skills training	DBT-ST (adapted from Linehan, 1993b)	DBT				Linehan MM (1993b) Skills Training Manual for Treating Borderline Personality Disorder. New York: The Guilford Press	
Soler, 2009	Standard group therapy	SGT (not manualized)	PSYD				Not manualized	
Stanley, 2017	Dialectical behavior therapy	DBT (Linehan, 1993a, 2014)	DBT				Linehan MM (1993a) Cognitive behavioral treatment of BPD. New York: Guilford.<br>Linehan MM (2014) DBT Skills Training Manual (2nd ed.). New York: Guilford Press.	
Turner, 2000	Dialectical behavior therapy	DBT-E (Turner, 2000; adapted from Linehan, 1993a)	DBT	PSYD			Turner RM (2000) Naturalistic evaluation of dialectical behavior therapy-oriented treatment for borderline personality disorder. Cognitive and Behavioral Practice;7(4),413–419.<br>Linehan MM (1993a) Cognitive-Behavioral	Content

Trial reference (author, year)	Original Intervention Name	Distinct Intervention Name	Macro-family intervention 1	Macro-family intervention 2	Family intervention 3	Manuals references	Manuals references	Type of Adaptation (content, duration/sessions, cultural, unknown)
							Treatment of Borderline Personality Disorder. New York: The Guilford Press.	
Turner, 2000	Client-centered therapy control condition	CCT (Carkhuff, 1969; Carkhuff, 1976)	SUP				Carkhuff RR (1969) Helping and human relations. New York: Holt, Rinehart, & Winston. Carkhuff RR, Pierce R, & Cannon J (1976) The art of helping.	
Verheul, 2003	Dialectical behavior therapy	DBT (adapted from Linehan, 1993a, 1993b)	DBT				Linehan MM (1993a) Cognitive-Behavioral Treatment of Borderline Personality Disorder. New York: The Guilford Press.	
Visintini, 2020	Dialectical behavior therapy	DBT (Linehan, 1993a, 2014)	DBT				Linehan MM (1993a) Cognitive-Behavioral Treatment of Borderline Personality Disorder. New York: The Guilford Press. Linehan MM (2014) DBT Skills Training Manual (2nd ed.). New York: Guilford Press.	
Visintini, 2020	Gruppi esperienziali terapeutici	GET (Visintini, 2020, unpublished)	PSYD	CBT			Visintini (2019) [unpublished] GET intervention	Content, structure
Walton, 2020	Dialectical behavior therapy	DBT (adapted from Linehan, 1993a, 1993b)	DBT				Linehan MM (1993a) Cognitive-Behavioral Treatment of Borderline Personality Disorder. New York: The Guilford Press. Linehan MM (1993b) Skills Training Manual for Treating Borderline Personality Disorder. New York: The Guilford Press	
Walton, 2020	Conversational model	CMT (Meares, 2012)	PSYD				Meares R (2012) Borderline Personality Disorder and the Conversational Model: A Clinician's Manual. New York: W. W. Norton & Company.	
Weinberg, 2006	Manual assisted cognitive treatment	MACT (Schmidt & Davidson, 2004)	CBT	DBT			Schmidt U, & Davidson K (2004) Life after self-harm. Hove, East Essex: Brunner-Routledge.	Content

List of acronyms for interventions and families: AdP: Adlerian Psychotherapy. AP: Abandonment Psychotherapy. CAMS: Collaborative Assessment and Management of Suicidality treatment. CBT: Cognitive-Behavioural Therapy. CBT-PTSD: Cognitive Behavioral Therapy for Post-traumatic Stress Disorder. CCT: Client-Centered Therapy. CMT: Conversational Model Therapy. CPT: Cognitive Processing Therapy. CT: Cognitive Therapy. CTBE: Community Treatment by Experts. DBT: Dialectic Behaviour Therapy. DBT-E: Dialectical Behavioral Therapy enhanced with psychodynamic techniques. DBT-ER: Dialectical Behavioral Therapy – Emotion Regulation module. DBT-IE: Dialectical Behavioral Therapy - Interpersonal Effectiveness module. DBT-PTSD: Dialectical Behavioral Therapy for Post-traumatic Stress Disorder. DBT-ST: Dialectic Behaviour Therapy - Skills Training. DDP: Dynamic Deconstructive Psychotherapy. GET: Gruppi Esperienziali Terapeutici. GPM: General Psychiatric Management. IPT: Interpersonal psychotherapy. MAAP: Mechanism-based anti-aggression psychotherapy. MACT: Manual Assisted Cognitive Treatment. MBT-DH: Mentalization-Based Treatment - Day Hospital. MBT-O: Mentalization-Based Treatment – Outpatient. PHA: Pharmacotherapy. PSP: Psychological Support for Personality. PSYD: Psychodynamic Psychotherapy. SCM: Structured Clinical Management. SGT: Standard Group Therapy. SCH: Schema Therapy. STDT: Short-term dynamic therapy. STM: Supervised team management. SUD: Substance Use DisorderSUP: Supportive Therapy. TFP: Transference-Focused Therapy.

Tomei G, *et al. BMJ Ment Health* 2026; 29:e302555. doi: 10.1136/bmjment-2026-302555

Table S8. Total psychological intervention arms with macro-family and adaptations – Bulimia nervosa

Trial reference (author, year)	Original Intervention Name	Distinct Intervention Name	Macro-family intervention 1	Macro-family intervention 2	Manuals references	Type of Adaptation (content, duration/sessions, cultural, unknown)
Agras, 2000	Cognitive-behavioral therapy	CBT (Fairburn, 1993)	CBT		Fairburn CG, Marcus MD, Wilson GT. (1993) Cognitive behavioral therapy for binge eating and bulimia nervosa: a comprehensive treatment manual. In: Fairburn CG, Wilson GT eds. Binge Eating: Nature, Assessment, and Treatment. New York, NY: Guilford Press:361-404.	
Agras, 2000	Interpersonal psychotherapy	IPT (Fairburn, 1997)	IPT		Fairburn CG. (1997) Interpersonal therapy for bulimia nervosa. In: Garner DM, Garfinkel PE, eds. Handbook of Treatment for Eating Disorders. New York, NY: Guilford Press	
Bailer, 2004	Group Cognitive-behavioral therapy	Group CBT (Jacobi, 1996)	CBT		Jacobi C, Thiel A, & Paul T (1996) Kognitive Verhaltenstherapie bei Bulimia und Anorexia nervosa. Weinheim, Germany: Beltz Psychologie Verlags Union.	Delivery
Bailer, 2004	Cognitive-behavioral therapy self-help	CBT self-help (Schmidt & Treasure, 1993)	SHP		Schmidt UH, & Treasure JL (1993) Getting better bit(e) by bit(e). London: Erlbaum	Delivery
Banasiak, 2005	Cognitive-behavioral therapy self-help	CBT self-help (Cooper, 1995)	SHP		Cooper PJ (1995) Bulimia Nervosa and Binge-Eating: A Guide to Recovery (revised edn). Robinson Publishing Ltd: London.	Delivery
Bulik, 1998	Cognitive-behavioral therapy + Binge Exposure and response prevention	CBT (Bulik, 1993; unpublished) + ERP (Bulik, 1993; unpublished)	CBT	ERP	Bulik C, Sullivan PF, Carter FA, Joyce PR (1993) [Unpublished] Cognitive Therapy Therapist Manual for the treatment of Bulimia Nervosa. Bulik C, Sullivan PF, Carter FA, Joyce PR (1993) [Unpublished]Bulimia Treatment Study B-ERP Therapist Manual	Content
Bulik, 1998	Cognitive-behavioral therapy + Purge Exposure and response prevention	CBT (Bulik, 1993; unpublished) + ERP (Bulik, 1993; unpublished)	CBT	ERP	Bulik C, Sullivan PF, Carter FA, Joyce PR (1993) [Unpublished] Cognitive Therapy Therapist Manual for the treatment of Bulimia Nervosa. Bulik C, Sullivan PF, Carter FA, Joyce PR (1993) [Unpublished] Bulimia Treatment Study P-ERP therapist Manual	Content
Bulik, 1998	Cognitive-behavioral therapy + relaxation	CBT (Bulik, 1993; unpublished) + Relaxation (not manualized)	CBT	REL	Bulik C, Sullivan PF, Carter FA, Joyce PR (1993) [Unpublished]Cognitive Therapy Therapist Manual for the treatment of Bulimia Nervosa	
Cooper, 1995	Cognitive-behavioral therapy	CBT (Fairburn & Cooper, 1989)	CBT		Fairburn CG, & Cooper PJ (1989) Eating disorders. In Hawton K, Salkovskis PM, Kirk J, & Clark DM. (Eds), Cognitive behaviour therapy for psychiatric problems. Oxford Univ. Press.	Content
Cooper, 1995	Cognitive-behavioral therapy + Exposure and response prevention	CBT (Fairburn & Cooper, 1989) + ERP (Adapted from Rosen & Leitenberg, 1985)	CBT	ERP	Fairburn CG, & Cooper PJ (1989) Eating disorders In Hawton K, Salkovskis PM, Kirk J, & Clark DM. (Eds), Cognitive behaviour therapy for psychiatric problems. Oxford Univ. Press. Rosen, J. C., & Leitenberg, H. (1982). Bulimia nervosa: Treatment	Content

Trial reference (author, year)	Original Intervention Name	Distinct Intervention Name	Macro-family intervention 1	Macro-family intervention 2	Manuals references	Type of Adaptation (content, duration/sessions, cultural, unknown)
					with exposure and response prevention. Behavior Therapy, 13(1), 117–124.<br>Rosen, J. C., & Leitenberg, H. (1982). Bulimia nervosa: Treatment with exposure and response prevention. Behavior Therapy, 13(1), 117-124	
Fairburn, 1993	Behavioral treatment	CBT (Fairburn, 1993)	CBT		Fairburn CG, Marcus MD, Wilson GT. (1993) Cognitive behavioral therapy for binge eating and bulimia nervosa: a comprehensive treatment manual. In: Fairburn CG, Wilson GT eds. Binge Eating: Nature, Assessment, and Treatment. New York, NY: Guilford Press:361-404.	Content
Fairburn, 1993	Cognitive-behavioral therapy	CBT (Fairburn, 1993)	CBT		Fairburn CG, Marcus MD, Wilson GT. (1993) Cognitive behavioral therapy for binge eating and bulimia nervosa: a comprehensive treatment manual. In: Fairburn CG, Wilson GT eds. Binge Eating: Nature, Assessment, and Treatment. New York, NY: Guilford Press:361-404.	
Fairburn, 1993	Interpersonal psychotherapy	IPT (Klerman, 1984)	IPT		Klerman GL, Weissman MM, Rounsaville BJ, Chevron ES. (1984) Interpersonal Psychotherapy of Depression. New York, NY: Basic Books Inc Publishers.<br>Fairburn, CG (1985) Cognitive-behavioral treatment for bulimia. In D. M. Garner & P. E. Garfinkel (Eds.), Handbook of psychotherapy for anorexia nervosa and bulimia. New York: Guilford Press.	Content
Fairburn, 2009	Enhanced Cognitive-behavioral therapy-focused	CBT-E-f (Fairburn, 2008)	CBT		Fairburn CG, Cooper Z, Shafran R, Bohn K, Hawker DM, Murphy R, Straebler S (2008) Enhanced cognitive behavior therapy for eating disorders: the core protocol, in Cognitive Behavior Therapy and Eating Disorders. Edited by Fairburn CG. New York, Guilford, 2008	
Fairburn, 2009	Enhanced Cognitive-behavioral therapy-broad	CBT-E-b (Fairburn, 2008)	CBT		Fairburn CG, Cooper Z, Shafran R, Bohn K, Hawker DM, Murphy R, Straebler S (2008) Enhanced cognitive behavior therapy for eating disorders: the core protocol, in Cognitive Behavior Therapy and Eating Disorders. Edited by Fairburn CG. New York, Guilford.<br>Fairburn CG, Cooper Z, Shafran R, Bohn K, Hawker DM (2008) Clinical perfectionism, core low self-esteem, and interpersonal problems, in Cognitive Behavior Therapy and Eating Disorders. Edited by Fairburn CG. New York, Guilford	
Fairburn, 2015	Enhanced Cognitive-behavioral therapy-focused	CBT-E-f (Fairburn, 2008)	CBT		Fairburn CG, Cooper Z, Shafran R, Bohn K, Hawker DM, Murphy R, Straebler S (2008) Enhanced cognitive behavior therapy for eating disorders: the core protocol, in Cognitive Behavior Therapy	

Trial reference (author, year)	Original Intervention Name	Distinct Intervention Name	Macro-family intervention 1	Macro-family intervention 2	Manuals references	Type of Adaptation (content, duration/sessions, cultural, unknown)
					and Eating Disorders. Edited by Fairburn CG. New York, Guilford, 2008	
Fairburn, 2015	Interpersonal psychotherapy	IPT (Murphy, 2012)	IPT		Murphy R, Straebler S, Basden S, Cooper Z, Fairburn CG (2012) Interpersonal Psychotherapy for Eating Disorders. Clin. Psychol. Psychother., 19: 150-158. https://doi.org/10.1002/cpp.1780	
Ghaderi, 2004	Cognitive-behavioral therapy	CBT (Fairburn, 1993)	CBT		Fairburn CG, Marcus MD, Wilson GT. (1993) Cognitive behavioral therapy for binge eating and bulimia nervosa: a comprehensive treatment manual. In: Fairburn CG, Wilson GT eds. Binge Eating: Nature, Assessment, and Treatment. New York, NY: Guilford Press:361-404.	
Ghaderi, 2004	Cognitive-behavioral therapy + Functional analysis	CBT (Fairburn, 1993) + Functional analysis (Adapted from Ghaderi, 2007)	CBT	FUN	Fairburn CG, Marcus MD, Wilson GT. (1993) Cognitive behavioral therapy for binge eating and bulimia nervosa: a comprehensive treatment manual. In: Fairburn CG, Wilson GT eds. Binge Eating: Nature, Assessment, and Treatment. New York, NY: Guilford Press:361-404.<br>Ghaderi, A. (2007) Logical functional analysis in the assessment and treatment of eating disorders. Clinical Psychologist 11(1).	
Goldbloom, 1997	Cognitive-behavioral therapy	CBT (Fairburn, 1993)	CBT		Fairburn CG, Marcus MD, Wilson GT. (1993) Cognitive behavioral therapy for binge eating and bulimia nervosa: a comprehensive treatment manual. In: Fairburn CG, Wilson GT eds. Binge Eating: Nature, Assessment, and Treatment. New York, NY: Guilford Press.<br>Fawcett J, Epstein P, Fiester S J, Elkin I, & Autry JH (1987) Clinical management--imipramine/placebo administration manual. Psvchopharmacology Bulletin, 23, 309 324.	
Goldbloom, 1997	Cognitive-behavioral therapy	CBT (Fairburn, 1993)	CBT		Fairburn CG, Marcus MD, Wilson GT. (1993) Cognitive behavioral therapy for binge eating and bulimia nervosa: a comprehensive treatment manual. In: Fairburn CG, Wilson GT eds. Binge Eating: Nature, Assessment, and Treatment. New York, NY: Guilford Press:361-404.	
Jacobi, 2002	Cognitive-behavioral therapy	CBT (Fairburn, 1985; Adapted Jacobi, 1996)	CBT		Fairburn, CG (1985) Cognitive-behavioral treatment for bulimia. In D. M. Garner & P. E. Garfinkel (Eds.), Handbook of psychotherapy for anorexia nervosa and bulimia. New York: Guilford Press.<br>Agras WS. (1987) Eating Disorders. Management of Obesity, Bulimia and Anorexia Nervosa. Pergamon Press: Oxford.<br>Jacobi C, Thiel A, & Paul T (1996) Kognitive Verhaltenstherapie	Unknown

Trial reference (author, year)	Original Intervention Name	Distinct Intervention Name	Macro-family intervention 1	Macro-family intervention 2	Manuals references	Type of Adaptation (content, duration/sessions, cultural, unknown)
					bei Bulimia und Anorexia nervosa. Weinheim, Germany: Beltz Psychologie Verlags Union.	
Jacobi, 2002	Cognitive-behavioral therapy	CBT (Fairburn, 1985; Adapted Jacobi, 1996)	CBT		Fairburn, CG (1985) Cognitive-behavioral treatment for bulimia. In D. M. Garner & P. E. Garfinkel (Eds.), Handbook of psychotherapy for anorexia nervosa and bulimia. New York: Guilford Press. Agras WS. (1987) Eating Disorders. Management of Obesity, Bulimia and Anorexia Nervosa. Pergamon Press: Oxford. Jacobi C, Thiel A, & Paul T (1996) Kognitive Verhaltenstherapie bei Bulimia und Anorexia nervosa. Weinheim, Germany: Beltz Psychologie Verlags Union.	Unknown
Lee & Rush, 1986	Group Cognitive-behavioral therapy	Group CBT (Kumetz-Lee, 1986; unpublished)	CBT		Kumetz-Lee N (1986) [Unpublished] Group Cognitive Behavioral Intervention	
Leitenberg, 1988	Cognitive-behavioral therapy	CBT (Fairburn, 1985; Adapted Leitenberg, 1988)	CBT		Fairburn CG (1985) Cognitive-behavioral treatment for bulimia. In D. M. Garner & P. E. Garfinkel (Eds.), Handbook of psychotherapy for anorexia nervosa and bulimia. New York: Guilford Press.	Delivery
Leitenberg, 1988	Cognitive-behavioral therapy + Multiple-setting Exposure and response prevention	CBT (Fairburn, 1985) + ERP (Leitenberg, 1988)	CBT	ERP	Fairburn CG (1985) Cognitive-behavioral treatment for bulimia. In D. M. Garner & P. E. Garfinkel (Eds.), Handbook of psychotherapy for anorexia nervosa and bulimia. New York: Guilford Press. Leitenberg H, Rosen JC, Gross J, Nudelman S, Vara LS (1988) Exposure Plus Response-Prevention Treatment of Bulimia Nervosa. Journal of Consulting and Clinical Psychology 56(4): 535-541	Content, Delivery
Leitenberg, 1988	Cognitive-behavioral therapy + Single-setting Exposure and response prevention	CBT (Fairburn, 1985) + ERP (Leitenberg, 1988)	CBT	ERP	Fairburn CG (1985) Cognitive-behavioral treatment for bulimia. In D. M. Garner & P. E. Garfinkel (Eds.), Handbook of psychotherapy for anorexia nervosa and bulimia. New York: Guilford Press. Leitenberg H, Rosen JC, Gross J, Nudelman S, Vara LS (1988) Exposure Plus Response-Prevention Treatment of Bulimia Nervosa. Journal of Consulting and Clinical Psychology 56(4): 535-541	Content, Delivery
Mitchell, 2008	Cognitive-behavioral therapy	CBT (Fairburn, 1993)	CBT		Fairburn CG, Marcus MD, Wilson GT. (1993) Cognitive behavioral therapy for binge eating and bulimia nervosa: a comprehensive treatment manual. In: Fairburn CG, Wilson GT eds. Binge Eating: Nature, Assessment, and Treatment. New York, NY: Guilford Press:361-404.	

Trial reference (author, year)	Original Intervention Name	Distinct Intervention Name	Macro-family intervention 1	Macro-family intervention 2	Manuals references	Type of Adaptation (content, duration/sessions, cultural, unknown)
Mitchell, 2008	Cognitive-behavioral therapy	CBT (Fairburn, 1993)	CBT		Fairburn CG, Marcus MD, Wilson GT. (1993) Cognitive behavioral therapy for binge eating and bulimia nervosa: a comprehensive treatment manual. In: Fairburn CG, Wilson GT eds. Binge Eating: Nature, Assessment, and Treatment. New York, NY: Guilford Press:361-404.	
Palmer, 2002	Cognitive-behavioral therapy self-help + in-person guidance	CBT self-help (Fairburn, 1995)	SHP		Fairburn CG (1995) Overcoming Binge Eating. Overcoming Binge Eating.New York: Guilford Press.	Delivery
Palmer, 2002	Cognitive-behavioral therapy self-help + remote guidance	CBT self-help (Fairburn, 1995)	SHP		Fairburn CG (1995) Overcoming Binge Eating. Overcoming Binge Eating.New York: Guilford Press.	Delivery
Palmer, 2002	Cognitive-behavioral therapy self-help	CBT self-help (Fairburn, 1995)	SHP		Fairburn CG (1995) Overcoming Binge Eating. Overcoming Binge Eating.New York: Guilford Press.	
Sanchez-Ortiz, 2011	Cognitive-behavioral therapy self-help	CBT self-help (Williams, 1998)	CBT		Williams C, Aubin S, Cottrell D, Harkin PJA (1998) Overcoming bulimia: a self-help package. [Computer software]. Leeds, University of Leeds	
Thompson-Brenner, 2016	Enhanced Cognitive-behavioral therapy-focused	CBT-E-f (Fairburn, 2008)	CBT		Fairburn CG, Cooper Z, Shafran R, Bohn K, Hawker DM, Murphy R, Straebler S (2008) Enhanced cognitive behavior therapy for eating disorders: the core protocol, in Cognitive Behavior Therapy and Eating Disorders. Edited by Fairburn CG. New York, Guilford	
Thompson-Brenner, 2016	Enhanced Cognitive-behavioral therapy-broad	CBT-E-b (Fairburn, 2008)	CBT		Fairburn CG, Cooper Z, Shafran R, Bohn K, Hawker DM, Murphy R, Straebler S (2008) Enhanced cognitive behavior therapy for eating disorders: the core protocol, in Cognitive Behavior Therapy and Eating Disorders. Edited by Fairburn CG. New York, Guilford. Fairburn CG, Cooper Z, Shafran R, Bohn K, Hawker DM (2008) Clinical perfectionism, core low self-esteem, and interpersonal problems, in Cognitive Behavior Therapy and Eating Disorders. Edited by Fairburn CG. New York, Guilford	
Treasure, 1994	Cognitive-behavioral therapy self-help	CBT self-help (Schmidt & Treasure, 1993)	SHP		Schmidt UH, & Treasure JL (1993) Getting better bit(e) by bit(e). London: Erlbaum	
Treasure, 1994	Cognitive-behavioral therapy	CBT (Fairburn & Cooper, 1989)	CBT		Fairburn CG, & Cooper PJ (1989) Eating disorders. In Hawton K, Salkovskis PM, Kirk J, & Clark DM. (Eds), Cognitive behaviour therapy for psychiatric problems. Oxford Univ. Press.	Structure
Wagner, 2013	Cognitive-behavioral therapy self-help	CBT self-help (Schmidt & Treasure, 1993)	SHP		Schmidt UH, & Treasure JL (1993) Getting better bit(e) by bit(e). London: Erlbaum	
Wagner, 2013	Cognitive-behavioral therapy self-help	CBT self-help (Netunion, 2013)	SHP		https://www.netunion.com/salut-bn	

Trial reference (author, year)	Original Intervention Name	Distinct Intervention Name	Macro-family intervention 1	Macro-family intervention 2	Manuals references	Type of Adaptation (content, duration/sessions, cultural, unknown)
Walsh, 1997	Supportive psychotherapy	Supportive psychotherapy (Fairburn 1986; Adapted Walsh, 1997)	SUP		Fairburn CG, Kirk J, O'Connor M, Cooper PJ (1986) A comparison of two psychological treatments for bulimia nervosa. Behav Res Ther 24.<br>Walsh BT, Wilson GT, Loeb KL, Devlin MJ, Pike KM, Roose SP, Fleiss J, Waternaux C. Medication and psychotherapy in the treatment of bulimia nervosa. Am J Psychiatry. 1997 Apr;154(4):523-31	Content
Walsh, 1997	Supportive psychotherapy	Supportive psychotherapy (Fairburn 1986; Adapted Walsh, 1997)	SUP		Fairburn CG, Kirk J, O'Connor M, Cooper PJ (1986) A comparison of two psychological treatments for bulimia nervosa. Behav Res Ther 24.<br>Walsh BT, Wilson GT, Loeb KL, Devlin MJ, Pike KM, Roose SP, Fleiss J, Waternaux C. Medication and psychotherapy in the treatment of bulimia nervosa. Am J Psychiatry. 1997 Apr;154(4):523-31	Content
Walsh, 1997	Cognitive-behavioral therapy	CBT (Walsh, 1997)	CBT		Walsh TB, Wilson TG, Loeb KL, Devlin MJ, Pike KM, Roose SP, Fleiss J, Waternaux C (1997) Medication and Psychotherapy in the Treatment of Bulimia Nervosa. Am J Psychiatry 154	Content
Walsh, 1997	Cognitive-behavioral therapy	CBT (Walsh, 1997)	CBT		Walsh TB, Wilson TG, Loeb KL, Devlin MJ, Pike KM, Roose SP, Fleiss J, Waternaux C (1997) Medication and Psychotherapy in the Treatment of Bulimia Nervosa. Am J Psychiatry 154	Content
Wilson, 1991	Cognitive-behavioral therapy	CBT (Wilson, 1991; unpublished)	CBT		Wilson GT (1991) [Unpublished] Cognitive Behavioral Intervention	
Wilson, 1991	Cognitive-behavioral therapy + Exposure and response prevention	CBT (Wilson, 1991; unpublished) + ERP (Wilson, 1986)	CBT	ERP	Wilson GT (1991) [Unpublished] Cognitive Behavioral Intervention.<br>Wilson GT, Rossiter E, Kleifield EI, Lindholm L (1986) Cognitive-behavioral treatment of bulimia nervosa: A controlled evaluation. Behaviour Research and Therapy, 24(3), 277–288. doi:10.1016/0005-7967(86)90187-7	

List of acronyms for interventions and families: CBT: Cognitive-Behavioral Therapy. CBT-E-b: Enhanced Cognitive-Behavioral-Therapy – broad. CBT-E-f: Enhanced Cognitive-Behavioral-Therapy – focused. ERP: Exposure and Response Prevention. FA: Functional Analysis. IPT: Interpersonal Psychotherapy. REL: Relaxation. SHP: Self-Help. SUP: Supportive Psychotherapy

Table S9. Total psychological intervention arms with macro-family and adaptations – Schizophrenia and psychotic

Trial reference (author, year)	Original Intervention Name	Distinct Intervention Name	Macro-family intervention 1	Macro-family intervention 2	Manuals references	Type of Adaptation (content, duration/sessions, cultural, unknown)
Aghotor, 2010	Metacognitive training (group)	MCT (Moritz, 2005)	MCT		Moritz, S., Burlon, M., & Woodward, T. S. (2005). Metacognitive training for schizophrenic patients. Hamburg, Germany: VanHam Campus Verlag.	
Andreou, 2017	Metacognitive training (individual)	MCT+ (Moritz, 2011)	CR		Moritz S, Veckenstedt R, Randjbar S, Vitzthum F. (2011) Individualisiertes metakognitives Therapieprogramm für Menschen mit Psychose. Springer	
Andreou, 2017	Computerized cognitive remediation training	CR - CogPack v 5.9 (Marker, 2003)	MCT		Marker K. (2003) COGPACK manual version 5.9. Marker software (Ladenburg).	
Bark, 2003	Computerized cognitive remediation training	CR - Broderbund Software v 2.0 (adapted from Medalia, 2000, Medalia, 2001)	CR		Medalia A, Revheim N, Casey M. (2000) Remediation of memory disorders in schizophrenia. Psychol. Med. 30, 1451– 1459.doi: 10.1017/s0033291799002913.	Content
Barrowclough, 2006	Cognitive behavioral therapy	CBT (Barrowclough, 2006)	CBT		Barrowclough C, Haddock G, Lobban F, Jones S, Siddle RON, Roberts C, et al.(2006) Group cognitive-behavioural therapy for schizophrenia: Randomised controlled trial. Br J Psychiatry. (189):527–32.	
Bechdolf, 2004	Cognitive behavioral therapy	CBT (adapted from Tarrier, 1990, Tarrier 1993)	CBT		Tarrier N, Harwood S, Yusopoff L, Beckett R, Baker A. (1990) Coping Strategy Enhancement (CSE): A Method of Treating Residual Schizophrenic Symptoms. Behavioural Psychotherapy. 18(4). doi: 10.1017/S0141347300010387.	Content
Bechdolf, 2004	Psychoeducation	PSED (adapted from Hornung 1996)	PSED		Hornung WP, Kieserg A, Feldmann R. (1996). Psychoeducational training for schizophrenic patients: background, procedure and empirical findings. Patient Educ Couns, 29:257–268. doi: 10.1016/s0738-3991(96)00918-4.	Content, Structure
Bradley, 2006	Multifamily group treatment	MFT (McFarlane 1991)	MFT		McFarlane WR, Deakins SM, GingerichSL, et al. (1991). Multiple-Family Psychoeducational Group Treatment Manual. NewYork, New York State Psychiatric Institute.	
Buonocore, 2015	Metacognitive training (group)	MCT (Moritz, 2005)	CR		Moritz, S., Burlon, M., & Woodward, T. S. (2005). Metacognitive training for schizophrenic patients. Hamburg, Germany: VanHam Campus Verlag.	
Buonocore, 2015	Computerized cognitive remediation training	CR - CogPack (Marker, 1987-2007)	MCT		Marker, K. (2003). COGPACK manual version 5.9. Marker software (Ladenburg).	
Byrne, 2013	Computerized cognitive remediation training	CR - CDT software (Byrne, 2013)	CR		Byrne LK, Peng D, McCabe M, Mellor D, Zhang J, Zhang T, et al. (2013). Does practice make perfect? Results from a Chinese feasibility study of cognitive remediation in schizophrenia. Neuropsychol Rehabilitation, 23(4):580–96.	
Cai, 2015	Comprehensive family therapy	SST (Liberman, 1993) + family PSED (Cai, 2015)	SST	PSED	Liberman RP, Wallace CJ, Blackwell G, Kopelowicz A, Vaccaro JV,Mintz J. (1998). Skills training versus psychosocial occupational therapy for persons with persistent schizophrenia. Am J Psychiatry. 155(8):1087–1091.	
Chan, 2009	Family and individual psychoeducation	PSED (Chan, 2009) +TAU	PSED		Chan SW, Yip B, Tso S, Cheng B, Tam W. (2009). Evaluation of a psychoeducation program for Chinese clients with schizophrenia and their family caregivers. Patient Educ Couns. 75:67–76. doi: 10.1016/j.pec.2008.08.028	Structure
Chien, 2013a	Mindfulness-based psychoeducation	MF-based PSED (Chien, 2013a)	MF-PSED		Chien WT, Lee IY. (2013). The mindfulness-based psychoeducation program for Chinese patients with schizophrenia. Psychiatr Serv. 64(4), 376–379. doi: 10.1176/appi.ps.002092012.	
Chien, 2013b	Psychoeducation	PSED (Chien, 2013b)	PSED		Chien W-T, Leung SF. (2013). A controlled trial of a needs-based, nurse-led psychoeducation programme for Chinese patients with first-onset mental disorders: 6 month follow up. Int J Nurs Pract.19:3–13.	
Chien, 2014	Mindfulness-based psychoeducation	MF-based PSED (Chien, 2013a)	MF-PSED		Chien WT, Lee IY. (2013). The mindfulness-based psychoeducation program for Chinese patients with schizophrenia. Psychiatr Serv. 64(4), 376–379. doi: 10.1176/appi.ps.002092012.	
Chien, 2014	Psychoeducation	PSED (Chien, 2013b)	PSED		Chien W-T, Leung SF. (2013). A controlled trial of a needs-based, nurse-led psychoeducation programme for Chinese patients with first-onset mental disorders: 6 month follow up. Int J Nurs Pract.19:3–13.	

Trial reference (author, year)	Original Intervention Name	Distinct Intervention Name	Macro-family intervention 1	Macro-family intervention 2	Manuals references	Type of Adaptation (content, duration/sessions, cultural, unknown)
d'Amato, 2011	Computerized cognitive remediation training	CR - RehaCom (Schuhfried, 2003)	CR		Schuhfried G. (2003). RehaCom Version 5. Basic Manual [Internet]. Magdeburg (DE): Hasomed.	
de Mamani	Culturally informed family therapy	Culturally informed FT (Weisman, 2005)	FT		Weisman A. (2005). Integrating culturally-based approaches with existing interventions for Hispanic/Latino families coping with schizophrenia. Psychotherapy: Theory, Research, Practice, Training. 42, 178–197. doi:10.1037/0033-3204.42.2.178	
de Mamani	Standard psychoeducation	PSED (Weisman, 2005)	PSED		Weisman A. (2005). Integrating culturally-based approaches with existing interventions for Hispanic/Latino families coping with schizophrenia. Psychotherapy: Theory, Research, Practice, Training. 42, 178–197. doi:10.1037/0033-3204.42.2.178	
Dickinson, 2010	Computerized cognitive remediation training	CR DCA-CR software (Bellack, 2004)	CR		Bellack AS, Dickinson D, Morris S, Gold J, Tenhula W. (2004) [unpublished]. Maryland computer assisted cognitive remediation manual.	
Durham, 2003	Cognitive behavioral therapy	CBT (Tarrier 1992; Kingdon & Turkington, 1994)	CBT		Tarrier N. (1992). Management and modification of residual psychotic symptoms. In Innovations in the Psychological Management of Schizophrenia (eds M. Birchwood & N.Tarrier), pp.147-170.Chichester: John Wiley & Sons.	
Durham, 2003	Supportive psychotherapy	Psychodynamic psychotherapy (adapted from Garfield, 1995)	SUP		Garfield D. (1995). Unbearable Affect: A Guide to the Psychotherapy of Psychosis. Chichester: JohnWiley & Sons.	Unknown
England, 2007	Cognitive intervention	Cognitive nursing intervention (England, 2006)	CBT		England M. (2006). Cognitive intervention for voice hearers. Issues in Mental Health Nursing. 27(7), 735–751.doi:10.1080/01612840600781139	
Fardig, 2011	Illness management and recovery	Illness management and recovery (Gingerich, S., 2005)	ILLMNG		Gingerich S & Mueser KT. (2005). Illness Management and Recovery. In R. E. Drake, M. R. Merrens, & D. W. Lynde (Eds.), Evidence-based mental health practice: A textbook, pp. 395–424. W. W. Norton & Company	
Farreny, 2012	Group cognitive remediation	CR (Farreny, 2010)	CR		Farreny A. (2010). REPYFLEC (Resolución de Problemas y Flexibilidad Cognitiva): onlinemanual	
Fernandez-Gonzalo, 2015	Computerized cognitive remediation training	CR - NeuroPersonalTrainer® (Cardoner, 2017)	CR		Cardoner N. (2017). Neuropersonaltrainer-mh: A New Computerized Platform for the Cognitive Remediation in Schizophrenia and Bipolar Disorders. European Psychiatry, Volume 41 Issue S1: Abstract of the 25th European Congress of Psychiatry April 2017, pp. S23	
Fiszdon, 2016	Computerized cognitive remediation training	CR - PSS CogReHab (Fiszdon, 2016)	CR		Fiszdon JM, Choi KH, Bell MD, Choi J, Silverstein SM. (2016). Cognitive remediation for individuals with psychosis: efficacy and mechanisms of treatment effects. Psychol Med. 2016;46:3275–89.	
Freeman, 2015a	Cognitive behavioral therapy for worry reduction	CBT - worry (Freeman, 2012)	CBT		Freeman D, Dunn G, Startup H, Kingdon D. (2012). The effects of reducing worry in patients with persecutory delusions: study protocol for a randomized controlled trial. Trials. 13:223. doi: 10.1186/1745-6215-13-223	
Freeman, 2015b	Cognitive behavioral therapy for sleep	CBT - sleep (Freeman, 2013)	CBT		Freeman D, Startup H, Myers E. (2013). The effects of using cognitive behavioural therapy to improve sleep for patients with delusions and hallucinations (the BEST study): study protocol. Trials. 14:214. doi: 10.1186/1745-6215-14-214	
Garcia, 2003	Social perception program group	SCR - IntPT (adapted from Roder, 1996)	SCR		Roder V, Brenner HD, Hodel B, Kienzle N. (1996). Terapia Integrada de la Esquizofrenia. Barcelona: Ariel	Content
Garety, 2008	Cognitive behavioral therapy - no carers	CBT (adapted from Fowler, 1995)	CBT		Fowler D, Garety PA, Kuipers L. (1995). Cognitive Behaviour Therapy for Psychosis: Theory and Practice. Wiley	Content
Garety, 2008	Family intervention - with carers	FT (adapted from Kuipers, 2002)	CBT		Kuipers E, Leff JP, Lam D. (2002). Family Work for Schizophrenia: A Practical Guide(2nd ed). Gaskell.	Content
Garety, 2008	Cognitive behavioral therapy - with carers	CBT (adapted from Fowler, 1995)	FT		Fowler D, Garety PA, Kuipers L. (1995). Cognitive Behaviour Therapy for Psychosis: Theory and Practice. Wiley	Content
GilSanz, 2009	Social cognition training program	SCR (GilSanz, 2009)	SCR		Gil Sanz D, Diego Lorenzo M, Bengochea Seco R, Arrieta Rodríguez M, Lastra Martínez I, Sánchez Calleja R, Alvarez Soltero A. (2009). Efficacy of a social	

Trial reference (author, year)	Original Intervention Name	Distinct Intervention Name	Macro-family intervention 1	Macro-family intervention 2	Manuals references	Type of Adaptation (content, duration/sessions, cultural, unknown)
					cognition training program for schizophrenic patients: A Pilot Study. Span J Psychol. 12(1):184–91.	
Gohar, 2013	Social cognitive skills training	SCR (adapted from Horan, 2012)	SCR		Horan WP, Dolinsky M, Bender A, Green M. [unpublished]. Augmenting Social Cognitive Intervention for Veterans with Schizophrenia (ASCI) Treatment Manual.	Structure, Cultural
Gohar, 2013	Control skills training program	CST (adapted from Wallace, 1992)	SCR		Wallace CJ, Liberman RP, MacKain SJ, Blackwell G, Eckman TA. (1992). Effectiveness and replicability of modules for teaching social and instrumental skills to the severely mentally ill. Am. J. Psychiatry. 149, 654–658.	Content
Gumley, 2003	Cognitive behavioral therapy	CBT (Gumley, 2003)	CBT		Gumley AI, Power KG. (2003) [unpublished]. Targeting Cognitive Behaviour Therapy for relapse: A treatment protocol.	
Guo, 2010	Psychosocial intervention	Psychosocial intervention (Guo, 2007)	PSYSOC		Guo X, Zhao J, Liu Z, Zhai J, Xue Z, Chen J. (2007). Antipsychotic Combination with Psychosocial Intervention on Outcome of Schizophrenia (ACPIOS): Rationale and Design of the Clinical Trial. Clinical Schizophrenia & Related Psychoses. 1(2), 185-192.	
Haddock, 1999	Cognitive behavioral therapy	CBT (Haddock, 1999a, unpublished)	CBT		Haddock G. et al. (1999) [unpublished]. Short-term individual CBT for psychosis.	
Haddock, 1999	Psychoeducation /supportive counselling	Supportive counselling (Haddock, 1999a, unpublished)	PSED		Haddock G. et al. (1999) [unpublished]. Short-term individual CBT for psychosis.	
Hayes, 1995	Social skills training	SST (Hayes, 1995)	NSDG		Hayes RL, Halford WK, Varghese FT. (1995). Social skills training with chronic schizophrenic patients: Effects on negative symptoms and community functioning. Behav Ther. 26:433–49.	
Hayes, 1995	Discussion group	Non-specific discussion group (Brammer, 1973)	SST		Brammer LB. (1973). The helping relationship: Process and Skills. Englewood Cliffs, N J: Prentice-Hall.	
Jenner, 2004	Hallucinations focused integrative treatment	HIT (Jenner, 1996)	HIT		Jenner JA, van Gorcum I, Wiersma D. (1996). "Coping training en cognitieve therapie bij schizofrenie-patienten met chronische en invaliderende hallucinaties: Protocol voor behandeling." Groningen, The Netherlands: Academisch Ziekenhuis en Rijksuniversiteit Groningen, Afdeling en Vakgroep Psychiatrie.	
Jørgensen, 2015	Guided self-determination	Guided self-determination (Jorgensen, 2013)	GSD		Jorgensen R. (2013) [unpublished]. Improving insight in individuals diagnosed with schizophrenia - PhD thesis.	
Kang, 2016	Social skills	SST (Liberman, 1993) + Tai Chi	SST		Kang R, Wu Y, Li Z, Jiang J, Gao Q, Yu Y, et al. (2016). Effect of community-based social skills training and Tai-Chi exercise on outcomes in patients with chronic schizophrenial: A randomized, one-year study. Psychopathology. 49:345–55.	
Kantrowitz, 2016	Cognitive Remediation	CR- Posit Science "Brain Fitness" (Fisher, 2009)	CR		Fisher M, Holland C, Merzenich MM, Vinogradov S. (2009). Using neuroplasticity-based auditory training to improve verbal memory in schizophrenia. Am J Psychiatry. 166(7):805–811. doi: 10.1176/appi.ajp.2009.08050757	
Keefe, 2012	Computerized cognitive remediation training	CR- Posit Science "Brain Fitness" (Mahncke, 2006)	CR		Mahncke HW, Bronstone A, Merzenich MM. (2006). Brain plasticity and functional losses in the aged: Scientific bases for a novel intervention. Prog Brain Res. 157:81–109.	
Kim, 2010	EMDR	EMDR (Kim, 2010)	EMDR		Kim, D. (2010) [unpublished]. EMDR.	
Kim, 2010	Progressive muscle relaxation	Progressive muscle relaxation (Bernstein, 1973)	REL		Bernstein DA, Borkovec, TD. (1973). Progressive Relaxation Training. Champaign, IL: Research Press.	
Kuipers, 1997	Cognitive behavioral therapy	CBT (Fowler, 1995)	CBT		Fowler D, Garety PA, Kuipers L (1995). Cognitive Behaviour Therapy for Psychosis: Theory and Practice. Wiley	
Kumar, 2010	Metacognitive training (group)	MCT (Moritz, 2005)	MCT		Moritz, S., Burlon, M., & Woodward, T. S. (2005). Metacognitive training for schizophrenic patients. Hamburg, Germany: VanHam Campus Verlag.	
Leclerc, 2000	Group rehabilitative coping skills module	Coping skills (Leclerc, 1997)	COPE		Leclerc C, Lesage A, Ricard N. (1997). Pertinence du paradigme stress-coping pour l'elaboration d'un modele de la gestion du stress des personnes atteintes de schizophrenie [Relevance of stress-coping paradigm for the development of a stress	

Trial reference (author, year)	Original Intervention Name	Distinct Intervention Name	Macro-family intervention 1	Macro-family intervention 2	Manuals references	Type of Adaptation (content, duration/sessions, cultural, unknown)
					management model for persons with schizophrenia]. Sante Mentale au Quebec, 22(2), 68-91.	
Lee, 2013	Computerized cognitive remediation training	CR- Cog-trainer (Lee, 2008)	CR		Lee WK, Lee WH, Hwang TY. (2008). Cog-Trainer. Maxmedica, Seoul.	
Lewis, 2002	Cognitive behavioral therapy	CBT (Haddock, 1999)	CBT		Haddock G, Morrison AP, Hopkins R, Lewis S, Tarrier N. (1998). Individual cognitive–behavioural interventions in early psychosis. The British Journal of Psychiatry, 172(S33), 101-106.	
Lewis, 2002	Supportive counselling	Supportive counselling (Lewis, 2002, unpublished)	SUP		Lewis S. (2002) [unpublished]. Supportive Counselling.	
Li, 2015	Cognitive behavioral therapy	CBT (adapted from Kingdon & Turkington, 2005)	CBT		Kingdon DG & Turkington D. (2004). Cognitive Therapy of Schizophrenia. Guilford Press: New York.	Cultural
Li, 2015	Supportive therapy	SUP (Winston, 2004)	SUP		Winston A, Rosenthal RN, Pinsker H. (2004). Introduction to Supportive Psychotherapy. American Psychiatric Publishing Incorporated: New York	
Liberman, 2009	Skills training	SST (Liberman, 2009)	SST		Liberman RP, Mueser K, Wallace CJ, Jacobs HE, Eclanan T. (1986). Training skills in the psychiatrically disabled: Leaming coping and competence. Schizophrenia Bulletin. 2:631-647.	Cultural
Lincoln, 2012	Cognitive behavioral therapy	CBT (Lincoln, 2006)	CBT		Lincoln T. (2006). Kognitive Verhaltenstherapie der Schizophrenie. Ein individuenzentrierter Ansatz zur Veränderung von Wahn, Halluzinationen Halluzinationen und Negativsymptomatik [Cognitive behavior therapy for schizophrenia: An individual-centered approach to changing delusions, hallucinations and negative symptoms]. Göttingen, Germany: Hogrefe	
Lindenmayer, 2013	Computerized cognitive remediation + emotion perception intervention	SCR (Baron-Cohen, 2004)	CR		Baron-Cohen S, Golan O, Wheelwright S, Hill JJ. (2004). Mind Reading: The interactive guide to emotions. London, UK: Jessica Kingsley Limited.	
Lindenmayer, 2013	Computerized cognitive remediation training	CR - CogPack v 8.1 (Marker, 1987-2007)	SCR		Marker K. (2003). COGPACK manual version 5.9. Marker software (Ladenburg).	
Lopez-Luengo, 2016	Attentional rehabilitation	CR - RehaCom (Schuhfried, 2003)	CR		Schuhfried G. (2003). RehaCom Version 5. Basic Manual [Internet]. Magdegurg (DE): Hasomed.	
Lukoff, 1986	Social skills training	SST (Wallace, 1982, Liberman, 1984)	HHS		Liberman RP, Falloon I, Aitchison R A. (1984). Multiple family therapy for schizophrenia: A behavioral, problem-solving approach. Psychosocial Rehabilitation Journal, 7(4), 60–77. doi: 10.1037/h0099685	
Lukoff, 1986	Holistic Health Sessions	Holistic Health Sessions (Lukoff, 1986)	SST		Lukoff D, Wallace CJ, Liberman RP, Burke K. (1986). A holistic program for chronic schizophrenic patients. Schizophrenia bulletin, 12(2), 274–282. doi: 10.1093/schbul/12.2.274	
Mazza, 2010	Social cognition remediation	ETIT (Mazza, 2010)	PS		Mazza M. (2010). [unpublished]. Emotion and ToM Imitation Training.	
Mazza, 2010	Problem-solving training	Problem-solving (Fallon &Talbot 1982; Barbieri, 2006)	SCR		Falloon IR & Talbot RE. (1982). Achieving the goals of day treatment. Journal of Nervous and Mental Disease, 170(5), 279–285.	
Montero, 2001	Behavioral family therapy (psychoeducation, communication skills and problem-solving)	Family PSED (adapted from Leff, 1982; Falloon, 1984)	BFT		Falloon IR, Boyd JL, Mcgill CW. (1984). Family Care of Schizophrenia: A Problem Solving Approach to the Treatment of Mental Illness. New York: Guilford Press.	Content
Montero, 2001	Family psychoeducation	Family PSED (adapted from Leff, 1982; Falloon, 1984)	PSED		Falloon IR, Boyd JL, Mcgill CW. (1984). Family Care of Schizophrenia: A Problem Solving Approach to the Treatment of Mental Illness. New York: Guilford Press.	
Moritz, 2011	Metacognitive training (group) & Metacognitive training (individual)	MCT (Moritz, 2005) + MCT + (Moritz, 2010)	CR		Moritz S, Woodward TS, Burlon M (2005). Metacognitive Skills Training for Patients with Schizophrenia (MCT). Manual. VanHam Campus : Hamburg.	
Moritz, 2011	Computerized cognitive remediation training	CR - CogPack v 5.9 (Marker, 2003)	MCT		Marker K. (2003). COGPACK Manual Version 5.9. Marker Software: Ladenburg.	

Trial reference (author, year)	Original Intervention Name	Distinct Intervention Name	Macro-family intervention 1	Macro-family intervention 2	Manuals references	Type of Adaptation (content, duration/sessions, cultural, unknown)
Moritz, 2013	Metacognitive Training	MCT (Moritz, 2005)	MCT		Moritz S, Woodward TS, Burlon M (2005). Metacognitive Skills Training for Patients with Schizophrenia (MCT). Manual. VanHam Campus : Hamburg.	
Moritz, 2013	Cognitive remediation program	CR - CogPack v 5.9 (Marker, 2003)	CR		Marker K (2003). COGPACK Manual Version 5.9. Marker Software: Ladenburg.	
Morrison, 2014	Cognitive therapy based on a specific cognitive model	CT (Kingdon & Turkington 2005; Morrison, 2004)	CBT		Morrison AP, Renton JC, Dunn H, Williams S, Bentall RP. (2004). Cognitive therapy for psychosis: A formulation-based approach. London: Brunner-Routledge.	
Naeem, 2015	Cognitive behavioral therapy for schizophrenia	CBT (Naeem, 2015, adapted from Kingdon & Turkington, 1994)	CBT		Kingdon DG & Turkington D. (1994). Cognitive-behavioral therapy of schizophrenia. Lawrence Erlbaum.	Cultural
Naeem, 2016	Cognitive behavioral therapy for psychosis	CBT (Naeem, 2016)	CBT		Naeem F, Johal R, McKenna C, Rathod S, Ayub M, Lecomte T, Husain N, Kingdon D, Farooq S. (2016). Cognitive Behavior Therapy for psychosis based Guided Self-help (CBTp-GSH) delivered by frontline mental health professionals: Results of a feasibility study. Schizophrenia research, 173(1-2), 69–74. doi: 10.1016/j.schres.2016.03.003	
Ng, 2006	Social skills training	SST (Bellack, 1986)	SST		Bellack AS, Mueser KT, Gingerich S, Agresta J. (1986). Social skills training for schizophrenia: a step-by-step guide.New York: Guilford Press	
Ojeda, 2012	Cognitive remediation	CR (Ojeda, 2010)	CR		Ojeda N, Peña J, Sánchez P, Bengoetxea E. (2010). La rehabilitación neuropsicológica en psicosis II: El programa rehacop. In Ezcurra J, Gutiérrez, M, Gonzalez-Pinto A eds. Esquizofrenia: Sociogénesis, psicogénesis y condicionamiento biológico. Madrid: Aula Médica:471–495.	
Omiya, 2016	Cognitive remediation	CR (Matsui, 2015, adapted from Delahunty 1993)	CR		Delahunty A. & Morice R. (1993). A Manual for Neurocognitive Rehabilitation for Patients with Chronic Schizophrenia: Frontal Executive Program. Albury New South Wales: Department of Health.	
Penn, 2009	Cognitive-behavioral therapy for auditory hallucinations	CBT (Penn, 2009, unpublished)	CBT		Penn DL. [Unpublished]. Group voices manual: Unpublished manual.	
Penn, 2009	Enhanced supportive therapy	SUP (Penn, 2004, unpublished)	SUP		Penn DL (2004) [Unpublished] Supportive Interpersonal Therapy (SIT) for Schizophrenia	
Penn, 2011	Psychoeducation	PSED (Penn, 2006, unpublished)	PSED		Penn DL, Perkins D, Mueser KT, Waldheter EJ, Cather C. (2006). [Unpublished] The Graduated Recovery Intervention Program (GRIP): An Individual Treatment for First Episode Psychosis.	
Peters, 2010	Cognitive behavioral therapy for psychosis	CBT (adapted from Fowler, 1995)	CBT		Fowler D, Garety PA, Kuipers L.(1995). Cognitive Behaviour Therapy for Psychosis: Theory and Practice. Wiley	
Pinto, 1999	Cognitive behavioral therapy + Social skills training	CBT (adapted from Fowler, 1995) + Social Skills Training (Liberman, 1986)	CBT		Pinto (1999) [Unpublished]. Cognitive-behavioral therapy.	
Pinto, 1999	Supportive Therapy	SupT (Pinto, 1999)	SUP		Pinto (1999) [Unpublished]. Supportive therapy.	
Rakitzi, 2016	Integrated psychological therapy	INT-CR (adapted from Roder, 2008)	SCR		Roder V, Brenner HD, Kienzle N. (2008). Integriertes Psychologisches Therapieprogramm bei schizophren Erkrankten. IPT. Beltz.	Content
Rathod, 2013	Cognitive behavioral therapy for psychosis	CBT (Rathod, 2010 adapted from Kingdon & Turkington, 2005)	CBT		Kingdon GD & Turkington D. (2005). Cognitive therapy for schizophrenia. New York: Guilford Press.	Cultural
Rector, 2003	Cognitive behavioral therapy for schizophrenia	CBT (Rector, 2002)	CBT		Rector NA, & Beck AT. (2002). Cognitive therapy for schizophrenia: from conceptualization to intervention. Canadian journal of psychiatry. Revue canadienne de psychiatrie. 47(1), 39–48.	
Roberts, 2014	Social Cognition and Interaction Training	SCR (Roberts, 2014, unpublished)	SCR		Roberts DL, Penn DL, Combs DR. (s/f) [Unpublished]. Social Cognition and Interaction Training (SCIT): Treatment manual. New York: Oxford University Press.	
Rus-Calafell, 2013	Social skills training	SST (Rus-Calafell, 2009, unpublished)	SST		Rus-Calafell M. (2009) [Unpublished]. Entrenament en Habilitats Socials per a persones amb Esquizofrenia. Universitat de Barcelona.	
Sanchez, 2014	Cognitive remediation	CR (Adapted from Ojeda, 2010)	CR		Ojeda N, Peña J, Sánchez P, Bengoetxea E. (2010). La rehabilitación neuropsicológica en psicosis II: El programa rehacop. In Ezcurra J, Gutiérrez, M,	Content

Trial reference (author, year)	Original Intervention Name	Distinct Intervention Name	Macro-family intervention 1	Macro-family intervention 2	Manuals references	Type of Adaptation (content, duration/sessions, cultural, unknown)
					Gonzalez-Pinto A eds. Esquizofrenia: Sociogénesis, psicogénesis y condicionamiento biológico. Madrid: Aula Médica:471–495.	
Schaub, 2016	Cognitive behavioral therapy (coping skills training)	CBT (Schaub, 2016)	CBT		Schaub A, Mueser KT, Werder T Von, Engel R, Möller H, Falkai P. (2016). A randomized controlled trial of group coping-oriented therapy vs supportive therapy in schizophrenia : Results of a 2-Year Follow-up. Schizophr Bull. 42(1):s71-80.	
Schrank, 2016	Positive psychotherapy	PP (Brownell, 2014)	PP		Brownell T, Schrank B, Slade M. (2014). WELLFOCUS PPT: Positive Psychotherapy for Psychosis Intervention Manual, London: Section for Recovery.	
Sensky, 2000	Cognitive behavioral therapy for schizophrenia	CBT (Kingdon & Turkington, 1994)	CBT		Kingdon DG & Turkington D. (1994). Cognitive-behavioral therapy of schizophrenia. Lawrence Erlbaum.	
Shin, 2002	Psychoeducation (group) with individual supportive counselling	PSED (Shin & Lukens, 2002) + SUP (Shin & Lukens, 2002)	PSED	SUP	Shin SK & Lukens EP. (2002) [Unpublished]. Psychoeducation and supportive therapy.	
Shin, 2002	Supportive sessions (individual)	SUP (Shin, 2002)	SUP		Shin SK & Lukens EP. (2002) [Unpublished]. Psychoeducation and supportive therapy manual.	
Startup, 2004	Cognitive behavioral therapy for psychotic disorders	CBT (Fowler, 1995)	CBT		Fowler D, Garety PA, Kuipers L.(1995). Cognitive Behaviour Therapy for Psychosis: Theory and Practice. Wiley	
Tan, 2016	Cognitive remediation	CR (adapted from Delahunty & Motrice, 1996; Wykes, 2007)	CR		Delahunty A, & Morice R. (1996). Rehabilitation of frontal/executive impairments in schizophrenia. The Australian and New Zealand journal of psychiatry, 30(6), 760–767. doi: 10.3109/00048679609065042.	Unknown
Tao, 2015	Cognitive remediation + psychopharmacologic treatment	CR (Tao, 2015)	CR		Tao J, Zeng Q, Liang J, Zhou A, Yin X, Xu A. (2015). Effects of cognitive rehabilitation training on schizophrenia : 2 years of follow-up. Int J Clin Exp Med. 8(9):16089–94.	
Tarrier, 2014	Cognitive behavioral therapy for suicide prevention	CBT (Tarrier, 2013)	CBT		Tarrier N, Gooding P, Pratt D, Kelly J, Awenat Y, & Maxwell J. (2013). Cognitive Behavioural Prevention of Suicide in Psychosis: A treatment manual. Routledge.	
Tas, 2012	Family Social Cognition and Interaction Training	SCR (adapted from Roberts, 2014, unpublished)	SCR		Roberts DL, Penn DL, Combs DR. (s/f) [Unpublished]. Social Cognition and Interaction Training (SCIT): Treatment manual. New York: Oxford University Press.	Cultural
Turkington, 2002	Cognitive behavioral therapy for schizophrenia	CBT (adapted from Kingdon & Turkington, 1994)	CBT		Kingdon DG & Turkington D. (1994). Cognitive - Behavioural Therapy of Schizophrenia. New York: Guilford Publications.	Content, Structure
Valencia, 2007	Psychosocial skills training & Family psychoeducation	SST (Valencia, 2001) + Family PSED (Valencia, 2007, unpublished)	SST	PSED	Valencia M, Rascón ML, Ortega H. (2001). Psychosocial treatment in patients with schizophrenia. In: Ortega H & Valencia M eds. Schizophrenia: Current Views and Perspectives. National Institute of Psychiatry: Mexico City: 399–454.	
Valencia, 2010	Psychosocial skills training & Family psychoeducation	SST (Valencia, 2001) + Family PSED (adapted from Valencia, 2007, unpublished)	SST	PSED	Valencia M, Rascón ML, Ortega H. (2001). Psychosocial treatment in patients with schizophrenia. In: Ortega H & Valencia M eds. Schizophrenia: Current Views and Perspectives. National Institute of Psychiatry: Mexico City: 399–454.	Structure
Valencia, 2012	Psychosocial skills training & Family psychoeducation	SST (Valencia, 2001) + Family PSED (adapted from Valencia, 2007, unpublished)	SST	PSED	Valencia M, Rascón ML, Ortega H. (2001). Psychosocial treatment in patients with schizophrenia. In: Ortega H & Valencia M eds. Schizophrenia: Current Views and Perspectives. National Institute of Psychiatry: Mexico City: 399–454.	Structure
Valencia, 2013	Psychosocial skills training & Family psychoeducation	SST (Valencia, 2001) + Family PSED (adapted from Valencia, 2007, unpublished)	SST	PSED	Valencia M, Rascón ML, Ortega H. (2001). Psychosocial treatment in patients with schizophrenia. In: Ortega H & Valencia M eds. Schizophrenia: Current Views and Perspectives. National Institute of Psychiatry: Mexico City: 399–454.	Structure
Valmaggia, 2005	Cognitive behavioral therapy	CBT (Valmaggia, 2005, unpublished)	CBT		Valmaggia LR. (2005) [Unpublished]. Cognitive-behavioural Therapy.	
Valmaggia, 2005	Supportive counselling	SUP (Lewis, 2002, unpublished)	SUP		Lewis S. (2002) [unpublished]. Supportive Counselling manual	
Velligan, 2015	Cognitive adaptation training	CAT (Velligan, 2015, unpublished)	CBT		Velligan DI. (2015) [Unpublished]. Cognitive adaptation therapy: a manual-driven compensatory strategies and environmental supports.	
Velligan, 2015	Cognitive behavioral therapy for psychosis	CBT (adapted from Kingdon & Turkington, 2005; Granholm 2005)	CBT	CAT	Kingdon DG & Turkington D. (2005). Cognitive therapy for schizophrenia. New York: Guilford Press.	Content

Trial reference (author, year)	Original Intervention Name	Distinct Intervention Name	Macro-family intervention 1	Macro-family intervention 2	Manuals references	Type of Adaptation (content, duration/sessions, cultural, unknown)
Velligan, 2015	Cognitive behavioral therapy for psychosis + Cognitive adaptation training	CBT (Velligan, 2009) + CAT (Velligan, 2009)	CAT		Velligan DI, Turkington D, Tai S. (2009). Multi-modal cognitive therapy for schizophrenia: Addressing cognitive impairment and dysfunctional cognitive schemas. Schizophrenia Bulletin, 35(suppl 1), 345–346	
Veltro, 2011	Problem-solving training	PS (Fallon &Talbot 1982; Barbieri, 2006)	PS		Falloon IR & Talbot RE. (1982). Achieving the goals of day treatment. Journal of Nervous and Mental Disease, 170(5), 279–285.	
Veltro, 2011	Cognitive-emotional rehabilitation	SCR (Veltro, 2011, unpublished)	SCR		Vendittelli N, Veltro F (2006) [Unpublished]. Teoria della Mente e Schizofrenia: verso una teoria della cura. Decrizione di un nuovo intervento strutturato denominato “Riabilitazione Emozionale Cognitiva”. Psichiatria di Comunità. 4: 169-79.	
Vita, 2011a	Integrated psychological therapy	IntPT (Brenner, 1997 adapted from Brenner, 1994)	CR		Brenner H, Roder V, Hodel B, Kienzie N, Reed D, Liberman R. (1994). Integrated psychological therapy for schizophrenic patients. Seattle: Hogrefe & Huber.	Cultural
Vita, 2011a	Computerized cognitive remediation training	CR - CogPack v 5.9 (Marker, 2003)	SCR		Marker K. (2003). COGPACK Manual Version 5.9. Marker Software: Ladenburg.	
Vita, 2011b	Integrated psychological therapy	IntPT (Brenner, 1997 adapted from Brenner, 1994)	SCR		Brenner H, Roder V, Hodel B, Kienzie N, Reed D, Liberman R. (1994). Integrated psychological therapy for schizophrenic patients. Seattle: Hogrefe & Huber.	Cultural
Wang, 2016	Mindfulness-based psychoeducation	MF-based PSED (Chien, 2013)	MF-PSED		Chan SW, Yip B, Tso S, Cheng B, Tam W. (2009). Evaluation of a psychoeducation program for Chinese clients with schizophrenia and their family caregivers. Patient Educ Couns. 75:67–76. doi: 10.1016/j.pec.2008.08.028	
Wang, 2016	Psychoeducation	PSED (adapted from Chan, 2009)	PSED		Chien WT, Lee IY. (2013). The mindfulness-based psychoeducation program for Chinese patients with schizophrenia. Psychiatr Serv. 64(4), 376–379. doi: 10.1176/appi.ps.002092012.	
Wolwer, 2011	Training of affect recognition	SCR (Frommann, 2003)	CR		Frommann N, Streit M, & Wölwer W. (2003). Remediation of facial affect recognition impairments in patients with schizophrenia: a new training program. Psychiatry research, 117(3), 281–284. doi: 10.1016/s0165-1781(03)00039-8	
Wolwer, 2011	Computerized and non-computerized cognitive remediation training	CR - CogPack v 5.9 (Wolwer, 2011, supplemented Marker, 2003)	SCR		Marker K (2003). COGPACK Manual Version 5.9. Marker Software: Ladenburg.	Content
Wykes, 1999	Cognitive remediation	CR (Delahunty & Motrice, 1993)	CR		Delahunty A & Morice RA. (1993). Training Programme for the Remediation of Cognitive Deficits in Schizophrenia. Albury, New South Wales: Department of Health.	
Wykes, 1999	Psychological based occupational therapy	P-OT (Wykes, 1999, unpublished)	P-OT		Wykes T. (1999) [unpublished]. Intensive Occupational Therapy manual.	
Wykes, 2007	Cognitive remediation	CR (Delahunty & Motrice, 1993; Delahunty, 2002)	CR		Delahunty A & Morice RA. (1993). Training Programme for the Remediation of Cognitive Deficits in Schizophrenia. Albury, New South Wales: Department of Health.	

List of acronyms for interventions and families: BFT: Behavioral family therapy; CAT: Cognitive analytic therapy; CBT: Cognitive-behavioral therapy; COPE: Coping skills; CR: Cognitive remediation; EMDR: Eye movement desensitization and reprocessing; FT: Family therapy; GSD: Guided self-determination; HHS: Holistic health sessions; HIT: Hallucinations focused integrative treatment; ILLMNG: Illness management; INT-CR: Integrated psychological therapy. MCT: Metacognitive training; MF-PSED: Mindfulness-based psychoeducation; MFT: Multifamily treatment; NSDG: Non-specific discussion group; P-OT: Psychological based occupational therapy; PP: Positive psychotherapy; PS: Problem Solving; PSED: Psychoeducation; PSYSOC: Psychosocial intervention; REL: Relaxation; SCR: Social cognitive remediation; SST: Social skills training; SUP: Supportive therapy.

Table S10. Total psychological intervention arms with macro-family and adaptations – Substance use disorders (stimulant use disorders)

Trial reference (author, year)	Original Intervention Name	Distinct Intervention Name	Macro-family intervention 1	Macro-family intervention 2	Manuals references	Type of Adaptation (content, duration/sessions, cultural, unknown)
Carroll, 1994	Cognitive behavioral coping skills training + PHA	CBCST (Carroll, 1997, unpublished)	CBT		Carroll KM (1997) [unpublished]. Cognitive-Behavioral Coping Skills Treatment for Cocaine Dependence. Yale University Psychotherapy Development Center Training Series No. 2. Rockville: National Institute on Drug Abuse. New Haven, Connecticut.	
Carroll, 1994	Clinical management + PHA	ICM (adapted from Fawcett, 1987)	CLIN		Fawcett J, Epstein P, Fiester SJ, Elkin I, Autry JH (1987). Clinical management- imipramine/placebo administration manual: NIMH Treatment of Depression Collaborative Research Program. Psychopharmacol Bull; 23; 309-324.	Content
Carroll, 1994	Cognitive behavioral coping skills training	CBCST (Carroll, 1997, unpublished)	CBT		Carroll KM (1997) [unpublished]. Cognitive-Behavioral Coping Skills Treatment for Cocaine Dependence. Yale University Psychotherapy Development Center Training Series No. 2. Rockville: National Institute on Drug Abuse. New Haven, Connecticut.	
Carroll, 1994	Clinical management	ICM (adapted from Fawcett, 1987)	CLIN		Fawcett J, Epstein P, Fiester SJ, Elkin I, Autry JH (1987). Clinical management- imipramine/placebo administration manual: NIMH Treatment of Depression Collaborative Research Program. Psychopharmacol Bull; 23; 309-324.	Content
Carroll, 1998	Cognitive behavioral coping skills training + PHA	CBCST (Carroll, 1997, unpublished)	CBT		Carroll KM (1997) [unpublished]. Cognitive-Behavioral Coping Skills Treatment for Cocaine Dependence. Yale University Psychotherapy Development Center Training Series No. 2. Rockville: National Institute on Drug Abuse. New Haven, Connecticut.	
Carroll, 1998	Twelve-Step facilitation + PHA	TSF (Baker, 1998, unpublished)	TSF		Baker SM (1998) [unpublished]. Twelve Step Facilitation Therapy for Drug Abuse and Dependence. Yale University Psychotherapy Development Center Training Series No. 3. Rockville: National Institute on Drug Abuse. New Haven, Connecticut.	
Carroll, 1998	Clinical management + PHA	ICM (adapted from Fawcett, 1987)	CLIN		Fawcett J, Epstein P, Fiester SJ, Elkin I, Autry JH (1987). Clinical management- imipramine/placebo administration manual: NIMH Treatment of Depression Collaborative Research Program. Psychopharmacol Bull; 23; 309-324.	Content
Carroll, 1998	Cognitive behavioral coping skills training	CBCST (Carroll, 1997, unpublished)	CBT		Carroll KM (1997) [unpublished]. Cognitive-Behavioral Coping Skills Treatment for Cocaine Dependence. Yale University Psychotherapy Development Center Training Series No. 2. Rockville: National Institute on Drug Abuse. New Haven, Connecticut.	

Trial reference (author, year)	Original Intervention Name	Distinct Intervention Name	Macro-family intervention 1	Macro-family intervention 2	Manuals references	Type of Adaptation (content, duration/sessions, cultural, unknown)
Carroll, 1998	Twelve-Step facilitation	TSF (Baker, 1998, unpublished)	TSF		Baker SM (1998) [unpublished]. Twelve Step Facilitation Therapy for Drug Abuse and Dependence. Yale University Psychotherapy Development Center Training Series No. 3. Rockville: National Institute on Drug Abuse. New Haven, Connecticut.	
Carroll, 2012	Twelve-Step facilitation + PHA	TSF (Baker, 1998, unpublished)	TSF		Baker SM (1998) [unpublished]. Twelve Step Facilitation Therapy for Drug Abuse and Dependence. Yale University Psychotherapy Development Center Training Series No. 3. Rockville: National Institute on Drug Abuse. New Haven, Connecticut.	
Carroll, 2012	Twelve-Step facilitation	TSF (Baker, 1998, unpublished)	TSF		Baker SM (1998) [unpublished]. Twelve Step Facilitation Therapy for Drug Abuse and Dependence. Yale University Psychotherapy Development Center Training Series No. 3. Rockville: National Institute on Drug Abuse. New Haven, Connecticut.	
Carroll, 2014	Computer-based Cognitive behavioral therapy	CBT4CBT (Carroll, 2014)	CBT		Carroll KM, Kiluk BD, Nich C, Gordon MA, Portnoy GA, Marino DR, Ball SA (2014). Computer-assisted delivery of cognitive-behavioral therapy: efficacy and durability of CBT4CBT among cocaine-dependent individuals maintained on methadone. Am J Psychiatry; 171(4):436–44.	
Carroll, 2016	Cognitive behavioral therapy + Contingency management	CBCST (Carroll, 1997, unpublished) + CM (Carroll, 2016, based on Petry, 2000) + PHA	CBT	CM	Carroll KM (1997) [unpublished]. Cognitive-Behavioral Coping Skills Treatment for Cocaine Dependence. Yale University Psychotherapy Development Center Training Series No. 2. Rockville: National Institute on Drug Abuse. New Haven, Connecticut.<br>Carroll KM, Nich C, Petry NM, Eagan DA, Shi JM, Ball SA (2016). A randomized factorial trial of disulfiram and contingency management to enhance cognitive behavioral therapy for cocaine dependence. Drug Alcohol Depend;160:135-142. doi: 10.1016/j.drugalcdep.2015.12.036.<br>Petry NM (2000). A comprehensive guide to the application of contigency management procedures in clinical settings. Drug Alcohol Depend. 58, 9–25	
Carroll, 2016	Cognitive behavioral therapy + PHA	CBCST (Carroll, 1997, unpublished)	CBT		Carroll KM (1997) [unpublished]. Cognitive-Behavioral Coping Skills Treatment for Cocaine Dependence. Yale University Psychotherapy Development Center Training Series No. 2. Rockville: National Institute on Drug Abuse. New Haven, Connecticut.	
Carroll, 2016	Cognitive Behavioral therapy + Contingency management	CBCST (Carroll, 1997, unpublished) + CM (Carroll, 2016, based on Petry, 2000)	CBT	CM	Carroll KM (1997) [unpublished]. Cognitive-Behavioral Coping Skills Treatment for Cocaine Dependence. Yale University Psychotherapy Development Center Training Series No. 2. Rockville: National Institute on Drug Abuse. New Haven,	

Trial reference (author, year)	Original Intervention Name	Distinct Intervention Name	Macro-family intervention 1	Macro-family intervention 2	Manuals references	Type of Adaptation (content, duration/sessions, cultural, unknown)
					Connecticut.<br>Carroll KM, Nich C, Petry NM, Eagan DA, Shi JM, Ball SA (2016). A randomized factorial trial of disulfiram and contingency management to enhance cognitive behavioral therapy for cocaine dependence. Drug Alcohol Depend;160:135-142. doi: 10.1016/j.drugalcdep.2015.12.036.<br>Petry NM (2000). A comprehensive guide to the application of contigency management procedures in clinical settings. Drug Alcohol Depend. 58, 9–25	
Carroll, 2016	Cognitive behavioral therapy	CBCST (Carroll, 1997, unpublished)	CBT		Carroll KM (1997) [unpublished]. Cognitive-Behavioral Coping Skills Treatment for Cocaine Dependence. Yale University Psychotherapy Development Center Training Series No. 2. Rockville: National Institute on Drug Abuse. New Haven, Connecticut.	
Chen, 2013	Integrative mind-body meditation and ear acupunture	IMEA (Chen, 2013, unpublished)	MBT		Chen KW (2013) [unpublished]. Integrative Mind-Body Meditation and Ear Accupunture (IMEA). Treatment manual.	
Crits-Cristoph, 1999	Cognitive therapy + Group Drug Counseling	CT/CBT (Beck, 1993) + GDC (Daley, 2002)	CBT	DC	Beck AT,Wright FD, Newman CF, Liese BS (1993). Cognitive Therapy of Substance Abuse. New York, NY: Guilford Press.<br>Daley DC, Mercer D, Carpenter C (2002). Drug Counseling for Cocaine Addiction: The Collaborative Cocaine Treatment Study Model. National Institute on Drug Abuse. Bethesda, Maryland.	
Crits-Cristoph, 1999	Individual Drug Counseling + Group drug counseling	IDC (Mercer & Woody, 1999) + GDC (Daley, 2002)	DC	DC	Mercer DC, & Woody GE (1999). Individual Drug Counseling. National Institute on Drug Abuse Division of Clinical and Services Research. Rockville, Maryland.<br>Daley DC, Mercer D, Carpenter C (2002). Drug Counseling for Cocaine Addiction: The Collaborative Cocaine Treatment Study Model. National Institute on Drug Abuse. Bethesda, Maryland.	
Crits-Cristoph, 1999	Group Drug Counseling	GDC (Daley, 2002)	DC		Daley DC, Mercer D, Carpenter C (2002). Drug Counseling for Cocaine Addiction: The Collaborative Cocaine Treatment Study Model. National Institute on Drug Abuse. Bethesda, Maryland.	
Crits-Cristoph, 1999	Supportive-Expressive therapy + Group drug counseling	SEPT (Mark & Faude, 1995, unpublished) + GDC (Mercer, 1994)	SUP	DC	Mark D, & Faude J (1995) [unpublished]. Supportive-Expressive Therapy of Cocaine Abuse.<br>Daley DC, Mercer D, Carpenter C (2002). Drug Counseling for Cocaine Addiction: The Collaborative Cocaine Treatment Study Model. National Institute on Drug Abuse. Bethesda, Maryland.	
Donovan, 2013	Stimulant Abuser Groups to Engage in 12-Step	STAGE-12 (Donovan, 2008)	TSF		Donovan DM, Daley D, Perl H, Brigham G, Hodgkins C (2008). Stimulant Abuser Groups to Engage in 12-Step (STAGE-12): Evaluation of a Combined Individual-Group Intervention to Reduce	

Trial reference (author, year)	Original Intervention Name	Distinct Intervention Name	Macro-family intervention 1	Macro-family intervention 2	Manuals references	Type of Adaptation (content, duration/sessions, cultural, unknown)
					Stimulant and Other Drug Use by Increasing 12-Step Involvement. NIDA CTN Protocol 0031.	
Dursteler-MacFarland, 2013	Cognitive-Behavioral Group therapy + PHA	CBGT (Carroll, 1998)	CBT		Carroll KM (1998). A Cognitive-Behavioral Approach: Treating Cocaine Addiction. Rockville, Maryland: NIDA.	
Dursteler-MacFarland, 2013	Cognitive-Behavioral Group therapy	CBGT (Carroll, 1998)	CBT		Carroll KM (1998). A Cognitive-Behavioral Approach: Treating Cocaine Addiction. Rockville, Maryland: NIDA.	
Epstein, 2003	Cognitive behavioral therapy + Contingency management	CBT (Hawkins, 1997, unpublished) + CM (Silverman, 1996)	CBT	CM	Hawkins W, Latkin C, Lewis F, Covi L, Ruckel S (1997) [unpublished].Coping with Life and Recovery: A Skills Training Approach. Treatment Manual.<br>Silverman K, Higgins ST, Brooner RK, Montoya ID, Cone EJ, Schuster CR, Preston KL (1996). Sustained cocaine abstinence in methadone maintenance patients through voucher-based reinforcement therapy. Archives of General Psychiatry;53:409–415.	
Epstein, 2003	Cognitive behavioral therapy + Non-contingent reward	CBT (Hawkins, 1997, unpublished) + NCR	CBT		Hawkins W, Latkin C, Lewis F, Covi L, Ruckel S (1997) [unpublished]. Coping with Life and Recovery: A Skills Training Approach. Treatment Manual.	
Epstein, 2003	Contingency management	CM (adapted from Higgins, 1991, 1993, 1994)	CM		Silverman K, Higgins ST, Brooner RK, Montoya ID, Cone EJ, Schuster CR, Preston KL (1996). Sustained cocaine abstinence in methadone maintenance patients through voucher-based reinforcement therapy. Archives of General Psychiatry;53:409–415. [PubMed: 8624184]	Unknown
Epstein, 2003	Social Support + NCR	SSI (Epstein, 2013, no manual) + NCR	SUP		Not manualized	
Festinger, 2014	Cash-Based Reinforcement therapy	CM (Festinger, 2008, unpublished)	CM		Festinger DS (2008) [unpublished]. Contingency Management for Cocaine Dependance: Cash vs. Vouchers.	
Festinger, 2014	voucher-based reinforcement therapy	CM (Festinger, 2008, unpublished)	CM		Festinger DS (2008) [unpublished]. Contingency Management for Cocaine Dependance: Cash vs. Vouchers.	
Garcia-Fernandez, 2011	Community Reinforcement Approach	CRA (adapted from Budney & Higgings, 1998)	CRA		Budney AJ, & Higgins ST (1998). A Community Reinforcement Plus Vouchers Approach: Treating Cocaine Addiction. Rockville: National Institute on Drug Abuse.	Delivery
Garcia-Fernandez, 2011	Community reinforcement approach + Contingency management	CRA (adapted from Budney & Higgings, 1998) + CM (Budney & Higgings, 1998)	CRA	CM	Budney AJ, & Higgins ST (1998). A Community Reinforcement Plus Vouchers Approach: Treating Cocaine Addiction. Rockville: National Institute on Drug Abuse.	Delivery

Trial reference (author, year)	Original Intervention Name	Distinct Intervention Name	Macro-family intervention 1	Macro-family intervention 2	Manuals references	Type of Adaptation (content, duration/sessions, cultural, unknown)
Garcia-Rodriguez, 2007	Community reinforcement approach + Contingency management (version 1)	CRA (adapted from Budney & Higgings, 1998) + CM (Budney & Higgings, 1998)	CRA	CM	Budney AJ, & Higgins ST (1998). A Community Reinforcement Plus Vouchers Approach: Treating Cocaine Addiction. Rockville: National Institute on Drug Abuse.	Delivery
Garcia-Rodriguez, 2007	Community reinforcement approach + Contingency management (version 2)	CRA (adapted from Budney & Higgings, 1998) + CM (Budney & Higgings, 1998)	CRA	CM	Budney AJ, & Higgins ST (1998). A Community Reinforcement Plus Vouchers Approach: Treating Cocaine Addiction. Rockville: National Institute on Drug Abuse.	Delivery
Ghitza, 2007	manual CM	CM (Vahabzadeh, 2007)	CM		Vahabzadeh M, Lin JL, Epstein DH, Mezghanni M, Schmittner J, Preston KL (2007). Computerized contingency management for motivating behavior change: Automated tracking and dynamic reward reinforcement management. Proceedings of the 20th IEEE International Symposium on Computer-Based Medical Systems (CBMS 2007), 85–90.	
Ghitza, 2007	Computerized standard-density contingency management	CM (Vahabzadeh, 2007)	CM		Vahabzadeh M, Lin JL, Epstein DH, Mezghanni M, Schmittner J, Preston KL (2007). Computerized contingency management for motivating behavior change: Automated tracking and dynamic reward reinforcement management. Proceedings of the 20th IEEE International Symposium on Computer-Based Medical Systems (CBMS 2007), 85–90.	
Ghitza, 2007	Computerized high-density contingency management	CM (Vahabzadeh, 2007)	CM		Vahabzadeh M, Lin JL, Epstein DH, Mezghanni M, Schmittner J, Preston KL (2007). Computerized contingency management for motivating behavior change: Automated tracking and dynamic reward reinforcement management. Proceedings of the 20th IEEE International Symposium on Computer-Based Medical Systems (CBMS 2007), 85–90.	
Hagedorn, 2013	Contingency management	CM (Hagedorn, 2008, unpublished)	CM		Hagerdon H (2008) [unpublished]. Reinforcing Early Abstinence and Treatment Participation (REAP) Study Manual of Operation Reinforcing Early Abstinence and Treatment Participation (REAP) Study Manual of Operations.	
Higgins, 1993	Community reinforcement approach counselling + Contingency management	CRA (Sisson & Azrin, 1989) + CM (Higgings, 1993, 1994)	CRA	CM	Sisson RW, & Azrin NH (1989). The Community Reinforcement Approach. In: Hester RK, Miller WR, eds. Handbook of Alcoholism Treatment Approaches: Effective Alternatives. New York, NY: Pergamon Press Inc; 242-258.<br>Higgins ST, Budney AJ, Bickel WK, Hughes JR, Foerg F, Badger G (1993). Achieving cocaine abstinence with a behavioral approach. Am J Psychiatry;150(5):763-769.	

Trial reference (author, year)	Original Intervention Name	Distinct Intervention Name	Macro-family intervention 1	Macro-family intervention 2	Manuals references	Type of Adaptation (content, duration/sessions, cultural, unknown)
Higgins, 1993	Twelve-Step Facilitation Drug Abuse Counselling + Non-contingent reinforcement	TSF (Narcotics Anonymous, 1988) + NCR	TSF		Narcotics Anonymous (1988). Narcotics Anonymous, 5th ed, Van Nuys, Calif, World Service Office.	
Higgins, 1994	Community reinforcement approach + Contingency management	CRA (Sisson & Azrin, 1989) + CM (Higgings, 1993, 1994)	CRA	CM	Sisson RW, & Azrin NH (1989). The Community Reinforcement Approach. In: Hester RK, Miller WR, eds. Handbook of Alcoholism Treatment Approaches: Effective Alternatives. New York, NY: Pergamon Press Inc; 242-258. Higgins ST, Budney AJ, Bickel WK, Foerg FE, Donham R, Badger GJ (1994). Incentives improve outcome in outpatient behavioral treatment of cocaine dependence. Arch Gen Psychiatry;51(7):568-576.	
Higgins, 1994	Community reinforcement approach	CRA (Sisson & Azrin, 1989) + NCR	CRA		Sisson RW, & Azrin NH (1989). The Community Reinforcement Approach. In: Hester RK, Miller WR, eds. Handbook of Alcoholism Treatment Approaches: Effective Alternatives. New York, NY: Pergamon Press Inc; 242-258.	
Higgins, 2000	Community reinforcement approach + Contingency management	CRA (Budney & Higgings, 1998) + CM (Budney & Higgings, 1998)	CRA	CM	Budney AJ, & Higgins ST (1998). A Community Reinforcement Plus Vouchers Approach: Treating Cocaine Addiction. Rockville: National Institute on Drug Abuse.	
Higgins, 2000	Community reinforcement approach + Non-contingent reinforcement	CRA (Budney & Higgings, 1998) + NCR	CRA		Budney AJ, & Higgins ST (1998). A Community Reinforcement Plus Vouchers Approach: Treating Cocaine Addiction. Rockville: National Institute on Drug Abuse.	
Higgins, 2003	Community reinforcement approach + Contingency management	CRA (Budney & Higgings, 1998) + CM (Budney & Higgings, 1998)	CRA	CM	Budney AJ, & Higgins ST (1998). A Community Reinforcement Plus Vouchers Approach: Treating Cocaine Addiction. Rockville: National Institute on Drug Abuse.	
Higgins, 2003	Contingency management	CM (adapted from Higgins, 1991, 1993, 1994)	CM		Budney AJ, & Higgins ST (1998). A Community Reinforcement Plus Vouchers Approach: Treating Cocaine Addiction. Rockville: National Institute on Drug Abuse.	
Kirby, 1998	Cognitive-behavioral counselling + Contingency management	CBC (Bux, 1998; Platt, 1991) + CM (Stitzer, 1992)	CBT	CM	Bux DA, Lamb RJ, Platt JJ, Husband SD, Kirby KC (1992). 26-session cognitive-behavioral treatment program for co-caine and stimulant abuse.In Jerome J. Platt, AlleghenyUniversity of the Health Sciences, Division of Addiction Research andTreatment, Mailstop 984, Broad and Vine Streets, Philadelphia, PA 19102-1192. Platt JJ, McKim PJ, Husband SD (1991). Training in interpersonal problem solving trainer's manual. In Jerome J. Platt, Allegheny University of the Health Sciences, Division of Addic-tion Research and Treatment, Mailstop 984, Broad and Vine Streets, Philadelphia, PA 19102-1192.	

**Trial reference (author, year)**	**Original Intervention Name**	**Distinct Intervention Name**	**Macro-family intervention 1**	**Macro-family intervention 2**	**Manuals references**	**Type of Adaptation (content, duration/sessions, cultural, unknown)**
					Stitzer ML, Iguchi MY, Felch LJ (1992). Contingent take- home incentive: Effects on drug use of methadone maintenance patients. Journal of Consulting and Clinical Psychology, 60, 927-934.	
Kirby, 1998	Cognitive-behavioral counseling	CBC (Bux, 1998; Platt, 1991)	CBT		Bux DA, Lamb RJ, Platt JJ, Husband SD, Kirby KC (1992). 26-session cognitive-behavioral treatment program for co-caine and stimulant abuse.In Jerome J. Platt, AlleghenyUniversity of the Health Sciences, Division of Addiction Research andTreatment, Mailstop 984, Broad and Vine Streets, Philadelphia, PA 19102-1192.<br>Platt JJ, McKim PJ, Husband SD (1991). Training in interpersonal problem solving trainer's manual. In Jerome J. Platt, Allegheny University of the Health Sciences, Division of Addic-tion Research and Treatment, Mailstop 984, Broad and Vine Streets, Philadelphia, PA 19102-1192.	
Landovitz, 2015	Contingency management	CM (Reback, 2010, unpublished)	CM		Reback CJ, Landovitz RJ, Shoptav S, Lake J (2010) [unpublished]. Optimizing Access to Non-occupational Post Exposure Prophylaxis for HIV Using Contingency Management in Stimulant-Using Men Who Have Sex with Men.	
Ledgerwood, 2006	Contingency management	CM (Petry, 2000)	CM		Petry NM (2000). A comprehensive guide to the application of contingency management procedures in general clinic settings. Drug Alcohol Depend. 58, 9–25.	
Maude-Griffin, 1998	Cognitive-behavioral therapy	CT/CBT (adapted from Beck, 1993)	CBT		Beck AT, Wright FD, Newman CF, Liese BS (1993). Cognitive therapy of substance abuse. New York: Guilford Press .	Unknown
Maude-Griffin, 1998	12 steps of Alcoholics Anonymous and Cocaine Anonymous	TSF (adapted from Nowinski & Baker, 1992)	TSF		Nowinski I, & Baker S (1992). The twelve-step facilitation handbook: A systematic approach to early recovery from alcoholism and addiction. New York: Lexington Books.	Unknown
McDonnell, 2013	Contingency management	CM (Peirce, 2006)	CM		Peirce JM, Petry NM, Stitzer ML, Blaine J, Kellogg S, Satterfield F, Schwartz M, Krasnansky J, Pencer E, Silva-Vazquez L, Kirby KC, Royer-Malvestuto C, Roll JM, Cohen A, Copersino ML, Kolodner K, Li R (2006). Effects of lower-cost incentives on stimulant abstinence in methadone maintenance treatment: a National Drug Abuse Treatment Clinical Trials Network study. Arch Gen Psychiatry; 63:201–20.	
McKay, 1997	Individualized relapse prevention (RP) aftercare	RP aftercare (McKay & Feeley, 1993, unpublished)	CBT		McKay J, & Feeley M (1993) [unpublished]. Relapse Prevention Manual.	
Menza, 2010	Contingency management	CM (adapted from Shoptaw, 2005)	CM		Shoptaw S, Reback CJ, Peck JA, Yang X, Rotheram-Fuller E, Larkins S Veniegas RC, Freese TE, Hucks-Ortiz C (2005).	Unknown

Trial reference (author, year)	Original Intervention Name	Distinct Intervention Name	Macro-family intervention 1	Macro-family intervention 2	Manuals references	Type of Adaptation (content, duration/sessions, cultural, unknown)
					Behavioral treatment approaches for methamphetamine dependence and HIV-related sexual risk behaviors among urban gay and bisexual men. Drug Alcohol Depend, 78(2):125-134.	
Miguel, 2016	Contingency management	CM (Miguel, 2016, unpublished)	CM		Miguel M (2016) [unpublished]. Effectiveness of Contingency Management in the Treatment of Crack Addiction for Individuals Living in the “Crackland” Region. A Single-Blind Randomized Controlled Trial.	
Milby, 2008	Contingency management + (contingency management + behavioral day treatment)	CM+BDT (Milby, 2008, no manual)	CM	CBT	Not manualized	
Milby, 2008	Contingency management	CM (Milby, 2008, no manual)	CM		Not manualized	
Peirce, 2006	Contingency management	CM (Peirce, 2006)	CM		Peirce JM, Petry NM, Stitzer ML, Blaine J, Kellogg S, Satterfield F, Schwartz M, Krasnansky J, Pencer E, Silva-Vazquez L, Kirby KC, Royer-Malvestuto C, Roll JM, Cohen A, Copersino ML, Kolodner K, Li R (2006). Effects of lower-cost incentives on stimulant abstinence in methadone maintenance treatment: a National Drug Abuse Treatment Clinical Trials Network study. Arch Gen Psychiatry; 63:201–20.	
Petitjean, 2014	Cognitive-behavioral therapy + Contingency management	CBT (adapted from Carroll, 1998) + CM (Petry 2000)	CBT	CM	Carroll KM (1998). A Cognitive-Behavioral Approach: Treating Cocaine Addiction. U.S. Department of Health and Human Services, National Institute of Health, Maryland. Dürsteler-MacFarland KM, Schmid O, Strasser H, Wiesbeck GA (2010). Therapie manual Kokaina bhängigkeit: Grundlagen und Arbeitsmaterialien zur kognitiv-verhaltenstherapeutischen Behandlung. Kohlhammer, Stuttgart. Petry NM (2000). A comprehensive guide to the application of contingency management procedures in clinical settings. Drug Alcohol Depend. 58, 9–25.	Unknown
Petitjean, 2014	Cognitive-behavioral therapy	CBT (adapted from Carroll, 1998)	CBT		Carroll KM (1998). A Cognitive-Behavioral Approach: Treating Cocaine Addiction. U.S. Department of Health and Human Services, National Institute of Health, Maryland. Dürsteler-MacFarland KM, Schmid O, Strasser H, Wiesbeck GA (2010). Therapie manual Kokaina bhängigkeit: Grundlagen und Arbeitsmaterialien zur kognitiv-verhaltenstherapeutischen Behandlung. Kohlhammer, Stuttgart.	Unknown

Trial reference (author, year)	Original Intervention Name	Distinct Intervention Name	Macro-family intervention 1	Macro-family intervention 2	Manuals references	Type of Adaptation (content, duration/sessions, cultural, unknown)
Petry, 2002	Contingency management	CM (adapted from Petry, 2000)	CM		Petry NM (2000). A comprehensive guide to the application of contingency management procedures in general clinic settings. Drug Alcohol Depend. 58, 9–25.	Content
Petry, 2005a	Abstinence-based contingency management	CM (adapted from Petry, 2000)	CM		Petry NM (2000). A comprehensive guide to the application of contingency management procedures in general clinic settings. Drug Alcohol Depend. 58, 9–25.	Content
Petry, 2005b	Contingency management	CM (Petry, 2001)	CM		Petry NM, Martin B, Finocche C (2001). Contingency management in group treatment: A demonstration project in an HIV drop-in center. Journal ofSubstance Abuse Treatment, 21, 89–96.	
Petry, 2007	Contingency management	CM (adapted from Petry, 2000)	CM		Petry NM (2000). A comprehensive guide to the application of contingency management procedures in general clinic settings. Drug Alcohol Depend. 58, 9–25.	Content
Petry, 2007	Contingency management	CM (adapted from Petry, 2000)	CM		Petry NM (2000). A comprehensive guide to the application of contingency management procedures in general clinic settings. Drug Alcohol Depend. 58, 9–25.	Content
Petry, 2012a	Contingency management	CM (Petry & Stitzer 2002)	CM		Petry NM, & Stitzer ML (2002). Contingency management: Using motivational incentives to improve drug abuse treatment. West Haven, CT: Yale University Psychotherapy Development Center	
Petry, 2012b	Contingency management	CM (adapted from Petry, 2000)	CM		Petry NM (2000). A comprehensive guide to the application of contingency management procedures in general clinic settings. Drug Alcohol Depend. 58, 9–25.	Content
Petry, 2012b	Contingency management	CM (adapted from Petry, 2000)	CM		Petry NM (2000). A comprehensive guide to the application of contingency management procedures in general clinic settings. Drug Alcohol Depend. 58, 9–25.	Content
Petry, 2012b	Contingency management	CM (adapted from Petry, 2000)	CM		Petry NM (2000). A comprehensive guide to the application of contingency management procedures in general clinic settings. Drug Alcohol Depend. 58, 9–25.	Content
Petry, 2012b	Contingency management	CM (adapted from Petry, 2000)	CM		Petry NM (2000). A comprehensive guide to the application of contingency management procedures in general clinic settings. Drug Alcohol Depend. 58, 9–25.	Content
Petry, 2013	Contingency management	CM (adapted from Petry, 2000)	CM		Petry NM (2000). A comprehensive guide to the application of contingency management procedures in general clinic settings. Drug Alcohol Depend. 58, 9–25.	Content

Trial reference (author, year)	Original Intervention Name	Distinct Intervention Name	Macro-family intervention 1	Macro-family intervention 2	Manuals references	Type of Adaptation (content, duration/sessions, cultural, unknown)
Poling, 2006	Contingency management + PHA	CM (adapted from Petry, 2000)	CM		Petry NM (2000). A comprehensive guide to the application of contingency management procedures in general clinic settings. Drug Alcohol Depend. 58, 9–25.	
Poling, 2006	Contingency management	CM (adapted from Petry, 2000)	CM		Petry NM (2000). A comprehensive guide to the application of contingency management procedures in general clinic settings. Drug Alcohol Depend. 58, 9–25.	
Rawson, 2002	Cognitive-behavioral therapy	CBT (Rawson, 1989, 1995)	CBT		Rawson RA, Obert JL, McCann MJ, Smith DP, Scheffey EH (1989). The Neurobehavioral Treatment Manual. Beverly Hills, Calif: Matrix.	
Rawson, 2002	Contingency management	CM (Higgins, 1991, 1993, 1994)	CM		Higgins T, Budney J, Bickel K, Hughes R, Foerg F, Fenwick W (1991). A behavioral approach to achieving initial cocaine abstinence. AmJPsychiatry;148:1218- 1224.<br>Higgins T, Budney J, Bickel K, Hughes R, Foerg F, Badger J (1993). Achieving cocaine abstinence with a behavioral approach. Am J Psychiatry;150: 763-769.	
Rawson, 2002	Contingency management + Cognitive-behavioral therapy	CBT (Rawson, 1989, 1995) + CM (Higgins, 1991, 1993)	CM	CBT	Rawson RA, Obert JL, McCann MJ, Smith DP, Scheffey EH (1989). The Neurobehavioral Treatment Manual. Beverly Hills, Calif: Matrix.<br>Higgins ST, Delaney DD, Budney AJ, Bickel WK, Hughes JR, Feorg F, Fenwick JW (1991). A behavioral approach to achieving initial cocaine abstinence. Am J Psychiatry. 148:1218-1224.<br>Higgins T, Budney J, Bickel K, Hughes R, Foerg F, Badger J (1993). Achieving cocaine abstinence with a behavioral approach. Am J Psychiatry;150: 763-769.	
Rawson, 2006	Cognitive-behavioral therapy	CBT (Rawson, 1989, 1995)	CBT		Rawson RA, Obert JL, McCann MJ, Smith DP, Scheffey EH (1989). The Neurobehavioral Treatment Manual. Beverly Hills, Calif: Matrix.	
Rawson, 2006	Contingency management	CM (Higgins, 1991, 1993, 1994)	CM		Higgins T, Budney J, Bickel K, Hughes R, Foerg F, Fenwick W (1991). A behavioral approach to achieving initial cocaine abstinence. AmJPsychiatry;148:1218- 1224.<br>Higgins T, Budney J, Bickel K, Hughes R, Foerg F, Badger J (1993). Achieving cocaine abstinence with a behavioral approach. Am J Psychiatry;150: 763-769.	
Rawson, 2006	Contingency management + Cognitive-behavioral therapy	CBT (Rawson, 1989, 1995) + CM (Higgins, 1991, 1993)	CM	CBT	Rawson RA, Obert JL, McCann MJ, Smith DP, Scheffey EH (1989). The Neurobehavioral Treatment Manual. Beverly Hills, Calif: Matrix.<br>Higgins ST, Delaney DD, Budney AJ, Bickel WK, Hughes JR, Feorg F, Fenwick JW (1991). A behavioral approach to achieving	

Trial reference (author, year)	Original Intervention Name	Distinct Intervention Name	Macro-family intervention 1	Macro-family intervention 2	Manuals references	Type of Adaptation (content, duration/sessions, cultural, unknown)
					initial cocaine abstinence. Am J Psychiatry. 148:1218-1224.<br>Higgins T, Budney J, Bickel K, Hughes R, Foerg F, Badger J (1993). Achieving cocaine abstinence with a behavioral approach. Am J Psychiatry;150: 763-769.	
Roll, 2013	Contingency management one-month + Psychosocial treatment	CBT (Rawson, 1995) + CM (adapted from Roll, 1996)	CM	CBT	Rawson RA, Shoptaw SJ, Obert JL, McCann MJ, Hasson AL, Marinelli-Casey PJ, …, Ling W (1995). An intensive outpatient approach for cocaine abuse treatment. The Matrix model. Journal of substance abuse treatment; 12(2):117–127.<br>Roll JM, Higgins ST, Badger GJ (1996). An experimental comparison of three different schedules of reinforcement of drug abstinence using cigarette smoking as an exemplar. Journal of applied behavior analysis. 29(4):495–504.	Content
Roll, 2013	Contingency management two-month + Psychosocial treatment	CBT (Rawson, 1995) + CM (adapted from Roll, 1996)	CM	CBT	Rawson RA, Shoptaw SJ, Obert JL, McCann MJ, Hasson AL, Marinelli-Casey PJ, …, Ling W (1995). An intensive outpatient approach for cocaine abuse treatment. The Matrix model. Journal of substance abuse treatment; 12(2):117–127.<br>Roll JM, Higgins ST, Badger GJ (1996). An experimental comparison of three different schedules of reinforcement of drug abstinence using cigarette smoking as an exemplar. Journal of applied behavior analysis. 29(4):495–504.	Content
Roll, 2013	Contingency management three-month + Psychosocial treatment	CBT (Rawson, 1995) + CM (adapted from Roll, 1996)	CM	CBT	Rawson RA, Shoptaw SJ, Obert JL, McCann MJ, Hasson AL, Marinelli-Casey PJ, …, Ling W (1995). An intensive outpatient approach for cocaine abuse treatment. The Matrix model. Journal of substance abuse treatment; 12(2):117–127.<br>Roll JM, Higgins ST, Badger GJ (1996). An experimental comparison of three different schedules of reinforcement of drug abstinence using cigarette smoking as an exemplar. Journal of applied behavior analysis. 29(4):495–504.	Content
Roll, 2013	Standard psychosocial treatment	CBT (Rawson, 1989, 1995)	CBT		Rawson RA, Shoptaw SJ, Obert JL, McCann MJ, Hasson AL, Marinelli-Casey PJ, …, Ling W (1995). An intensive outpatient approach for cocaine abuse treatment. The Matrix model. Journal of substance abuse treatment; 12(2):117–127.	
Sanchez Hervas, 2010	Community reinforcement approach	CRA (adapted from Budney & Higgins, 1998)	CRA		Budney AJ, & Higgins ST (1998). A community reinforcement plus vouchers approach: treating cocaine addiction. Rockville: National Institute on Drug Abuse.	Content
Schottenfeld, 2011	Community reinforcement approach + Contingency management	CRA (Budney & Higgins, 1998) + CM (Budney & Higgins, 1998)	CRA	CM	Budney AJ, & Higgins ST (1998). A community reinforcement plus vouchers approach: treating cocaine addiction. Rockville: National Institute on Drug Abuse.	

**Trial reference (author, year)**	**Original Intervention Name**	**Distinct Intervention Name**	**Macro-family intervention 1**	**Macro-family intervention 2**	**Manuals references**	**Type of Adaptation (content, duration/sessions, cultural, unknown)**
Schottenfeld, 2011	Twelve-Step facilitation + Contingency management	TSF (Nowinski & Baker, 1992) + CM (Higgins, 1993)	TSF	CM	Nowinski J, Baker S, Carroll KM (1992). Twelve-step Facilitation Therapy Manual: a clinical research guide for therapists treating individuals with alcohol abuse and dependence, NIAAA Project MATCH Monograph Series (Rockville, MD, NIAAA).<br>Higgins ST, Budney AJ, Bickel WK, Hughes JR, Foerg F, Badger G (1993). Achieving cocaine abstinence with a behavioral approach. Am. J. Psychiatry 150, 763–769.	
Schottenfeld, 2011	Community reinforcement approach + Voucher control	CRA (Budney & Higgins 1998) + VC	CRA	CM	Budney AJ, & Higgins ST (1998). A community reinforcement plus vouchers approach: treating cocaine addiction. Rockville: National Institute on Drug Abuse.	
Schottenfeld, 2011	Twelve-Step facilitation + Voucher control	TSF (Nowinski & Baker, 1992) + VC	TSF	CM	Nowinski I, & Baker S (1992). The twelve-step facilitation handbook: A systematic approach to early recovery from alcoholism and addiction. New York: Lexington Books.	
Secades Villa, 2013	Community Reinforcement Approach	CRA (Budney & Higgins, 1998)	CRA		Budney AJ, & Higgins ST (1998). A community reinforcement plus vouchers approach: treating cocaine addiction. Rockville: National Institute on Drug Abuse.	
Secades Villa, 2013	Community reinforcement approach + Contingency management	CRA (Budney & Higgins, 1998) + CM (adapted from Budney & Higgins, 1998)	CRA	CM	Budney AJ, & Higgins ST (1998). A community reinforcement plus vouchers approach: treating cocaine addiction. Rockville: National Institute on Drug Abuse.	Structure
Shoptaw, 2005	Cognitive-behavioral therapy	CBT (Rawson, 1989, 1995)	CBT		Rawson RA, Shoptaw SJ, Obert JL, McCann MJ, Hasson AL, Marinelli-Casey PJ, …, Ling W (1995). An intensive outpatient approach for cocaine abuse treatment. The Matrix model. Journal of substance abuse treatment; 12(2):117–127.	
Shoptaw, 2005	Contingency management	CM (Higgins, 1991, 1993, 1994)	CM		Higgins T, Budney J, Bickel K, Hughes R, Foerg F, Badger J (1993). Achieving cocaine abstinence with a behavioral approach. Am J Psychiatry;150: 763-769.	
Shoptaw, 2005	Cognitive-behavioral therapy + Contingency management	CBT (Rawson, 1989, 1995) + CM (Higgins, 1991, 1993)	CBT	CM	Rawson RA, Shoptaw SJ, Obert JL, McCann MJ, Hasson AL, Marinelli-Casey PJ, …, Ling W (1995). An intensive outpatient approach for cocaine abuse treatment. The Matrix model. Journal of substance abuse treatment; 12(2):117–127.<br>Rawson RA, Obert JL, McCann MJ, Smith DP, Scheffey EH (1989). The Neurobehavioral Treatment Manual. Beverly Hills, Calif: Matrix.<br>Higgins T, Budney J, Bickel K, Hughes R, Foerg F, Badger J (1993). Achieving cocaine abstinence with a behavioral approach. Am J Psychiatry;150: 763-769.	

Trial reference (author, year)	Original Intervention Name	Distinct Intervention Name	Macro-family intervention 1	Macro-family intervention 2	Manuals references	Type of Adaptation (content, duration/sessions, cultural, unknown)
Shoptaw, 2005	Gay-specific cognitive-behavioral therapy	GCBT (Shoptaw, 1998, 2005)	CBT		Shoptaw S, Reback CJ, Freese TE, Rawson RA (1998). Behavioral interventions for methamphetamine abusing gay and bisexual men: A Treatment Manual Combining Relapse Prevention and HIV Risk-Reduction Interventions. Friends Research Institute, Los Angeles, CA.	
Shoptaw, 2008	Gay-specific cognitive-behavioral therapy	GCBT (Shoptaw, 1998, 2005)	CBT		Shoptaw S, Reback CJ, Peck JA, Yang X, Rotheram-Fuller E, Larkins S, ..., Hucks-Ortiz C (2005). Behavioral treatment approaches for methamphetamine dependence and HIV-related sexual risk behaviors among urban gay and bisexual men. Drug Alcohol Depend 78(2):125-134. doi: 10.1016/j.drugalcdep.2004.10.004.	
Shoptaw, 2008	Gay-specific social support therapy	GSST (no manual)	SUP		Not manualized	
Silverman, 1996	Contingency management	CM (Higgins, 1991, 1993, 1994)	CM		Higgins ST, Delaney DD, Budney AJ, Bickel WK, Hughes JR, Feorg F, Fenwick JW (1991). A behavioral approach to achieving initial cocaine abstinence. Am J Psychiatry. 148:1218-1224.	
Silverman, 1998	Contingency management (Escalating reinforcement group)	CM (adapted from Higgins, 1991, 1993, 1994)	CM		Silverman K, Higgins ST, Brooner RK, Montoya ID, Cone EJ, Schuster CR, Preston KL (1996). Sustained cocaine abstinence in methadone maintenance patients through voucher-based reinforcement therapy. Archives of General Psychiatry;53:409–415. Higgins ST, Budney AJ, Bickel WK, Foerg FE, Donham R, Badger MS (1994). Incentives improve outcome in outpatient behavioral treatment of cocaine dependence. Archives of General Psychiatry, 51, 568-576.	Unknown
Silverman, 1998	Contingency management (Escalating reinforcement group + Start-Up bonus)	CM (adapted from Higgins, 1991, 1993, 1994)	CM		Silverman K, Higgins ST, Brooner RK, Montoya ID, Cone EJ, Schuster CR, Preston KL (1996). Sustained cocaine abstinence in methadone maintenance patients through voucher-based reinforcement therapy. Archives of General Psychiatry;53:409–415. Higgins ST, Budney AJ, Bickel WK, Foerg FE, Donham R, Badger MS (1994). Incentives improve outcome in outpatient behavioral treatment of cocaine dependence. Archives of General Psychiatry, 51, 568-576.	Unknown
Smout, 2010	Cognitive-behavioral therapy	CBT (Smout & Minniti, 2004, unpublished)	CBT		Smout MF & Minniti R (2004) [unpublished]. Relapse prevention skills training for the treatment of people with methamphetamine abuse problems. Treatment manual.	

Trial reference (author, year)	Original Intervention Name	Distinct Intervention Name	Macro-family intervention 1	Macro-family intervention 2	Manuals references	Type of Adaptation (content, duration/sessions, cultural, unknown)
Smout, 2010	Acceptance and commitment therapy	ACT (Smout, 2015, unpublished)	CBT		Smout MF (2015) [unpublished]. Acceptance and commitment therapy for the treatment of people with methamphetamine abuse problems.	
Umbricht, 2014	Contingency management + PHA	CM (adapted from Higgins, 1991, 1993, 1994)	CM		Silverman K, Higgins ST, Brooner RK, Montoya ID, Cone EJ, Schuster CR, Preston KL (1996). Sustained cocaine abstinence in methadone maintenance patients through voucher-based reinforcement therapy. Archives of General Psychiatry;53:409–415. [PubMed: 8624184]	Unknown
Umbricht, 2014	Contingency management	CM (adapted from Higgins, 1991, 1993, 1994)	CM		Silverman K, Higgins ST, Brooner RK, Montoya ID, Cone EJ, Schuster CR, Preston KL (1996). Sustained cocaine abstinence in methadone maintenance patients through voucher-based reinforcement therapy. Archives of General Psychiatry;53:409–415. [PubMed: 8624184]	Unknown

List of acronyms for interventions and families: ACT: Acceptance and Commitment Therapy. BDT: Behavioral Day Treatment. CBC: Cognitive-behavioral counseling. CBCST: Cognitive Behavioral Coping Skills Treatment. CBGT: Cognitive-Behavioral Group Therapy. CBT: Cognitive-Behaviour Therapy. CBT4CBT: Computer-Based Cognitive-Behaviour Therapy. CLIN: Clinical Management. CM: Contingency Management. CRA: Community Reinforcement Approach. DC: Drug Counselling. GCBT: Gay-specific Cognitive Behavioral Therapy. GDC: Group Drug Counselling. GSST: Gay-specific social support therapy. ICM: Intensive Clinical Management. IDG: Individual Drug Counselling. IMEA: Integrative Mind-Body Meditation and Ear Accupunture. MBT: Mind-Body Therapy. NCR: Non-Contingent Rewards. RP: Relapse Prevention. SEPT: Supportive-Expressive Psychodynamic Therapy. SSI0Social support intervention. STAGE-12: Specifically, Stimulant Abuser Groups to Engage in 12-Step. SUP: Supportive Therapy. TSF: Twelve-Step facilitation. VC: Voucher Control. PHA: Pharmacotherapy

Tomei G, *et al. BMJ Ment Health* 2026; 29:e302555. doi: 10.1136/bmjment-2026-302555

Table S11. Distinct interventions assigned to multiple families

Disorder	Distinct intervention name	Family intervention 1	Family intervention 2	Family intervention 3	Type of adaptation (content, duration/sessions, cultural, unknown)
Anorexia	Family & psychodynamic (Hall & Crisp, 1987, no manual)	FT	PSYD		
Anorexia	CBT-AN (Pike, 2003, unpublished) + LEAP (Taranis et al., 2011)	CBT	LEAP		
Bipolar	DBT-SK (Adapted from Linehan, 1993b)	DBT	PSED		Content, Structure
Bipolar	FEST (Hautzinger, 2024)	SUP	PSED		
Bipolar	GPI (Castle, 2007)	PSED	CBT		
Bipolar	ICM (Adapted from Fawcett, 1987) + IPSRT (Frank, 2005)	CLIN	IPSRT		Content, Structure
Bipolar	IFPT (Rea, 2003, no manual)	PSED	CBT		
Bipolar	SSCM (Crowe, 2003, unpublished)	CLIN	SUP		
Borderline	AP (Andreoli, 2016, unpublished)	PSYD	CBT		Delivey
Borderline	CCT (Cottraux, 2009, unpublished)	SUP	PSYD		
Borderline	CT (Cottraux, 2009, unpublished)	CBT	SCH		
Borderline	CTBE (Leppanen, 2015)	SCH	DBT		
Borderline	DBT-E (Turner, 2000; adapted from Linehan, 1993a)	DBT	PSYD		Content
Borderline	GET (Visintini, 2020, unpublished)	PSYD	CBT		Content, structure
Borderline	MAAP (Herpertz, 2020, unpublished)	DBT	CBT	PSYD	
Borderline	MACT (Schmidt & Davidson, 2004)	CBT	DBT		Content
Borderline	SUP (Rockland, 1992)	SUP	PSYD		
Bulimia	CBT (Bulik, 1993; unpublished) + ERP (Bulik, 1993; unpublished)	CBT	ERP		Content
Bulimia	CBT (Bulik, 1993; unpublished) + Relaxation (not manualized)	CBT	REL		
Bulimia	CBT (Fairburn & Cooper, 1989) + ERP (Adapted from Rosen & Leitenberg, 1985)	CBT	ERP		Content
Bulimia	CBT (Fairburn, 1993) + Functional analysis (Adapted from Ghaderi, 2007)	CBT	FUN		
Bulimia	CBT (Fairburn, 1985) + ERP (Leitenberg, 1988)	CBT	ERP		Content, Delivery
Bulimia	CBT (Wilson, 1991; unpublished) + ERP (Wilson, 1986)	CBT	ERP		
Schizophrenia and psychotic	CBT (adapted from Kingdon & Turkington, 2005; Granholm 2005)	CBT	CAT		
Schizophrenia and psychotic	PSED (Shin & Lukens, 2002) + SUP (Shin & Lukens, 2002)	PSED	SUP		
Schizophrenia and psychotic	SST (Liberman, 1993) + family PSED (Cai, 2015)	SST	PSED		
Schizophrenia and psychotic	SST (Valencia, 2001) + Family PSED (adapted from Valencia, 2007, unpublished)	SST	PSED		Structure
Substance use disorders	CBCST (Carroll, 1997, unpublished) + CM (Carroll, 2016, based on Petry, 2000) + PHA	CBT	CM		

Disorder	Distinct intervention name	Family intervention 1	Family intervention 2	Family intervention 3	Type of adaptation (content, duration/sessions, cultural, unknown)
Substance use disorders	CBT (Hawkins, 1997, unpublished) + CM (Silverman, 1996)	CBT	CM		
Substance use disorders	CRA (adapted from Budney & Higgings, 1998) + CM (Budney & Higgings, 1998)	CRA	CM		Delivery
Substance use disorders	CRA (Sisson & Azrin, 1989) + CM (Higgings, 1993, 1994)	CRA	CM		
Substance use disorders	CBC (Bux, 1998; Platt, 1991) + CM (Stitzer, 1992)	CBT	CM		
Substance use disorders	CM+BDT (Milby, 2008, no manual)	CM	CBT		
Substance use disorders	CBT (adapted from Carroll, 1998) + CM (Petry 2000)	CBT	CM		Unknown
Substance use disorders	CBT (Rawson, 1989, 1995) + CM (Higgins, 1991, 1993)	CBT	CM		
Substance use disorders	TSF (Nowinski & Baker, 1992) + CM (Higgins, 1993)	TSF	CM		
Substance use disorders	CT/CBT (Beck, 1993) + GDC (Daley, 2002)	CBT	DC		
Substance use disorders	SEPT (Mark & Faude, 1995, unpublished) + GDC (Mercer, 1994)	SUP	DC		
Substance use disorders	CBT (Rawson, 1995) + CM (adapted from Roll, 1996)	CM	CBT		Content

List of acronyms for interventions and families: AP: Abandonment psychotherapy. BDT: Behavioral Day Treatment. CBC: Cognitive-behavioral counseling. CBCST: Cognitive behavioral coping skills treatment. CBT: Cognitive-behavioral therapy. CBT-AN: Cognitive-behavioral therapy for Anorexia Nervosa. CCT: Client-Centered therapy. CM: Contingency management. CRA: Community reinforcement approach. CT: Cognitive therapy. CT/CBT: Cognitive therapy/Cognitive-behavioral therapy. CTBE: Community treatment by experts. DBT: Dialectical behavior therapy. DBT-E: Dialectical Behavioral Therapy enhanced with psychodynamic techniques. DBT-SK: Dialectical behavior therapy - skills training. DC: Drug Counselling. ERP: Exposure and response prevention. FEST: Emotionally demanding, Supportive, Psychoeducational psychotherapy. FT: Family therapy. FUN: Functional analysis. GDC: Group Drug Counselling. GET: Gruppi Esperienziali Terapeutici. GPI: Group Psychological Intervention. ICM: Intensive clinical management. IFPT: Individually Focused Patient Treatment. LEAP: Compulsive Exercise Activity Therapy. MAAP: Mechanism-based anti-aggression psychotherapy. MACT: Manual assisted cognitive treatment. PSED: Psychoeducation. PSYD: Psychodynamic psychotherapy. REL: Relaxation. SCH: Schema Therapy. SEPT: Supportive-expressive psychodynamic therapy. SSCM: Specialist Supportive Clinical Management. SST: Social skills training. SUP: Supportive therapy. TSF: Twelve-Step facilitation.

Table S12. Manuals of distinct interventions used across more than one disorder

Manual	Full citation	Disorders	Trials sharing manual
Fairburn 1993 (CBT-BN)	Fairburn CG, Marcus MD, Wilson GT (1993). Cognitive behavioral therapy for binge eating and bulimia nervosa: a comprehensive treatment manual. In: Fairburn CG, Wilson GT (eds.) Binge Eating: Nature, Assessment, and Treatment. New York, NY: Guilford Press: 361-404.	Anorexia, Bulimia	Anorexia: McIntosh 2005<br>Bulimia: Agras 2000; Fairburn 1993; Ghaderi 2004; Goldbloom 1997; Mitchell 2008.
Fairburn 2008 (CBT-E)	Fairburn CG, Cooper Z, Shafran R, Bohn K, Hawker DM, Murphy R, Straebler S (2008). Enhanced cognitive behavior therapy for eating disorders: the core protocol. In: Fairburn CG (ed.) Cognitive Behavior Therapy and Eating Disorders. New York: Guilford.	Anorexia, Bulimia	Anorexia: Byrne 2017; Zipfel 2014 (ANTOP)<br>Bulimia: Fairburn 2009; Fairburn 2015; Thompson-Brenner 2016
Klerman 1984 (IPT for Depression)	Klerman GL, Weissman MM, Rounsaville BJ, Chevron ES (1984). Interpersonal Psychotherapy of Depression. New York: Basic Books.	Anorexia, Bulimia	Anorexia: McIntosh 2005<br>Bulimia: Fairburn 1993
Bulik 1993 (CBT-BN, unpublished)	Bulik C, Sullivan PF, Carter FA, Joyce PR (1993). Cognitive Therapy Therapist Manual for the Treatment of Bulimia Nervosa. Unpublished.	Anorexia, Bulimia	Anorexia: McIntosh 2005<br>Bulimia: Bulik 1998
Linehan 1993 (DBT Skills)	Linehan MM (1993). Skills Training Manual for Treating Borderline Personality Disorder. New York: Guilford Press.	Bipolar, Borderline	Bipolar: Van Dijk 2013.<br>Borderline: Koons 2001; Lin 2019; Linehan 2006; McMain 2009; Pistorello 2012; Priebe 2012; Soler 2009; Walton 2020.
Young 1994 (Schema-Focused Therapy)	Young JE (1994). Cognitive Therapy for Personality Disorders: A Schema-Focused Approach (2nd ed.). Sarasota, FL: Professional Resource Press.	Anorexia, Borderline	Anorexia: Ball 2004.<br>Borderline: Giesen-Bloo 2006.
Fawcett 1987 (Clinical Management — NIMH TDCRP)	Fawcett J, Epstein P, Fiester SJ, Elkin I, Autry JH (1987). Clinical management — imipramine/placebo administration manual. Psychopharmacology Bulletin, 23, 309-324.	Bipolar, Bulimia, Substance (stimulant) use disorder	Bipolar: Frank 2005<br>Bulimia: Goldbloom 1997<br>SUD: Carroll 1994; Carroll 1998

Table S13. Number of distinct intervention arms in each macro-family grouped by disorder

Intervention family acronym	Anorexia nervosa [a]	Bipolar disorder [b]	Borderline personality disorder [c]	Bulimia nervosa [d]	Schizophrenia and psychotic [e]	Substance use disorder [f]
CBT	6	16	9	16	23	19
CM						22
PSED		17			9	
PSYD	5		16		1	
SUP		4	8	1	7	3
CR	1				13	
DBT		2	11			
CRA						4
SCR					8	
SST					7	
FT	2	5			2	
CLIN		5				1
TSF						5
SHP				5		
IPT	1		1	3		
ERP				4		
MCT					3	
DC						4
SCH			4			
IPSRT		3				
SSCM	1					
CAT	1				2	
REL		1		1	1	
MANTRA	1					
MF-PSED					1	
BFT	1				1	
PS					1	
SCM			1			
COPE					1	
EMDR					1	
FR		1				
FUN				1		
GSD					1	
HHS					1	
HIT					1	
ILLMNG					1	
LEAP	1					
MBT						1
MFT					1	
NSDG					1	
NSIT	1					
P-OT					1	
PP					1	
PSYA	1					
PSYSOC					1	

Note.

List of acronyms: BFT: Behavioral family therapy; CAT: Cognitive analytic therapy; CBT: Cognitive behavioral therapy; CLIN: Clinical intervention; CM: Contingency management; COPE: Coping skills; CR: Cognitive remediation; CRA: Community reinforcement approach; DBT: Dialectical behavior therapy; DC: Drug counselling; EMDR: Eye movement desensitization and reprocessing; ERP: Exposure and response prevention; FR: Functional remediation; FT: Family therapy; FUN: Functional analysis; GSD: Guided self-determination; HHS: Holistic health sessions; HIT: Hallucinations focused integrative treatment; ILLMNG: Illness management; IPSRT: Interpersonal and social rhythm therapy; IPT: Interpersonal psychotherapy; LEAP: Compulsive exercise activity therapy; MANTRA: Maudsley model of anorexia nervosa treatment for adults; MBT: Mind-Body Therapy; MCT: Metacognitive training; MF-PSED: Mindfulness-based psychoeducation; MFT: Multifamily treatment ; NSDG: Non-specific discussion group; NSIT: Nonspecialistic Intervention; P-OT: Psychological based occupational therapy; PP: Positive psychotherapy; PS: Problem Solving; PSED: Psychoeducation; PSYA: Psychoanalysis; PSYD: Psychodynamic psychotherapy; PSYSOC: Psychosocial intervention; REL: Relaxation; SCH: Schema therapy; SCM: Structured clinical management; SCR: Social cognitive remediation ; SHP: Self-help; SSCM: Specialistic supportive clinical management; SST: Social skills training; SUP: Supportive therapy; TSF: Twelve-Step facilitation

[a] 2 distinct interventions were assigned to multiple families and were counted in each relevant family row

[b] 6 distinct interventions were assigned to multiple families and were counted in each relevant family row

[d] 9 distinct interventions were assigned to multiple families and were counted in each relevant family row

[e] 6 distinct interventions were assigned to multiple families and were counted in each relevant family row

[e] 4 distinct interventions were assigned to multiple families and were counted in each relevant family row

[f] 12 distinct interventions were assigned to multiple families and were counted in each relevant family row

Tomei G, *et al. BMJ Ment Health* 2026; 29:e302555. doi: 10.1136/bmjment-2026-302555

Table S14. Adaptations of interventions

Type of adaptation	Sub-type of adaptation	N (% [a])	Anorexia (n)	Bipolar (n)	Borderline (n)	Bulimia (n)	Schizophrenia and psychotic (n)	Substance use disorder (n)
Mod. Content (n)		59 (43)	4	9	14	11	13	8
	Modified component (n)	46 (34)	4	5	11	10	13	3
	Adapted for population (n)	12 (9)		4	2	1		5
	Unspecified (n)[b]	1 (1)			1			
Mod. Delivery (n)		14 (10)	1	2	5	4		2
	Format (n)	8 (6)	1	1	1	3		2
	Setting (n)	1 (1)			1			
	Mode (n)	4 (3)		1	2	1		
	Unspecified (n)[b]	1 (1)			1			
Mod. Structure (n)		42 (31)		18	15	1	7	1
	Altered number of sessions (n)	30 (30)		10	11	1	7	1
	Altered frequency of sessions (n)	2 (1.5)		2				
	Altered length of sessions (n)	9 (7)		5	4			
	Altered internal structure of sessions (n)	1 (1)		1				
Mod. Cultural (n)		9 (7)		1			8	
Mod. Unknown (n)		13 (9.5)	2			1	2	8
Total		137	7	30	34	17	30	19

Note. [a] Percentages calculated out of the total number of adaptations (n=137)
[b] For one trial arm, original authors explicitly reported content and delivery adaptations but did not provide further details

Table S15. Cultural adaptations

Trial (Author Year)	Disorder	Cultural adaptation
Nagy 2015	Bipolar	Translated to Arabic and adapted to Egyptian culture.
Gohar, 2013	Psychosis	Translated to Arabic and adapted to Egyptian culture.
Liberman 2009	Psychosis	Adapted to Latinos in California
Li 2015	Psychosis	Translated and adapted to Chinese culture
Naeem 2015	Psychosis	Translated and adapted to Pakistani culture
Rathod 2013	Psychosis	Adapted to Black British, African Caribbean/Black African and South Asian Muslim participants.
Tas 2012	Psychosis	Translated and adapted to Turkish culture
Vita 2011a	Psychosis	Translated and adapted to Italian culture
Vita 2011b	Psychosis	Translated and adapted to Italian culture

Tomei G, *et al. BMJ Ment Health* 2026; 29:e302555. doi: 10.1136/bmjment-2026-302555
